# Genome-Wide Identification and Functional Analysis of *RF2* Gene Family and the Critical Role of *GhRF2-32* in Response to Drought Stress in Cotton

**DOI:** 10.3390/plants12142613

**Published:** 2023-07-11

**Authors:** Haonan Gu, Zilin Zhao, Yangyang Wei, Pengtao Li, Quanwei Lu, Yuling Liu, Tao Wang, Nan Hu, Sumei Wan, Baohong Zhang, Shoulin Hu, Renhai Peng

**Affiliations:** 1 College of Agriculture, Tarim University, Alar 843300, China; 2 Anyang Institute of Technology, Anyang 455000, China; 3 Department of Biology, East Carolina University, Greenville, NC 27858, USA

**Keywords:** cotton, transcription factor, *RF2* gene, drought

## Abstract

Cotton is an important natural fiber crop. The *RF2* gene family is a member of the bZIP transcription factor superfamily, which plays an important role in plant resistance to environmental stresses. In this paper, the *RF2* gene family of four cotton species was analyzed genome-wide, and the key gene *RF2-32* was cloned for functional verification. A total of 113 *RF2* genes were identified in the four cotton species, and the *RF2* family was relatively conserved during the evolution of cotton. Chromosome mapping and collinear analysis indicated that fragment replication was the main expansion mode of *RF2* gene family during evolution. *Cis*-element analysis showed that there were many elements related to light response, hormone response and abiotic stress response in the promoters of *RF2* genes. The transcriptome and qRT-PCR analysis of *RF2* family genes in upland cotton showed that *RF2* family genes responded to salt stress and drought stress. GhRF2-32 protein was localized in the cell nucleus. Silencing the *GhRF2-32* gene showed less leaf wilting and increased total antioxidant capacity under drought and salt stress, decreased malondialdehyde content and increased drought and salt tolerance. This study revealed the evolutionary and functional diversity of the *RF2* gene family, which laid a foundation for the further study of stress-resistant genes in cotton.

## 1. Introduction

Plants are affected by a variety of abiotic stresses in the process of growth and development, which leads to a substantial reduction in crop yield [1]. Drought, salt and low temperature are the most common abiotic stresses in nature. About 1/3 of the land in the world is in an arid or semi-arid environment [2], resulting in the degradation of natural vegetation and serious damage to the structure and function of the ecosystem [3]. Saline-alkalized land slows reduced plant growth and development as well as yield and quality by affecting seed germination, root length, plant height and fruit setting rate [4,5]. Under low temperature stress, the related physiological and metabolic activities of plant cells are affected and even lead to plant death [6]. Thus, it is extremely important to study the mechanism of plant response to abiotic stress and to identify the related genes for crop improvement.

Transcription factors, also known as trans-acting elements, are a class of proteins that specifically bind to *cis* elements in the promoter region of eukaryotic genes, thus specifically activating or inhibiting the expression of related genes at a spatiotemporal manor [7]. The bZIP transcription factors are some of the most widely studied transcription factor gene families, which play an extremely important role in plant growth and development as well as response to abiotic stress [8]. There are many members of the bZIP transcription factor family. There are 78 *bZIP* genes in *Arabidopsis thaliana*, which are divided into 13 subfamilies [9]. The *bZIP* gene family has been identified in many species, including *Glycine max* [10], *Brassica napus* [11], *Oryza sativa* [12] and corn [13].

Two RF2-like bZIP transcription factors, RF2a and RF2b, were isolated and identified for the first time in rice via interaction with the vascular tissue-specific *cis*-element Box II, which activated the promoter expression of DNA virus rice tungro bacilliform virus (RTBV) [14,15]. All identified *RF2* genes belong to subgroup I of 13 subfamilies of bZIP transcription factors [12] and are involved in the development of vascular system and the formation of symptoms of rice RTBV [15]. In previous studies, an RF2-like bZIP transcription factor gene *TabZIP3* was cloned from wheat by RACE, which is a homologue to RF2b. TabZIP3 was induced via salt and drought stress, and its overexpression improved the salt tolerance of transgenic *A. thaliana*, indicating that RF2-like bZIP transcription factors also play an important role in abiotic stress [16].

As one of the main raw materials of textiles, cotton is one of the most important economic and oil crops [17,18]. As the climate changes, environmental stresses, including salinity and drought stresses, have become a major issue seriously affecting cotton yield and quality [19]. Although cotton is considered to be a salt-tolerant crop, it can also be adversely affected by abiotic stress, especially at seedling emergence and seedling stage [20]. Although 207 *bZIP* genes have been identified in cotton [21]; the number of RF2 genes in different cotton species is not clear, and the function of the cotton *RF2* gene has not been reported. In this study, we first identified all*RF2* genes in four cotton species genome-wide, then we systemically analyzed the physical and chemical property of these genes, including their structures and expression profiles in different conditions. We also studied their evolutionary relationship during cotton evolution. Finally, we employed VIGS technology to study the function of these RF2 genes. This study laid a theoretical foundation for the further analysis of the function of *RF2* gene and provided a reference for cotton breeding.

## 2. Results

### 2.1. Identification and Physicochemical Property Analysis of RF2 Gene Family in Cotton

A total of 113 *RF2* genes were identified in the four cotton species via homologous search using the Markov Model (PF00170 and PF07716) and specific conserved domain (269851-bZIP_plant_RF2) of *RF2* genes (Table S2). A total of 36 and 39 *RF2* genes were found in *G. hirsutum* and *G. barbadense*, and 20 and 18 *RF2* genes were found in *G. arboretum and G. raimondii*, respectively. According to the position of *RF2* gene on chromosome in each cotton species, all identified genes were renamed, such as *G. hirsutum* (*GhRF2-1*).

The results of physicochemical properties analysis showed that the amino acid length of cotton *RF2* gene protein ranged from 113 (*GbRF2-36*) to 572 (*GbRF2-4*), the predicted molecular weight range was 13,220.02 (*GbRF2-36*)–62,572.12 (*GrRF2-4*) and the isoelectric point range was 5.38 (*GrRF2-8*)–9.17 (*GaRF2-5*). The instability index refers to how stable the protein was in the test tube (≤40, possibly stable; >40, possibly unstable). The prediction results showed that the instability coefficient ranges from 35.5 (*GbRF2-36*) to 73.98 (*GhRF2-36*). Except for *GbRF2-10*, *GaRF2-12* and *GbRF2-36*, all *RF2* genes may be unstable. Subcellular prediction results showed that all genes were located in the nucleus, but eight genes may also be located in chloroplast (Table S2).

### 2.2. Construction of Phylogenetic Tree of RF2 Genes in Cotton

**Figure 1:**
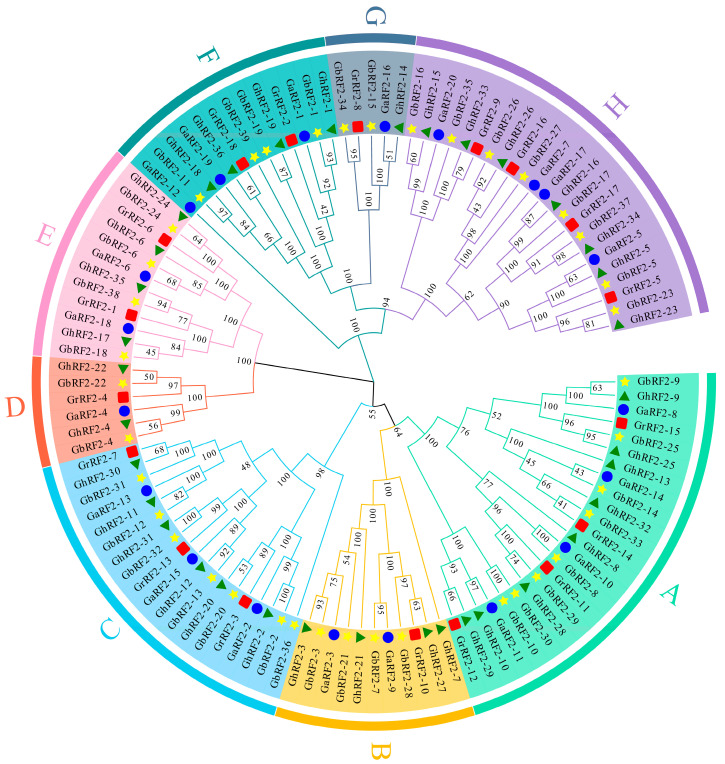
Phylogenetic tree of *RF2* gene in four cotton species. The arcs of different colors represent *RF2* genes of different subfamilies. Yellow stars represent *G. barbadense*, red squares represent *G. raimondii*, blue circles represent *G. arboretum* and green triangles represent *G. hirsutum*. A–H: Classification of *RF2* gene family.

In order to study the evolutionary relationship of the cotton *RF2* gene family, we sequenced the RF2 proteins of four cotton species and constructed a phylogenetic tree (Figure 1). According to the results, all family members are divided into eight subfamilies, of which subgroup A contains the largest number of genes, namely twenty-four genes, while subgroup G has the least number of genes, only five genes. The results showed that each branch contained the genes of four cotton species, and we found that the number of tetraploid cotton in each subgroup was about 2:1 compared with the number of diploid cotton. For example, D subgroup contained two genes from *G. hirsutum*, two genes from *G. barbadense*, one gene from *G. arboretum* and one gene from *G. raimondii*. This also fully showed the evolutionary relationship among the four cotton species. We also found that the *RF2* genes of *G. hirsutum* and *G. barbadense* were always clustered together, which may be due to the occurrence of gene repetition events.

### 2.3. Analysis of RF2 Gene Conserved Motif and Exon–Intron Distribution Pattern in Cotton

**Figure 2:**
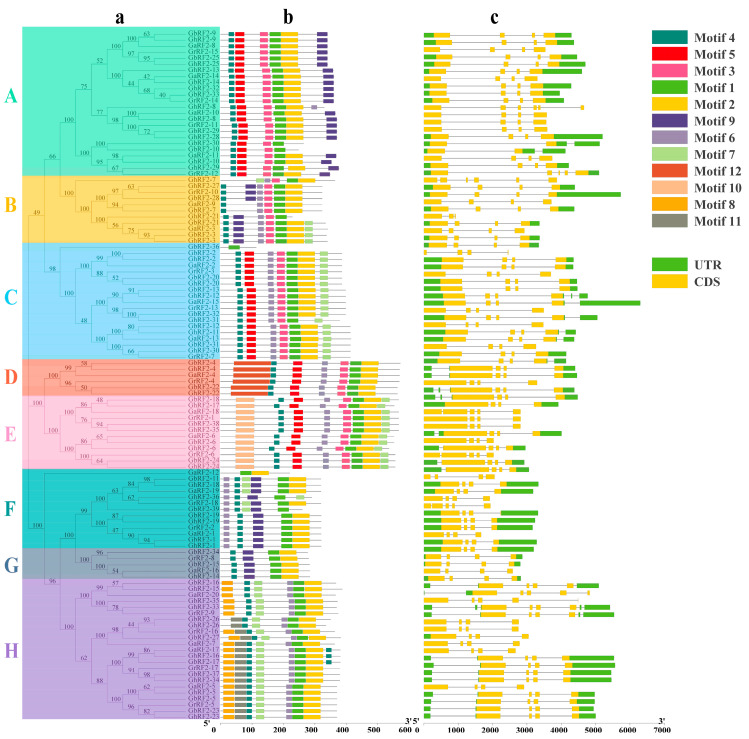
Structural triple diagram of *RF2* family in cotton. (**a**) Phylogenetic relationship of proteins encoded by RF2 in cotton. (**b**) Conserved motif of *RF2* in cotton. (**c**) Conserved domains of proteins encoded by RF2 in cotton. A–H: Classification of *RF2* gene family.

The information related to the evolution process of gene family can be obtained by analyzing conservative motif. Twelve conserved motif of cotton RF2 proteins were analyzed using the MEME online tool (Figure 2b). The results showed that all cotton *RF2* gene encoded proteins had motif 1. All but seven genes contained motif 2. This demonstrated that its structure was highly conservative. The *GbRF2-36* gene of subgroup C contained only one motif of motif 1. The *GaRF2-12* gene of subfamily F contained two motifs, including motif 1 and motif 2. Other conserved motifs existed only in one or some specific subfamilies, for example, motif 5 existed in A, C, D, and E subfamilies. Motif 12 existed only in the D subfamily. There were different conserved motifs in different subfamilies. In the same subfamily, the distribution of protein conserved motifs was basically the same, indicating that the gene function of the same subfamily was similar, and the homologous relationship was closed, which also proved the accuracy of the constructed phylogenetic tree.

The gene structure analysis of cotton *RF2* showed that the number of exons and introns of cotton *RF2* gene varied, with a minimum of two exons and a maximum of six exons (Figure 2c). Most *RF2* genes contained four exons and three introns. *RF2* genes in the same subfamily had very similar exon and intron structures. For example, all genes of C, D and G subfamilies contained four exons and three introns. This showed that the gene structure from the same subgroup was highly conservative and was closely related to evolution.

### 2.4. Distribution of RF2 Gene Family on Chromosomes in Cotton

**Figure 3:**
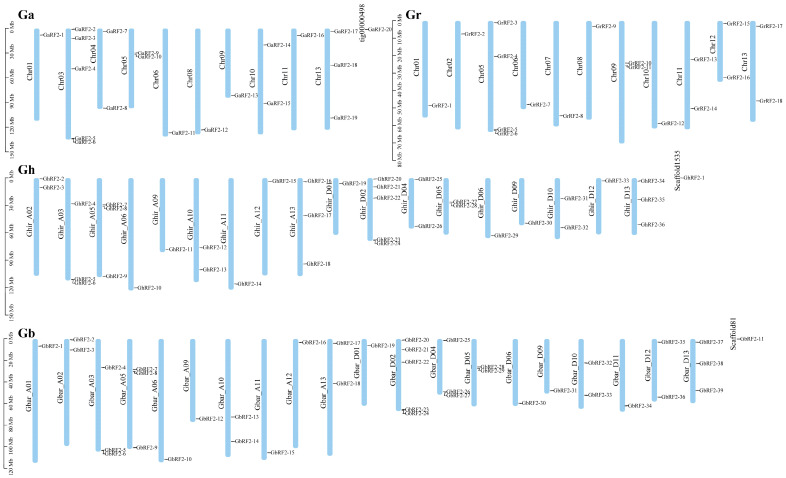
Distribution of *RF2* gene on chromosomes in cotton. The vertical bar on the left represented the size of the chromosome in Mb, and the chromosome number was on the left side of each chromosome. The corresponding cotton was marked in the upper left corner of the picture.

We combined the chromosomes of diploid A genome *G. arboretum*, diploid D genome *G. raimondii* and tetraploid A, D genome *G. hirsutum* and *G. barbadense* and the location information of each *RF2* gene on the chromosome to draw the distribution map of *RF2* gene on the chromosome (Figure 3). A total of 19 genes were distributed on 10 chromosomes of A genome and 18 genes on 11 chromosomes of D genome. The A subgenome of tetraploid contained 17 *RF2* genes, while in D subgenome, *G. hirsutum* contained 18 *RF2* genes and *G. barbadense* contained 21 *RF2* genes. One gene was located on the scaffold in the genomes of *G. arboretum*, *G. hirsutum* and *G. barbadense*.

### 2.5. Intraspecific and Interspecific Collinearity Analysis of RF2 Gene in Cotton

**Figure 4:**
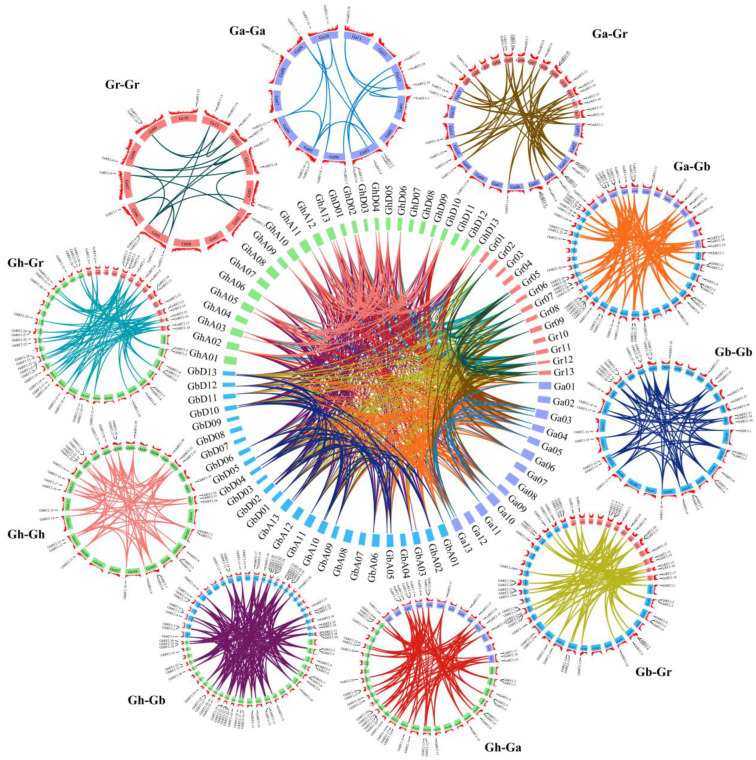
Collinearity of repetitive gene pairs of *RF2* gene in four cotton species. The chromosomes of different cotton species were marked with different colors, and the lines of different colors represented the contrastive relationship within and among different cotton species.

Gene duplication is the main force for promoting the expansion of the gene family. Gene duplication consists of tandem duplication and large fragment duplication [22]. The number of *RF2* genes in tetraploid cotton was twice as much as that in diploid cotton, which indicated that the *RF2* gene family expanded during cotton polyploidy. In order to study the expansion mode of *RF2* gene family in cotton, we analyzed the intraspecific and interspecific collinearity of four cotton species (Figure 4). The results showed that a total of 162 duplication gene pairs were obtained in the four cotton varieties, including 16 pairs in *G. arboretum*, 17 pairs in *G. raimondii*, 65 pairs in *G. hirsutum* and 64 pairs in *G. barbadense*. Of these, only *G. barbadense* contained one pair of tandem repeat genes. In addition, we comprehensively analyzed the collinearity among different cotton species and found that 556 duplication gene pairs existed in the following six groups of comparisons: Ga-Gr (49), Gb-Ga (90), Gb-Gr (84), Gh-Ga (89), Gh-Gb (157) and Gh-Gr (87). These results show that gene duplication, especially fragment duplication, plays an important role in the expansion of cotton *RF2* gene family.

### 2.6. Analysis of Cis Elements of RF2 Gene Promoters in Cotton

**Figure 5:**
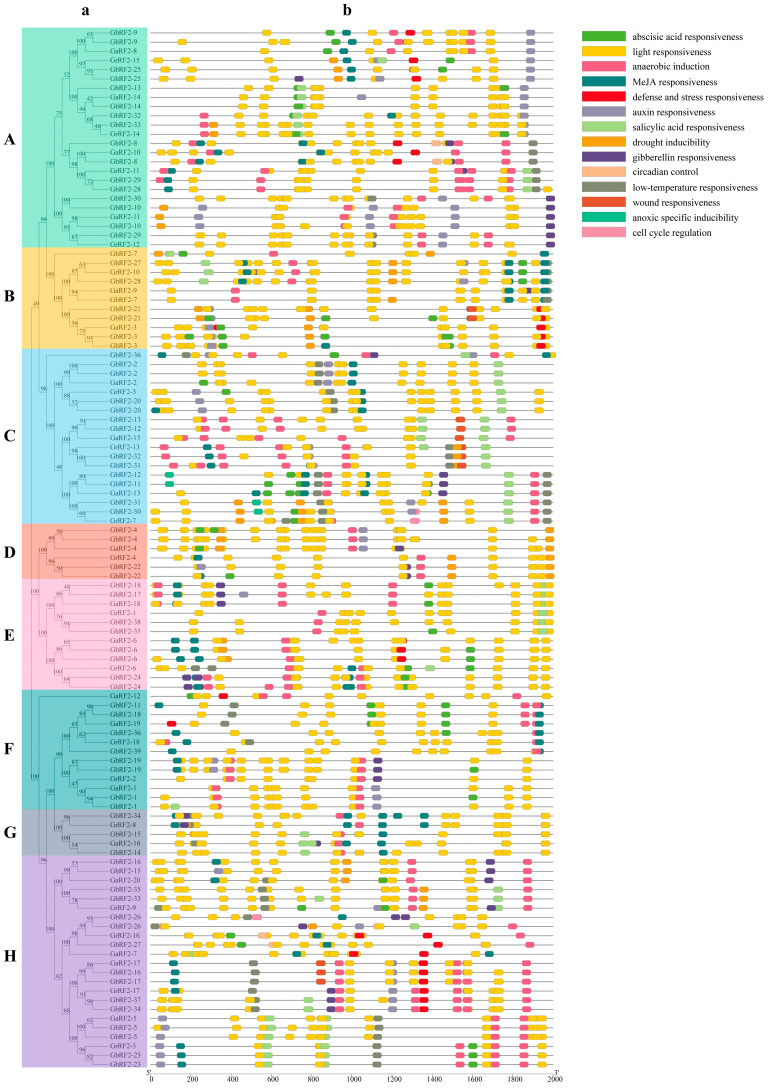
*Cis* elements in the promoter of cotton *RF2* Gene. Different colors represent different response elements. A–H: Classification of *RF2* gene family. (**a**) phylogenetic tree of all identified *RF2* genes in four cotton species. (**b**). *Cis* elements in the promoters of cotton *RF2* Genes.

In order to better understand the function of cotton *RF2* gene, we used the PlantCARE website to analyze the upstream 2000 bp region of the gene coding region and obtain regulatory *cis* elements. The results showed that the number of light response elements of cotton *RF2* gene was the largest (Figure 5). Certain hormone-related response elements were also obtained, including abscisic acid, MeJA, auxin, salicylic acid and gibberellin. A different number of *cis* elements associated with the defensive stress response were identified. We also found that the *RF2* gene may be related to the circadian rhythm and cell cycle. At the same time, there were a certain number of genes associated with abiotic stress, such as drought and low temperature. To sum up, different types and numbers of cis-acting elements were distributed in cotton *RF2* promoters. We analyze the function of RF2 gene under abiotic stress in the next step.

### 2.7. RF2 Gene Expression Patterns in Cotton under Abiotic Stress

**Figure 6:**
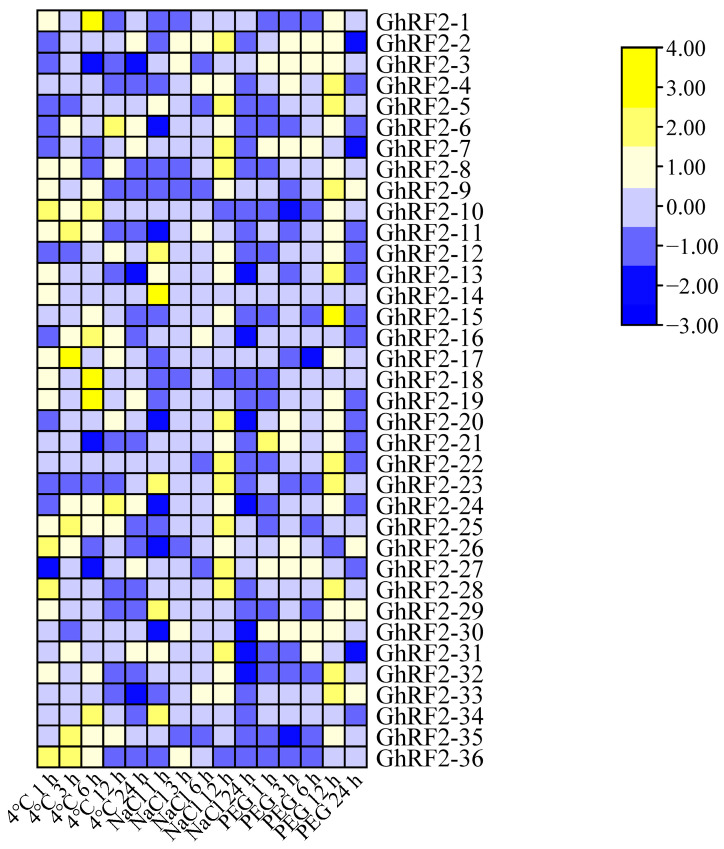
Expression of *RF2* gene in upland cotton under different stress conditions.

In order to explore whether cotton *RF2* genes were involved in cotton response to different abiotic stresses, we further analyzed the expression of *RF2* genes in upland cotton exposed to drought, salt and low temperature stresses. The results showed that the expression of *RF2* genes changed under different abiotic stresses and different treatment times (Figure 6) (Table S3). Under low temperature stress, 14 candidate genes (*GhRF2-2*, *GhRF2-3*, *GhRF2-4*, *GhRF2-6*, *GhRF2-16*, *GhRF2-17*, *GhRF2-20*, *GhRF2-21*, *GhRF2-22*, *GhRF2-23*, *GhRF2-24*, *GhRF2-27*, *GhRF2-32* and *GhRF2-35*) were significantly up-regulated by 4 to 40 times. Under salt stress, 15 candidate genes (*GhRF2-2*, *GhRF2-3*, *GhRF2-4*, *GhRF2-5*, *GhRF2-6*, *GhRF2-16*, *GhRF2-17*, *GhRF2-20*, *GhRF2-21*, *GhRF2-22*, *GhRF2-23*, *GhRF2-24*, *GhRF2-27*, *GhRF2-32* and *GhRF2-35*) were significantly up-regulated by about 4 to 20 times. Under drought stress, 12 candidate genes (*GhRF2-2*, *GhRF2-3*, *GhRF2-4*, *GhRF2-5*, *GhRF2-6*, *GhRF2-16*, *GhRF2-20*, *GhRF2-22*, *GhRF2-23*, *GhRF2-24*, *GhRF2-32* and *GhRF2-35*) were significantly up-regulated by about 5 to 25 times. We selected 12 key genes from 36 differentially expressed genes in upland cotton for further experiments, including: *GhRF2-2*, *GhRF2-3*, *GhRF2-4*, *GhRF2-5*, *GhRF2-6*, *GhRF2-16*, *GhRF2-20*, *GhRF2-22*, *GhRF2-23*, *GhRF2-24*, *GhRF2-32* and *GhRF2-35*.

### 2.8. qRT-PCR Analysis of RF2 Genes in Upland Cotton under Different Abiotic Stresses

**Figure 7:**
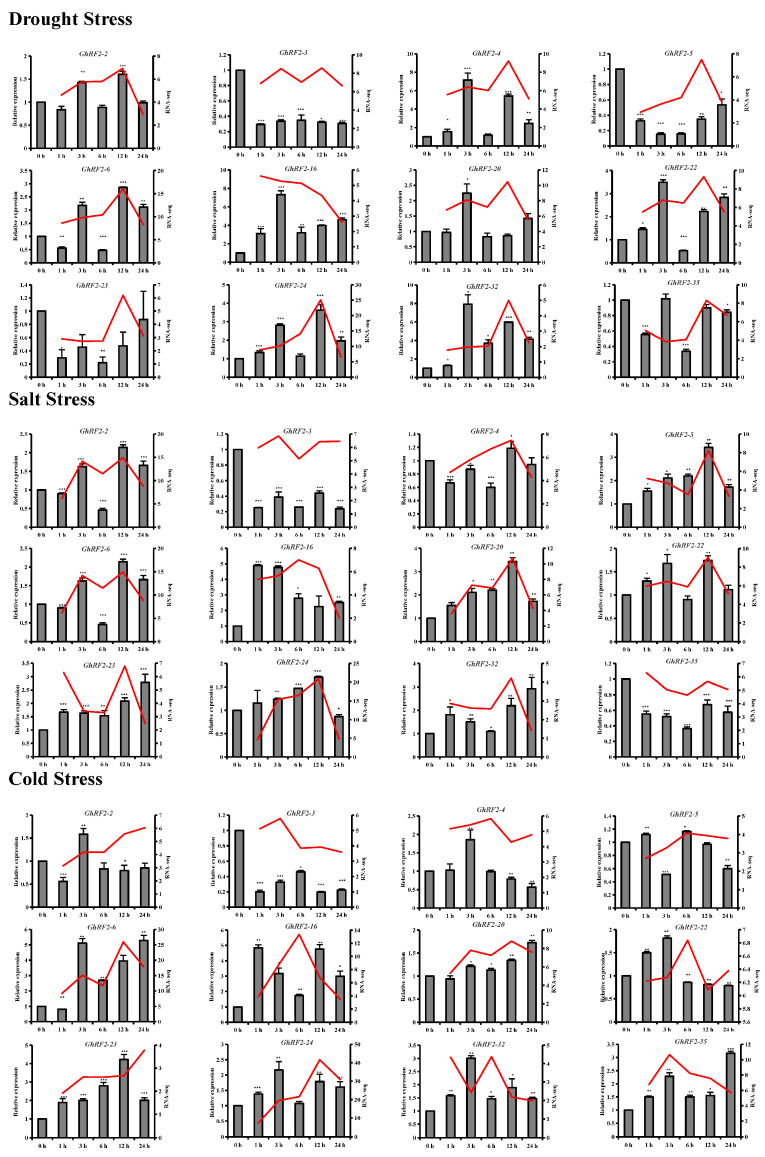
Expression patterns of 12 key *RF2* genes under salt, drought and cold stress.*: *p* < 0.05, **: *p* < 0.01,***: *p* < 0.001, Student’s *t*-test.

Under drought stress, *GhRF2-4*, *GhRF2-16* and *GhRF2-32* were significantly up-regulated, while *GhRF2-3*, *GhRF2-5* and *GhRF2-23* were significantly down-regulated. In addition, *GhRF2-2*, *GhRF2-6*, *GhRF2-20*, *GhRF2-22* and *GhRF2-24* were up-regulated and *GhRF2-35* was down-regulated under drought stress. Under salt stress, *GhRF2-5*, *GhRF2-16*, *GhRF2-20*, *GhRF2-23* and *GhRF2-32* were significantly up-regulated, while *GhRF2-3* and *GhRF2-35* were significantly down-regulated. Under cold stress, *GhRF2-6*, *GhRF2-16*, *GhRF2-23*, *GhRF2-32* and *GhRF2-35* were significantly up-regulated, while *GhRF2-3* was significantly down-regulated (Figure 7). We also found that the expression of *GhRF2-32* was significantly up-regulated under all tested abiotic stresses (drought stress, salt stress and low temperature stress), which will be further investigated.

### 2.9. Analysis of the Subcellular Localization of GhRF2-32

**Figure 8:**
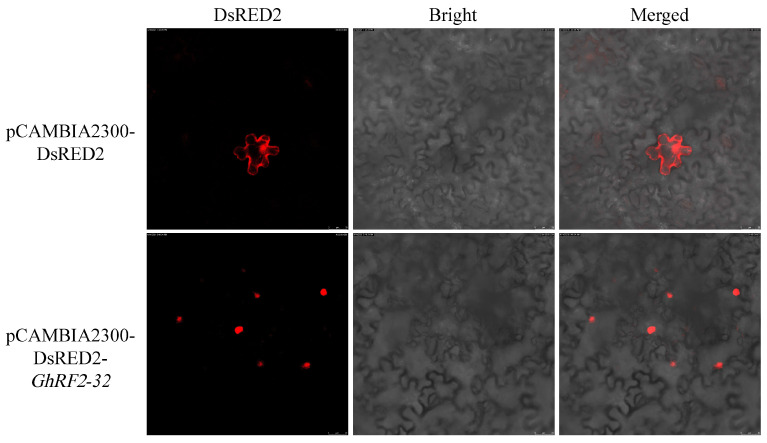
Subcellular localization of pCAMBIA2300-DsRED2-GhRF2-32 fusion protein.

In order to determine the subcellular localization of the *GhRF2-32* gene, the fusion expression vector pCAMBIA2300-DsRED2-*GhRF2-32* was constructed and transformed into GV1301 for infecting tobacco plants. As shown in Figure 8, pCAMBIA2300-DsRED2 (empty vector) displayed strong fluorescent signals in the cell membrane and nucleus of tobacco epidermal cells, but pCAMBIA2300-DsRED2-*GhRF2-32* was detected only in the nucleus, which confirmed that *GhRF2-32* was expressed in the nucleus.

### 2.10. Virus-Induced Gene Silencing (VIGS) of GhRF2-32 in Upland Cotton

**Figure 9:**
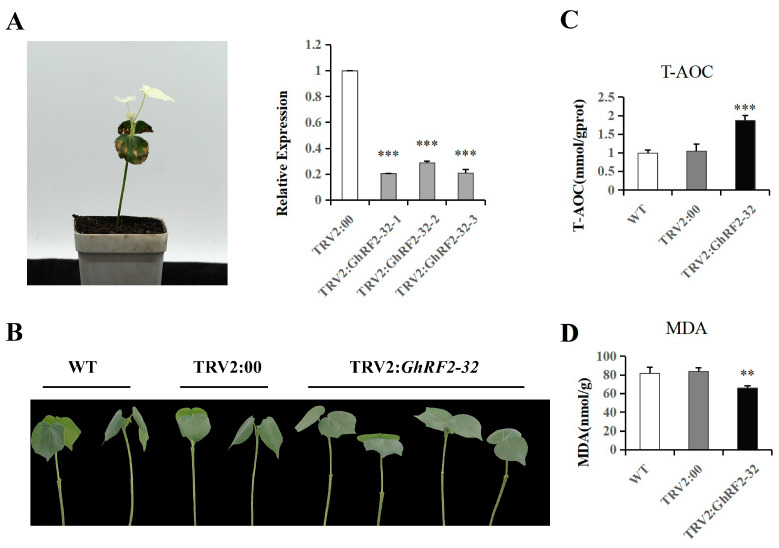
Silencing of the *GhRF2-32* gene by VIGS and the analysis of T-AOC and MDA contents under drought stress. (**A**) A positive control plant. (**B**) Phenotype of leaves in comparison with CK: control; TRV2:00: negative control; and TRV2:*GhRF2-32*-containing leaves. (**C**,**D**) T-AOC and MDA contents in WT: control, TRV2:00 empty vector and the TRV2:*GhRF2-32* candidate gene containing plants. Asterisks indicate statistically significant differences between transgenic lines and their corresponding control plants.

Virus-induced gene silencing (VIGS) was used to study *GhRF2-32* gene function in upland cotton. In this study, the *pds* gene served as positive control, which encodes a gene associated with chlorophyll synthesis. Silencing the *pds* gene, cotton leaves could not synthesize chlorophyll and the new leaves undergo albinism at a later stage. The occurrence of an albino phenotype after infection with cotton plants reveals successful gene silencing (Figure 9A). Subsequently, randomly selected cotton leaves were subjected to fluorescence quantification, and the expression levels of TRV empty gene and *GhRF2-32* gene were verified via qRT-PCR. The *GhRF2-32* gene expression levels of the normal and control plants did not significantly change, while the expression of target genes in cotton plants significantly decreased after silencing, indicating that *GhRF2-32* was successfully silenced (Figure 9A). An 18% PEG6000 solution was used to treat cotton plants with gene-silencing and empty vectors to simulate drought stress. Phenotypes were observed 72 h after drought stress, and the control cotton plants withered more severely than the gene-silenced plants (Figure 9B).

To study the potential mechanism of *GhRF2-32*-mediated drought tolerance, the T-AOC and MDA contents were measured in both normal control plants and VIGS-silenced plants (Figure 9C,D). For T-AOC, there was no significant difference between CK and TRV2:00 at 72 h after *GhRF2-32* gene silencing. However, in *GhRF2-32*-silenced seedlings, there was a significant difference between 12 and 24 h between the CK and TRV2:00-silenced plants, while there was no significant difference between 4 and 72 h between the CK and TRV2:00 plants. The results of MDA content were different from those of T-AOC. *GhRF2-32* gene showed significant differences from CK and TRV after 72 h of VIGS silencing. The MDA content in VIGS silenced plants was significantly lower than the controls. Based on the above results, it can be inferred that after the *GhRF2-32* gene was silenced, T-AOC content was increased and MDA content was decreased. This suggests that *GhRF2-32* functions in abiotic stress possibly through regulating active oxygen scavenging system.

## 3. Materials and Methods

### 3.1. Identification and Physicochemical Property Analysis of RF2 Gene Family in Cotton

The genomic sequences of *Gossypium hirsutum*, *Gossypium barbadense* [23], *Gossypium arboretum* [24] and *Gossypium raimondii* [25] were obtained from CottonMD [26]. Arabidopsis bZIP protein sequences were downloaded from Tair (https://www.arabidopsis.org/ (accessed on 12 August 2022)) and used as reference sequences to blast cotton genome using TBTools v1.106 [27]. Markov Model (HMM) PF00170 and PF07716 of the bZIP family were obtained from the Pfam (https://pfam.xfam.org/ (accessed on 12 August 2022)) [28]. The preliminary identification of the cotton bZIP family was conducted using the HMM Search of TBTools. The candidate proteins obtained by the two methods were merged and deduplicated. Then, NCBI Batch CD-Search was used to screen whether the candidate protein has the specific conserved domain (269851-bZIP_plant_RF2) of the RF2 family. The location information of the screened genes was extracted and further duplicated by the location information, and the final identification result was obtained. The sequence information of RF2 protein was obtained via TBTools, and the number of amino acids, relative molecular weight, isoelectric point and instability index were predicted using the ProtParam tool (https://web.expasy.org/protparam/ (accessed on 12 August 2022)) [29]. WoLF PSORT (https://wolfpsort.hgc.jp/ (accessed on 12 August 2022)) was used to predict the subcellular localization of the protein sequences of family members [30].

### 3.2. Phylogenetic Analysis of RF2 Gene in Cotton

Multiple sequence alignment of all cotton RF2 proteins was carried out via Clustal X, and the phylogenetic tree was constructed via the neighbor-joining method using MEGA-X [31]. The bootstrap was set to 1000 and model was selected as the p-distance. Finally, the evolution tree was modified and visualized by an online tool EvolView [32].

### 3.3. Analysis of Conserved Motifs and Gene Structures of RF2 Genes in Cotton

According to the location information of the extracted family members, the exon-intron structure of each gene was analyzed using TBTools, and the RF2 gene structure map was drawn. The conserved motif of RF2 protein sequence was identified via the online tool MEME (http://meme-suite.org/ (accessed on 12 August 2022)) [33]. The number of motifs was set to 12, the minimum length of motif was set to 10, the maximum length was set to 150, and other parameters were set by default. The output file in XML format was downloaded and visualized through TBTools.

### 3.4. Mapping RF2 Genes in Cotton Chromosome

According to the genome and genome annotation information, TBTools was used to extract the position of the *RF2* genes on the chromosome and the software was used to visualize the results.

### 3.5. Intraspecific and Interspecific Collinearity Analysis of RF2 Genes in Cotton

The collinearity analysis of *RF2* gene family sequences was performed using TBTools software, including fragment repeat, tandem repeat and genome-wide duplication events. Intraspecific and interspecific BLAST was performed on the diploid A/D genome (*G. arboretum*/*G. raimondii*) and tetraploid A/D subgenome (*G. hirsutum*/*G. barbadense*), respectively, and then the intraspecific duplicated gene pairs of *G. arboretum*, *G. raimondii*, *G. hirsutum* and *G. barbadense* and the interspecific duplicated gene pairs of *G. hirsutum*–*G. arboretum*, *G. hirsutum*–*G. raimondii*, *G. barbadense*–*G. arboretum*, *G. barbadense*–*G. raimondii* and *G. hirsutum*–*G. barbadense* obtained from duplication event analysis were used to map the intraspecific and interspecific covariance circles.

### 3.6. Prediction of Cis Elements of RF2 Gene in Cotton

According to the location information of *RF2* genes, the 2000 bp upstream DNA sequences of each RF2 gene were extracted from genome sequence files. The *cis* elements of the gene promoter region were predicted via PlantCARE (http://bioinformatics.psb.ugent.be/webtools/plantcare/html/ (accessed on 12 August 2022)) [34]. The predicted regulatory elements were visualized using TBTools.

### 3.7. Expression Pattern of RF2 under Abiotic Stress

The transcriptome data of *G. hirsutum* (PRJNA490626) under drought, salt and low temperature stress were downloaded from NCBI (https://www.ncbi.nlm.nih.gov/ (accessed on 12 August 2022)). First of all, the data were filtered using Trimmomatic software [35], the *G. hirsutum* reference genome database was built via the hisat2 tool [36], and then the transcriptome data were compared to the reference genome of the database. The FPKM values (fragments per kilobase of transcript per million fragments) of all genes contained in the genome were calculated by Cufflinks 2.0.2 software [37]. According to the unique ID combined transcriptome data of the proteome file corresponding to upland cotton *RF2* gene, the expression amount of each gene was obtained, the results were visualized and the expression heat map was drawn using TBTools.

### 3.8. Stress Treatments and qRT-PCR Analysis

*G. hirsutum* acc. TM-1 was grown in the experimental field at the Anyang Institute of Technology. In order to study the effects of drought, salt and low temperature stress on *RF2* gene expression in *G. hirsutum*, when the cotton seedlings grew to three true leaf stage, the seedlings were treated with 18% PEG, 250 mM NaCl and in a 4 °C incubator, respectively. The leaves of various stress treatments and the control group were collected at 0 h, 1 h, 3 h, 6 h, 12 h and 24 h, respectively. The leaves were immediately frozen in liquid nitrogen and stored at −80 °C. All treatments were repeated three times.

Total RNAs were extracted from each sample by using the EASYspin Plus Plant RNA Kit (RN38, Aidlab Biotech, Beijing, China). The quality of RNAs was determined via agarose gel electrophoresis and a Nanodrop2000 nucleic acid analyzer. mRNAs were reversely transcribed to cDNAs by using TranScript All-in-One First-Strand cDNA Synthesis SuperMix for qPCR (Transgen Biotech, Beijing, China). Finally, the qRT-PCR experiment was carried out on the ABI 7500 Fast Real-Time PCR system (Applied Biosystems, Waltham, MA, USA) using the TransStart Top Green qPCR SuperMix kit (Transgen Biotech, Beijing, China). *Actin* was saved as reference gene identified by RefFinder [38]. The primers of internal reference genes and differentially expressed genes in *G. hirsutum* were designed by the Prime-Blast of NCBI and listed in Table S1. Three biological replicates were run for each treatment and the control. Three repeats were performed on each cDNA sample, and the relative expression of the genes was calculated according to the CT value via the 2^−ΔΔCt^ method.

### 3.9. Subcellular Localization of GhRF2-32

According to the plasmid sequence of pCAMBIA2300-DsRED2 and the target gene *GhRF2-32* with the stop codon removed, primers were designed according to the specification, and BamHI was selected as the tangent point of line plasmid. The specific primers used were shown in Table S1. The PCR amplification product was ligated into the pCAMBIA2300-DsRED2 vector using a ClonExpress^®^ II One Step Cloning Kit, according to the manufacturer’s instructions. The recombinant plasmid was subsequently transformed into DH5α competent cells. The recombinant results were verified via PCR amplification and sequencing. Positive clones were screened, and plasmids were extracted to obtain the pCAMBIA2300-DsRED2-*GhRF2-32* vector construct. The plasmid was injected into leaves for 4 weeks under normal growth conditions. Two days later, the leaves injected with plasmid were removed and the red fluorescence of the DsRED2 fusion protein was observed using the Leica DMi8 confocal laser scanning microscope (Leica, Wetzlar, Germany).

### 3.10. Using Virus-Induced Gene Silencing (VIGS) to Test RF2 Gene Function

A 300 bp fragment of *RF2-32* gene was cloned into the TRV-RNA vector to develop the TRV2:*GhRF2-32* using the Kpn1 and Xba1 restriction sites. The primers were designed by SnapGene 4.1.9 software (Table S1). Similarly, TRV2:PDS was constructed as a visual marker to monitor silencing efficiency. As a negative control, TRV2:00 (empty vector) was used. All the vectors were inserted into the GV3101. We injected the cotyledons of 10 days old *G. hirsutum* seedlings at 25 °C. Th cotton plants with injected TRV2:PDS show an albino phenotype after 2 weeks of infiltration. When the cotton reaches the three-leaf stage, some of the plants were watered with water as the control and the others were treated with 18% PEG6000 solutions until the phenotypes appeared.

### 3.11. Determination of Enzyme Activity in Gene-Silencing Cotton after Drought Stress Treatment

The MDA content was measured using the Malondialdehyde (MDA) assay kit (BC0025, Solarbio, Beijing, China). The total antioxidant capacity (T-AOC) assay kit (A015-3-1, Nanjing Jiancheng Bioengineering Institute, Nanjing, China) was used to determine the T-AOC content. The experimental procedure was carried out in accordance with the instructions. During the measurement process, a spectrophotometer was used to determine the content of about 0.1 g of leaf samples from the wild type control and the *GhRF2-32-silenced* plants after 72 h of drought treatment. The experiment was repeated three independent times for three biological repetitions.

## 4. Discussion

Abiotic stress is a serious problem for plant growth and development, which threatens the growth and development of plants from all aspects, so it is urgent to study the response genes under stress. As an important economic crop in the world, cotton is also widely affected by almost all abiotic stresses, including drought, salt and high/low temperatures. *RF2* gene is an important gene belonging to the bZIP transcription factor superfamily. It has been identified as being associated with disease and abiotic stress in rice and wheat, but it has not been reported in cotton.

In this study, we identified a total of 113 *RF2* genes in four cotton species, including *G. hirsutum*, *G. barbadense*, *G. arboretum* and *G. raimondii* containing 36, 39, 20 and 18 *RF2* genes, respectively. Our results show that the number of *RF2* in tetraploid cotton is almost twice as much as that in diploid cotton, which confirms that allotetraploid cotton is produced via the natural hybridization of diploid cotton seeds containing A genome and D genome [39,40]. In the past, most of the results of family identification focused on the identification of bZIP superfamily, such as *A. thaliana* [41] and tobacco [42]. The physical and chemical properties of cotton RF2 protein showed that its sequence length, relative molecular weight and isoelectric point distribution were very wide. This may be related to the large-scale duplication of the genome of tetraploid cotton and the existence of a large number of *RF2* genes in tetraploid cotton. In this study, we predicted the subcellular location of cotton *RF2* and found that almost all genes were located on the nucleus, which was consistent with the results of previous studies. *RF2b* was clearly located on the nucleus in rice [15].

We constructed a phylogenetic tree of 113 RF2 proteins and divided them into eight subfamilies. Each subfamily contains four *RF2* genes of cotton species. At the same time, we found that the genes in the same subgroup had similar motif distribution patterns. The results of gene structure analysis also showed that *RF2* was highly conserved. The *RF2* of the same subfamily has similar exon/intron number and exon length.

In our study, we found that the distribution of *RF2* genes on each chromosome was uneven, but we found that most of the number and location of genes on each chromosome of the two tetraploid cotton species were one to one, this phenomena was also observed for other transcription factors in other plant species [43,44,45,46,47,48]. This may be due to the fact that allotetraploids share the common ancestors A0 (the possible extinct common ancestor of the existing A genome) and D5 (*G. raimondii*) [49].

*Cis* element analysis showed that *RF2* gene was associated with abiotic stress responses such as salt, drought and low temperature. Heat map analysis revealed that all *RF2* genes were expressed differently in upland cotton under different treatment times. Under salt stress, the growth of the *A. thaliana* lines overexpressing the *RF2* gene was significantly worse than that of the wild type, indicating that *TaRF2* (*TabZIP3*) gene played a positive regulatory role in plant salt stress response [17]. There are also many *bZIP* genes associated with abiotic stress. *OsbZIP71* specifically binds to the promoter of *OsNHX1* gene to enhance the salt stress resistance of rice by regulating cell osmosis [50]. In *A. thaliana*, *AtbZIP17* binds specifically to TGACG elements to regulate downstream salt stress response genes. The transcriptional activation state of *TabZIP2* increased in ploidy under severe drought conditions [51]. *ZmbZIP72* improved drought resistance in transgenic maize plants [52]. The overexpression of *TabZIP6* reduced the freezing resistance of transgenic *A. thaliana* seedlings [53]. *bZIP6* gene improved the freezing resistance of overexpressed plants in *A. thaliana* [54].

Drought is one of the important abiotic factors that restrict cotton yield and fiber quality. Traditional breeding methods are difficult to meet the needs of contemporary production, so it is important to identify and explore drought resistance-related genes in cotton. *RF2-32* gene was isolated from upland cotton TM-1. During the VIGS process of *GhRF2-32*, the emergence of an albino phenotype in plants indicates successful gene silencing. In addition, the expression of *GhRF2-32* was significantly reduced after silencing. After drought stress treatment, compared with the wild group plants and the control group plants, the silenced cotton plants showed a lighter wilt phenomenon. Gene silencing indicates that *GhRF2-32* has a negative regulatory effect on cotton drought resistance. There are various antioxidant macromolecules, small molecules and enzymes in plants. Their role is to eliminate various reactive oxygen species produced by plants under stress, thereby preventing the production of oxidative stress induced by reactive oxygen species. The total level of these antioxidant macromolecules, small molecules and enzymes reflects the total antioxidant capacity within the system. MDA is the final product produced by the decomposition of oxygen free radicals after acting on unsaturated fatty acids in lipids, and this reflects the level of lipid oxidation in plants. Drought stress can cause the accumulation of a large amount of reactive oxygen species, cause cell membrane lipid peroxidation and cause plant cell damage. Therefore, we evaluated the resistance of plants to environmental stress by measuring their total antioxidant capacity and MDA content under drought stress. The analysis after gene silencing showed that the total antioxidant capacity of cotton plants was significantly higher than that of the control cotton plants, and the content of malondialdehyde was significantly lower than that of non-silenced cotton plants, indicating that the metabolism in cotton leaves was slow, the degree of severity increased, which was consistent with the phenotype after drought treatment. It can be inferred that the silencing of the *GhRF2-32* gene improves the ability of cotton plants to eliminate the damage caused by reactive oxygen species, making them less susceptible to drought stress. Through our research on the function of *GhRF2-32* gene, including qRT-PCR, gene silencing techniques in cotton and the detection of physiological indicators after silencing, it can be inferred that *GhRF2-32* gene is involved in the regulation of cotton drought resistance.

## 5. Conclusions

Advanced genome sequencing and bioinformatics analysis have been bringing the related research into the next era of genome wide identification and gene function analysis [55]. In this study, a total of 113 *RF2* genes were identified in four different cotton species. The *RF2* family was analyzed at the evolutionary level by physical and chemical property analysis, phylogenetic tree construction, conservative motif analysis, gene structure analysis and collinearity analysis. The results showed that *RF2* genes were conserved in the process of evolution and expanded by gene duplication in different ploidy cotton species. Twelve key candidate genes were screened by heat map and qRT-PCR analysis, and *GhRF2-32* of the most significantly expressed core genes was identified for follow-up research. In the VIGS experiment, wild-type and control plants showed significant wilt compared to gene silenced cotton plants under the same drought treatment. The T-AOC of gene silenced cotton plants is higher than that of wild type and control group, and the MDA content is lower than that of wild type and control group, which indirectly indicates that *GhRF2-32* gene is related to drought resistance. Although the further functional analysis of these genes using the more advanced technologies may be required, such as CRISPR/Cas genome editing [56,57], this study lays a foundation for the mining of stress-resistant genes in cotton.

## Data Availability

All data were reported in this paper and will also available if requested.

## Supplementary Materials

The following supporting information can be downloaded at: https://www.mdpi.com/article/10.3390/plants12142613/s1, Table S1: Use NCBI designed differentially expressed gene-specific primer sequences; Table S2: Physico-chemical and biochemical characteristics of RF2 genes in cotton; Table S3: RNA-Seq data of RF2 gene family under different abiotic stress.

## References

[1] Valliyodan B., Nguyen H.T. (2006). Understanding regulatory networks and engineering for enhanced drought tolerance in plants. Curr. Opin. Plant Biol..

[2] Gupta A., Rico-Medina A., Caño-Delgado A.I. (2020). The physiology of plant responses to drought. Science.

[3] Yao Y., Fu B., Liu Y., Li Y., Wang S., Zhan T., Wang Y., Gao D. (2022). Evaluation of ecosystem resilience to drought based on drought intensity and recovery time. Agric. For. Meteorol..

[4] Liang W., Cui W., Ma X., Wang G., Huang Z. (2014). Function of wheat Ta-UnP gene in enhancing salt tolerance in transgenic Arabidopsis and rice. Biochem. Biophys. Res. Commun..

[5] Munns R., James R.A., Läuchli A. (2006). Approaches to increasing the salt tolerance of wheat and other cereals. J. Exp. Bot..

[6] Theocharis A., Clément C., Barka E.A. (2012). Physiological and molecular changes in plants grown at low temperatures. Planta.

[7] Corrêa L.G., Riaño-Pachón D.M., Schrago C.G., dos Santos R.V., Mueller-Roeber B., Vincentz M. (2008). The role of bZIP transcription factors in green plant evolution: Adaptive features emerging from four founder genes. PLoS ONE.

[8] Wang X., Lu X., Malik W.A., Chen X., Wang J., Wang D., Wang S., Chen C., Guo L., Ye W. (2020). Differentially expressed bZIP transcription factors confer multi-tolerances in *Gossypium hirsutum* L.. Int. J. Biol. Macromol..

[9] Dröge-Laser W., Snoek B.L., Snel B., Weiste C. (2018). The Arabidopsis bZIP transcription factor family-an update. Curr. Opin. Plant Biol..

[10] Zhang M., Liu Y., Shi H., Guo M., Chai M., He Q., Yan M., Cao D., Zhao L., Cai H. (2018). Evolutionary and expression analyses of soybean basic Leucine zipper transcription factor family. BMC Genom..

[11] Zhou Y., Xu D., Jia L., Huang X., Ma G., Wang S., Zhu M., Zhang A., Guan M., Lu K. (2017). Genome-Wide Identification and Structural Analysis of bZIP Transcription Factor Genes in Brassica napus. Genes.

[12] Nijhawan A., Jain M., Tyagi A.K., Khurana J.P. (2008). Genomic survey and gene expression analysis of the basic leucine zipper transcription factor family in rice. Plant Physiol..

[13] Wei K., Chen J., Wang Y., Chen Y., Chen S., Lin Y., Pan S., Zhong X., Xie D. (2012). Genome-wide analysis of bZIP-encoding genes in maize. DNA Res..

[14] Dai S., Petruccelli S., Ordiz M.I., Zhang Z., Chen S., Beachy R.N. (2003). Functional analysis of RF2a, a rice transcription factor. J. Biol. Chem..

[15] Dai S., Zhang Z., Chen S., Beachy R.N. (2004). RF2b, a rice bZIP transcription activator, interacts with RF2a and is involved in symptom development of rice tungro disease. Proc. Natl. Acad. Sci. USA.

[16] Guo G., Yang Y., Cao L., Liu W., Bi C. (2019). RF2 basic leucine zipper transcription factor TabZIP3 involved in salt stress response in wheat. J. Agric. Sci. Technol..

[17] Peng R., Xu Y., Tian S., Unver T., Liu Z., Zhou Z., Cai X., Wang K., Wei Y., Liu Y. (2022). Evolutionary divergence of duplicated genomes in newlydescribed allotetraploid cottons. Proc. Natl. Acad. Sci. USA.

[18] Peng R., Jones D.C., Liu F., Zhang B.H. (2021). From sequencing to genome editing for cotton improvement. Trend Biotech.

[19] Zhao Z., Shuang J., Li Z., Xiao H., Liu Y., Wang T., Wei Y., Hu S., Wan S., Peng R. (2021). Identification of the Golden-2-like transcription factors gene family in *Gossypium hirsutum*. PeerJ.

[20] Prakash S., Kumar M., Radha, Kumar S., Jaconis S., Parameswari E., Sharma K., Dhumal S., Senapathy M., Deshmukh V.P. (2023). The resilient cotton plant: Uncovering the effects of stresses on secondary metabolomics and its underlying molecular mechanisms. Funct. Integr. Genom..

[21] Pourabed E., Ghane Golmohamadi F., Soleymani Monfared P., Razavi S.M., Shobbar Z.S. (2015). Basic leucine zipper family in barley: Genome-wide characterization of members and expression analysis. Mol. Biotechnol..

[22] Zhang Y., Wang J., Chen X., Lu X., Wang D., Wang J., Wang S., Chen C., Guo L., Malik W.A. (2021). Genome-wide identification and characteristic analysis of the downstream melatonin metabolism gene GhM2H in *Gossypium hirsutum* L.. Biol. Res..

[23] Wang M., Tu L., Yuan D., Zhu D., Shen C., Li J., Liu F., Pei L., Wang P., Zhao G. (2019). Reference genome sequences of two cultivated allotetraploid cottons, *Gossypium hirsutum* and Gossypium barbadense. Nat. Genet..

[24] Du X., Huang G., He S., Yang Z., Sun G., Ma X., Li N., Zhang X., Sun J., Liu M. (2018). Resequencing of 243 diploid cotton accessions based on an updated A genome identifies the genetic basis of key agronomic traits. Nat. Genet..

[25] Udall J.A., Long E., Hanson C., Yuan D., Ramaraj T., Conover J.L., Gong L., Arick M.A., Grover C.E., Peterson D.G. (2019). De Novo Genome Sequence Assemblies of Gossypium raimondii and Gossypium turneri. G3.

[26] Yang Z., Wang J., Huang Y., Wang S., Wei L., Liu D., Weng Y., Xiang J., Zhu Q., Yang Z. (2023). CottonMD: A multi-omics database for cotton biological study. Nucleic Acids Res..

[27] Chen C., Chen H., Zhang Y., Thomas H.R., Frank M.H., He Y., Xia R. (2020). TBtools: An Integrative Toolkit Developed for Interactive Analyses of Big Biological Data. Mol. Plant.

[28] Mistry J., Chuguransky S., Williams L., Qureshi M., Salazar G.A., Sonnhammer E.L.L., Tosatto S.C.E., Paladin L., Raj S., Richardson L.J. (2021). Pfam: The protein families database in 2021. Nucleic Acids Res..

[29] Hao X.D., Liu Y., Li B.W., Wu W., Zhao X.W. (2020). Exome sequencing analysis identifies novel homozygous mutation in ABCA4 in a Chinese family with Stargardt disease. Int. J. Ophthalmol..

[30] Horton P., Park K.J., Obayashi T., Fujita N., Harada H., Adams-Collier C.J., Nakai K. (2007). WoLF PSORT: Protein localization predictor. Nucleic Acids Res..

[31] Hall B.G. (2013). Building phylogenetic trees from molecular data with MEGA. Mol. Biol. Evol..

[32] He Z., Zhang H., Gao S., Lercher M.J., Chen W.H., Hu S. (2016). Evolview v2: An online visualization and management tool for customized and annotated phylogenetic trees. Nucleic Acids Res..

[33] Bailey T.L., Boden M., Buske F.A., Frith M., Grant C.E., Clementi L., Ren J., Li W.W., Noble W.S. (2009). MEME SUITE: Tools for motif discovery and searching. Nucleic Acids Res..

[34] Lescot M., Déhais P., Thijs G., Marchal K., Moreau Y., Van de Peer Y., Rouzé P., Rombauts S. (2002). PlantCARE, a database of plant cis-acting regulatory elements and a portal to tools for in silico analysis of promoter sequences. Nucleic Acids Res..

[35] Bolger A.M., Lohse M., Usadel B. (2014). Trimmomatic: A flexible trimmer for Illumina sequence data. Bioinformatics.

[36] Kim D., Langmead B., Salzberg S.L. (2015). HISAT: A fast spliced aligner with low memory requirements. Nat. Methods.

[37] Ghosh S., Chan C.K. (2016). Analysis of RNA-Seq Data Using TopHat and Cufflinks. Methods Mol. Biol..

[38] Xie F., Wang J., Zhang B.H. (2023). RefFinder: A web-based tool for comprehensively analyzing and identifying reference genes. Funct. Integr. Genom..

[39] Flagel L.E., Wendel J.F., Udall J.A. (2012). Duplicate gene evolution, homoeologous recombination, and transcriptome characterization in allopolyploid cotton. BMC Genom..

[40] Senchina D.S., Alvarez I., Cronn R.C., Liu B., Rong J., Noyes R.D., Paterson A.H., Wing R.A., Wilkins T.A., Wendel J.F. (2003). Rate variation among nuclear genes and the age of polyploidy in Gossypium. Mol. Biol. Evol..

[41] Jakoby M., Weisshaar B., Dröge-Laser W., Vicente-Carbajosa J., Tiedemann J., Kroj T., Parcy F., bZIP Research Group (2002). bZIP transcription factors in Arabidopsis. Trends Plant Sci..

[42] Xue C., Qiu S.R., Li H., Li S.J., Li X., Xiang H., Zeng S.H., Liu Y.J. (2020). Identification of bZIP gene family and gene expression analysis of its subgroup A under ABA treatment in Nicotiana tabacum. Fenzi Zhiwu Yuzhong (Mol. Plant Breed.).

[43] Hajibarat Z., Saidi A., Hajibarat Z. (2022). Genome-wide identification of 14-3-3 gene family and characterization of their expression in developmental stages of Solanum tuberosum under multiple biotic and abiotic stress conditions. Funct. Integr. Genom..

[44] Lu X., Zhang H., Hu J., Nie G., Khan I., Feng G., Zhang X., Wang X., Huang L. (2022). Genome-wide identification and characterization of bHLH family genes from orchardgrass and the functional characterization of DgbHLH46 and DgbHLH128 in drought and salt tolerance. Funct. Integr. Genom..

[45] Xing L., Peng K., Xue S., Yuan W., Zhu B., Zhao P., Wu H., Cheng Y., Fang M., Liu Z. (2022). Genome-wide analysis of zinc finger-homeodomain (ZF-HD) transcription factors in diploid and tetraploid cotton. Funct. Integr. Genom..

[46] Duan P., Wei M., Zhang R., Zhao S., Wang Y., Gou B., Yang N., Zhang T., Zhang G., Wei B. (2022). Identification and bioinformatic analysis of the CaCesA/Csls family members and the expression of the CaCslD1 in the flower buds of CMS/Rf system in pepper. Funct. Integr. Genom..

[47] Mallick B., Kumari M., Pradhan S.K., Acharya G.C., Naresh P., Das B., Shashankar P. (2022). Genome-wide analysis and characterization of heat shock transcription factors (Hsfs) in common bean (*Phaseolus vulgaris* L.). Funct. Integr. Genom..

[48] Cheng Y.-Z., He G.-Q., Yang S.-D., Ma S.-H., Ma J.-P., Shang F.-H.-Z., Li X.-F., Jin H.-Y., Guo D.-L. (2022). Genome-wide identification and expression analysis of JmjC domain–containing genes in grape under MTA treatment. Funct. Integr. Genom..

[49] Huang G., Wu Z., Percy R.G., Bai M., Li Y., Frelichowski J.E., Hu J., Wang K., Yu J.Z., Zhu Y. (2020). Genome sequence of Gossypium herbaceum and genome updates of Gossypium arboreum and *Gossypium hirsutum* provide insights into cotton A-genome evolution. Nat. Genet..

[50] Liu C., Mao B., Ou S., Wang W., Liu L., Wu Y., Chu C., Wang X. (2014). OsbZIP71, a bZIP transcription factor, confers salinity and drought tolerance in rice. Plant Mol. Biol..

[51] Luang S., Sornaraj P., Bazanova N., Jia W., Eini O., Hussain S.S., Kovalchuk N., Agarwal P.K., Hrmova M., Lopato S. (2018). The wheat TabZIP2 transcription factor is activated by the nutrient starvation-responsive SnRK3/CIPK protein kinase. Plant Mol. Biol..

[52] Ying S., Zhang D.F., Fu J., Shi Y.S., Song Y.C., Wang T.Y., Li Y. (2012). Cloning and characterization of a maize bZIP transcription factor, ZmbZIP72, confers drought and salt tolerance in transgenic Arabidopsis. Planta.

[53] Cai W., Yang Y., Wang W., Guo G., Liu W., Bi C. (2018). Overexpression of a wheat (*Triticum aestivum* L.) bZIP transcription factor gene, TabZIP6, decreased the freezing tolerance of transgenic Arabidopsis seedlings by down-regulating the expression of CBFs. Plant Physiol. Biochem..

[54] Wang L., Cao H., Qian W., Yao L., Hao X., Li N., Yang Y., Wang X. (2017). Identification of a novel bZIP transcription factor in Camellia sinensis as a negative regulator of freezing tolerance in transgenic arabidopsis. Ann. Bot..

[55] Liu W., Zhang B.H. (2022). The landscape of genome sequencing and assembling in plants. Funct. Integr. Genom..

[56] Li C., Brant E., Budak H., Zhang B.H. (2021). CRISPR/Cas: A Nobel Prize award-winning precise genome editing technology for gene therapy and crop improvement. J. Zhejiang Univ. Sci. B.

[57] Li C., Chu W., Gill R.A., Sang S., Shi Y., Hu X., Yang Y., Zaman Q.U., Zhang B.H. Computational tools and resources for CRISPR/Cas genome editing. Genom. Proteom. Bioinform..

