# Supplementary material for: Genome-Wide Identification and Functional Analysis of RF2 Gene Family and the Critical Role of GhRF2-32 in Response to Drought Stress in Cotton
Source: Plants (Basel). 2023 Jul 11;12(14):2613. doi: 10.3390/plants12142613 (PMC10385120; doi:10.3390/plants12142613)
Supplement: Supplementary file 1 [file plants-12-02613-s001.zip › plants-2464776-supplementary.pdf]

Table S1: Use NCBI designed differentially expressed gene-specific primer sequences.

Primer name	sequence (5' to 3')	Number of bases	Purification method
GhActin QF	ATCCTCCGTCTTGACCTTG	19	PAGE
GhActin QR	TGTCCGTCAGGCAACTCAT	19	PAGE
GhRF2-2F	GGAGCTAATCAACAATACTACCC CA	25	PAGE
GhRF2-2R	CCCAGTTTGATGTTGCGGTT	20	PAGE
GhRF2-3F	CCTCCGATGACTCCTCATGC	20	PAGE
GhRF2-3R	CAACCCGAGTCCATCAAAGA	20	PAGE
GhRF2-4F	GCAACAGGCCCAACTGAAAG	20	PAGE
GhRF2-4R	CCTCTCCACTGAACTCTGCG	20	PAGE
GhRF2-5F	TTTCCCACTGTTTCCCCAGC	20	PAGE
GhRF2-5R	GGAGGCTTTCGGAGGAAGTG	20	PAGE
GhRF2-6F	CTCGGACCGACTCATTCTCG	20	PAGE
GhRF2-6R	GGCAAACTTTAACGAATGCGA	21	PAGE
GhRF2-16F	TCCAAGTGGGCTTCCTTCTTTA	22	PAGE
GhRF2-16R	CATTTGCTGTTGATTGTGCTCG	22	PAGE
GhRF2-20F	CATACTCTGCTAACCGCGCA	20	PAGE
GhRF2-20R	CCATGTCCCCCGTTTGATGT	20	PAGE
GhRF2-22F	AAGTCCAGCGACTGAAGCTC	20	PAGE
GhRF2-22R	ATGTTTCGCTGCTGCTGTTG	20	PAGE
GhRF2-23F	TGCACTTCCTCTGAAAGCCT	20	PAGE
GhRF2-23R	GCACAGGAGTCTCTGGTTCA	20	PAGE
GhRF2-24F	CCGCGGAAGTCCATCGATTA	20	PAGE
GhRF2-24R	CTGTTGCTGGTGTAGCTGGA	20	PAGE
GhRF2-32F	ATCCACCAAGTCTGAAGCCG	20	PAGE
GhRF2-32R	AGAGCCTCGGAAAGTGGTTG	20	PAGE
GhRF2-35F	CTTTAACGGCGGTGCTGCT	19	PAGE
GhRF2-35R	ATCTGGTTGGGGTGTGAAGG	20	PAGE
RF2-32V-XbaI-F	AAGGTTACCGAATTCTCTAGACA CCCCTTCTTTTCCCAACC	41	PAGE
RF2-32V-KpnI-R	GAGACGCGTGAGCTCGGTACCCC TTCATTAGTCTTCTCCCCCA	43	PAGE
RF2b-DsRED2-F	ctaggtgtgttttgcaagcttATGCAGGATCC ACCAAGTCTGA	43	PAGE
RF2b-DsRED2-R	acgacggccagtgccaagcttTCATAATGTT CCACTACTTTCACCAG	47	PAGE

Table S2. Physico-chemical and biochemical characteristics of RF2 genes in cotton

Transcript ID	gene name	Length (aa)	ORF length(bp)	MW	PI	Stability index	Subcellular prediction
evm.model.Ga01G0609	GaRF2-1	320	963	35999.05	6.51	62.38	Nuclear
evm.model.Ga03G0132	GaRF2-2	386	1161	41901.59	5.83	47.83	Nuclear
evm.model.Ga03G0756	GaRF2-3	341	1026	37967.43	7.87	57.85	Nuclear
evm.model.Ga03G1126	GaRF2-4	570	1713	62392.85	6.19	68.16	Nuclear
evm.model.Ga03G2503	GaRF2-5	370	1113	41478.06	9.17	68.14	Nuclear
evm.model.Ga03G2578	GaRF2-6	552	1659	59952.97	6.35	60.19	Nuclear
evm.model.Ga04G0296	GaRF2-7	363	1092	40943.53	8.34	70.15	Nuclear
evm.model.Ga04G1998	GaRF2-8	341	1026	37612.05	5.69	52.81	Nuclear
evm.model.Ga05G2881	GaRF2-9	323	972	35523.38	6.74	48.88	Nuclear
evm.model.Ga05G2977	GaRF2-10	366	1101	39941.29	5.78	55.40	Nuclear
evm.model.Ga06G2190	GaRF2-11	369	1110	39858.34	5.68	45.14	Nuclear/chloroplast
evm.model.Ga08G2367	GaRF2-12	220	663	24923.90	5.80	38.66	Nuclear
evm.model.Ga09G2394	GaRF2-13	415	1248	44992.04	5.65	52.09	Nuclear
evm.model.Ga10G0927	GaRF2-14	361	1086	39327.88	5.83	51.63	Nuclear
evm.model.Ga10G1669	GaRF2-15	398	1197	43657.51	6.44	52.22	Nuclear
evm.model.Ga11G0563	GaRF2-16	284	855	31952.72	5.59	59.06	Nuclear
evm.model.Ga13G0339	GaRF2-17	381	1146	42583.94	8.36	63.35	Nuclear
evm.model.Ga13G1080	GaRF2-18	567	1704	61330.68	6.30	54.32	Nuclear
evm.model.Ga13G1858	GaRF2-19	319	960	35902.12	6.34	72.45	Nuclear
evm.model.Ga14G0186	GaRF2-20	366	1101	41362.63	6.65	69.57	Nuclear
Gbar_A01G004970.1	GbRF2-1	320	963	35999.05	6.51	62.38	Nuclear
Gbar_A02G001100.1	GbRF2-2	386	1161	41899.57	5.83	48.61	Nuclear
Gbar_A02G006540.1	GbRF2-3	337	1014	37499.96	7.89	54.64	Nuclear
Gbar_A03G009220.1	GbRF2-4	572	1719	62567.05	6.30	67.75	Nuclear
Gbar_A03G021150.1	GbRF2-5	370	1113	41479.00	8.91	68.37	Nuclear
Gbar_A03G021900.1	GbRF2-6	552	1659	59961.04	6.49	61.54	Nuclear
Gbar_A05G026560.1	GbRF2-7	323	972	35667.55	6.96	49.59	Nuclear
Gbar_A05G027450.1	GbRF2-8	371	1116	40489.95	5.85	55.69	Nuclear
Gbar_A05G040630.1	GbRF2-9	341	1026	37486.83	5.57	52.25	Nuclear
Gbar_A06G019370.1	GbRF2-10	248	747	27199.55	7.79	39.97	Nuclear/chloroplast
Gbar_A07G025540.1	GbRF2-11	319	960	35872.03	6.34	72.57	Nuclear
Gbar_A09G022230.1	GbRF2-12	413	1242	44920.97	5.96	52.62	Nuclear
Gbar_A10G014980.1	GbRF2-13	398	1197	43658.49	6.41	50.16	Nuclear
Gbar_A10G019270.1	GbRF2-14	361	1086	39475.04	5.83	50.16	Nuclear
Gbar_A11G030850.1	GbRF2-15	283	852	31838.62	5.59	60.10	Nuclear
Gbar_A12G002500.1	GbRF2-16	367	1104	41331.52	6.65	68.30	Nuclear
Gbar_A13G003620.1	GbRF2-17	381	1146	42536.84	7.20	64.00	Nuclear

Gbar_A13G009350.1	GbRF2-18	567	1704	61353.72	6.35	54.32	Nuclear
Gbar_D01G005120.1	GbRF2-19	320	963	36067.13	6.65	61.91	Nuclear
Gbar_D02G001400.1	GbRF2-20	386	1161	41920.67	6.01	44.75	Nuclear
Gbar_D02G007490.1	GbRF2-21	334	1005	37189.64	8.51	53.15	Nuclear
Gbar_D02G010420.1	GbRF2-22	563	1692	61959.48	6.59	65.96	Nuclear
Gbar_D02G023090.1	GbRF2-23	370	1113	41357.68	7.75	64.06	Nuclear
Gbar_D02G023830.1	GbRF2-24	556	1671	60289.43	6.42	61.27	Nuclear
Gbar_D04G001290.1	GbRF2-25	341	1026	37545.99	5.69	52.89	Nuclear
Gbar_D04G017730.1	GbRF2-26	350	1053	39175.24	6.28	65.45	Nuclear
Gbar_D04G017740.1	GbRF2-27	382	1149	42921.72	7.18	68.92	Nuclear/chloroplast
Gbar_D05G027400.1	GbRF2-28	323	972	35527.43	6.96	48.17	Nuclear
Gbar_D05G028280.1	GbRF2-29	371	1116	40410.85	6.04	57.12	Nuclear
Gbar_D06G020250.1	GbRF2-30	264	795	29212.01	9.13	44.99	Nuclear/chloroplast
Gbar_D09G021980.1	GbRF2-31	413	1242	44784.84	5.90	52.24	Nuclear
Gbar_D10G013120.1	GbRF2-32	398	1197	43664.44	6.53	51.51	Nuclear
Gbar_D10G019420.1	GbRF2-33	361	1086	39209.70	5.66	51.84	Nuclear
Gbar_D11G031450.1	GbRF2-34	277	834	31285.12	5.52	61.24	Nuclear
Gbar_D12G002730.1	GbRF2-35	372	1119	41887.15	6.85	65.61	Nuclear
Gbar_D12G023100.1	GbRF2-36	113	342	13220.02	6.91	35.50	Nuclear
Gbar_D13G003400.1	GbRF2-37	379	1140	42413.72	7.80	62.60	Nuclear
Gbar_D13G009810.1	GbRF2-38	567	1704	61376.67	6.17	53.49	Nuclear
Gbar_D13G016200.1	GbRF2-39	260	783	28995.66	6.23	64.89	Nuclear
Ghir_A01G022860.1	GhRF2-1	320	963	35999.05	6.51	62.38	Nuclear
Ghir_A02G001350.1	GhRF2-2	386	1161	41899.57	5.83	48.61	Nuclear
Ghir_A02G006740.1	GhRF2-3	340	1023	37884.35	7.89	55.95	Nuclear
Ghir_A03G009200.1	GhRF2-4	570	1713	62360.85	6.30	67.02	Nuclear
Ghir_A03G021100.1	GhRF2-5	370	1113	41479.00	8.91	68.37	Nuclear
Ghir_A03G021810.1	GhRF2-6	536	1611	58213.97	6.22	57.88	Nuclear
Ghir_A05G027550.1	GhRF2-7	364	1095	40054.03	8.68	46.02	Nuclear/chloroplast
Ghir_A05G028410.1	GhRF2-8	330	993	35979.36	7.08	43.87	Nuclear
Ghir_A05G041580.1	GhRF2-9	341	1026	37545.90	5.69	52.33	Nuclear
Ghir_A06G019360.1	GhRF2-10	354	1065	38204.46	5.51	41.49	Nuclear/chloroplast
Ghir_A09G022110.1	GhRF2-11	413	1242	44920.97	5.96	52.62	Nuclear
Ghir_A10G014150.1	GhRF2-12	398	1197	43672.52	6.53	50.62	Nuclear
Ghir_A10G018390.1	GhRF2-13	359	1080	38972.35	5.59	48.49	Nuclear
Ghir_A11G031340.1	GhRF2-14	284	855	31968.76	5.59	58.38	Nuclear
Ghir_A12G002680.1	GhRF2-15	387	1164	43898.61	8.91	64.20	Nuclear
Ghir_A13G003420.1	GhRF2-16	381	1146	42583.94	8.36	63.35	Nuclear
Ghir_A13G009010.1	GhRF2-17	553	1662	59905.16	6.22	53.30	Nuclear
Ghir_A13G015530.1	GhRF2-18	319	960	35872.03	6.34	72.57	Nuclear

Ghir_D01G005170.1	GhRF2-19	320	963	36093.21	6.56	61.13	Nuclear
Ghir_D02G001430.1	GhRF2-20	386	1161	41917.71	6.01	45.88	Nuclear
Ghir_D02G007160.1	GhRF2-21	228	687	25027.22	6.71	54.15	Nuclear
Ghir_D02G010000.1	GhRF2-22	563	1692	61911.48	6.75	65.30	Nuclear
Ghir_D02G022550.1	GhRF2-23	370	1113	41357.68	7.75	65.62	Nuclear
Ghir_D02G023300.1	GhRF2-24	556	1671	60275.36	6.30	60.57	Nuclear
Ghir_D04G001370.1	GhRF2-25	341	1026	37531.96	5.69	52.97	Nuclear
Ghir_D04G017590.1	GhRF2-26	335	1008	37379.10	6.46	68.54	Nuclear
Ghir_D05G027570.1	GhRF2-27	323	972	35485.39	7.31	49.52	Nuclear
Ghir_D05G028420.1	GhRF2-28	371	1116	40357.80	5.89	58.00	Nuclear
Ghir_D06G020520.1	GhRF2-29	377	1134	41036.62	5.81	48.38	Nuclear/chloroplast
Ghir_D09G021400.1	GhRF2-30	413	1242	44842.88	5.79	52.24	Nuclear
Ghir_D10G013410.1	GhRF2-31	379	1140	41651.17	6.66	53.30	Nuclear
Ghir_D10G019890.1	GhRF2-32	361	1086	39214.72	5.59	52.15	Nuclear
Ghir_D12G002680.1	GhRF2-33	369	1110	41565.81	6.85	65.02	Nuclear
Ghir_D13G003680.1	GhRF2-34	379	1140	42413.72	7.80	62.60	Nuclear
Ghir_D13G009990.1	GhRF2-35	567	1704	61391.64	6.17	54.06	Nuclear
Ghir_D13G016290.1	GhRF2-36	290	873	32672.44	6.61	73.98	Nuclear
XP_012441813.1	GrRF2-1	567	1704	61304.65	6.30	53.19	Nuclear
XP_012457700.1	GrRF2-2	320	963	35974.05	6.56	64.17	Nuclear
XP_012481534.1	GrRF2-3	386	1161	42018.77	5.90	48.36	Nuclear
XP_012478118.1	GrRF2-4	569	1710	62572.12	6.45	66.37	Nuclear
XP_012480294.1	GrRF2-5	370	1113	41429.73	8.40	66.79	Nuclear
XP_012480421.1	GrRF2-6	556	1671	60289.38	6.30	59.94	Nuclear
XP_012487039.1	GrRF2-7	413	1242	44828.85	5.79	52.24	Nuclear
XP_012488308.1	GrRF2-8	280	843	31510.27	5.38	60.19	Nuclear
XP_012436227.1	GrRF2-9	374	1125	42111.36	6.85	66.43	Nuclear
XP_012446799.1	GrRF2-10	323	972	35543.43	6.96	48.93	Nuclear
XP_012447231.1	GrRF2-11	371	1116	40353.75	6.04	57.32	Nuclear
XP_012449361.1	GrRF2-12	349	1050	37764.96	5.64	45.41	Nuclear/chloroplast
XP_012456530.1	GrRF2-13	398	1197	43657.41	6.38	49.57	Nuclear
XP_012456653.1	GrRF2-14	361	1086	39228.74	5.59	53.06	Nuclear
XP_012460397.1	GrRF2-15	341	1026	37432.76	5.57	53.06	Nuclear
XP_012460807.1	GrRF2-16	363	1092	40677.87	6.61	64.77	Nuclear
XP_012463962.1	GrRF2-17	379	1140	42370.74	8.64	63.75	Nuclear
XP_012464256.1	GrRF2-18	319	960	35871.10	6.34	72.89	Nuclear

Table S3. FPKM value of each gene presented in Figure 6

		4°C 1h	4°C 3h	4°C 6h	4°C 12h	4°C 24h	NaCl 1h	NaCl 3h	NaCl 6h	NaCl 12h	NaCl 24h	PEG 1h	PEG 3h	PEG 6h	PEG 12h	PEG 24h
Ghir_A01G022860.1	GhRF2-1	1.125359	0.421888	3.032503	0.234322	0.619973	0.121385	0.202156	0.32055	0.428481	0.325875	0.191437	0.169832	0.181163	0.89282	0.266219
Ghir_A02G001350.1	GhRF2-2	3.142973	4.178137	4.178843	5.563423	6.033443	3.208757	5.783	5.796437	8.0806	3.29283	4.622527	5.76264	5.801367	6.91742	2.971487
Ghir_A02G006740.1	GhRF2-3	5.11465	5.777067	3.847627	3.914937	3.588397	5.965667	6.848587	5.140423	6.423375	6.456917	6.904253	8.487477	7.039667	8.562827	6.64241
Ghir_A03G009200.1	GhRF2-4	5.16443	5.422053	5.82138	4.296883	4.769027	4.7229	5.81069	6.725787	7.45368	4.287233	5.5626	6.385027	6.023357	9.20926	5.110577
Ghir_A03G021100.1	GhRF2-5	2.706437	3.26012	4.067003	3.930347	3.778277	5.227793	4.732277	3.508667	8.243405	3.323077	2.931253	3.635423	4.199383	7.48287	3.80887
Ghir_A03G021810.1	GhRF2-6	9.167237	15.0691	11.70023	25.931	17.85043	6.17765	14.1562	11.49777	14.934	8.801467	8.611653	9.79303	10.40757	15.9889	8.264723
Ghir_A05G027550.1	GhRF2-7	1.271605	2.37915	1.69784	2.148609	3.2494	1.886324	2.139364	1.834639	3.52884	1.509283	2.811557	3.114083	2.54952	2.365473	0.979006
Ghir_A05G028410.1	GhRF2-8	0.169941	0.208619	0	0.120706	0	0	0.018533	0.037242	0.299656	0	0.020125	0.034914	0.092542	0.133915	0.041109
Ghir_A05G041580.1	GhRF2-9	1.138307	0.593992	1.045159	0.258995	0.207488	0.408	0.423204	0.399232	1.153976	0.531702	0.562698	0.301235	0.556149	1.77011	0.90178
Ghir_A06G019360.1	GhRF2-10	1.24473	1.191722	1.273753	0.850207	0.800988	0.866906	0.697133	0.781655	0.626101	0.671104	0.505932	0.387928	0.528108	0.963711	0.89248
Ghir_A09G022110.1	GhRF2-11	1.857317	2.501917	1.910067	1.163825	1.052953	0.818728	1.55001	1.9449	1.300866	1.034966	1.517363	1.13674	1.596626	2.21399	1.123849
Ghir_A10G014150.1	GhRF2-12	0.931602	1.011521	1.519542	1.946068	1.779656	3.679953	1.49701	1.429143	2.27523	0.742379	1.222603	1.278599	1.45907	2.265687	1.035543
Ghir_A10G018390.1	GhRF2-13	1.268132	0.933428	1.008776	0.65891	0.34891	1.4777	0.749255	0.883508	1.515995	0.35789	0.793918	0.565478	0.835507	1.728507	0.613575
Ghir_A11G031340.1	GhRF2-14	0.039834	0	0	0	0	0.085187	0	0	0	0	0	0	0	0	0
Ghir_A12G002680.1	GhRF2-15	0.535921	0.535732	0.744996	0.348004	0.154311	0.149382	0.381139	0.547196	0.598702	0.218396	0.220726	0.275611	0.21234	1.598848	0.188793
Ghir_A13G003420.1	GhRF2-16	3.870483	8.741437	13.33746	6.816743	3.492853	5.32787	5.65303	7.009013	6.274465	2.037687	5.602397	5.26954	5.13008	4.33877	2.597563
Ghir_A13G009010.1	GhRF2-17	7.914783	12.71081	7.30222	8.768907	6.549463	5.51394	6.772367	6.57741	6.21	6.379927	6.16629	5.578107	4.642873	8.3372	6.99526
Ghir_A13G015530.1	GhRF2-18	0.149494	0.129193	0.413265	0.071001	0.05765	0.043466	0.040462	0.068221	0.032412	0.006201	0.014523	0.121977	0.057978	0.106537	0.11685
Ghir_D01G005170.1	GhRF2-19	1.273937	0.751422	4.363224	0.733575	1.468435	0.316759	0.608793	0.66458	0.5833	0.391861	0.38133	0.711966	0.720383	1.256948	0.395534
Ghir_D02G001430.1	GhRF2-20	5.306097	7.80598	7.25784	8.794553	7.601833	3.579503	7.266197	6.888283	10.69907	4.28281	6.798927	8.10332	7.168647	10.43908	5.544873
Ghir_D02G007160.1	GhRF2-21	5.116507	5.40005	2.91979	3.676337	3.47741	4.716087	5.570583	5.102127	7.36056	4.13498	7.59803	6.26456	5.74385	6.79431	4.197593
Ghir_D02G010000.1	GhRF2-22	6.220553	6.275287	6.838503	6.088037	6.376857	5.964997	6.47097	5.88501	9.04754	5.13287	5.519663	6.787913	6.4795	9.317453	5.54536
Ghir_D02G022550.1	GhRF2-23	1.920263	2.617127	2.612123	2.67636	3.78214	6.29191	3.40534	3.305683	6.77737	2.485263	2.905003	2.726067	2.73711	6.19452	3.14622
Ghir_D02G023300.1	GhRF2-24	7.391207	19.50067	21.5744	41.6283	30.9102	4.565553	15.4241	16.49453	20.8609	4.83017	8.865093	10.18639	14.14297	25.0625	6.517647
Ghir_D04G001370.1	GhRF2-25	1.044226	1.249953	1.04185	1.088689	0.641272	0.606554	0.819614	0.885307	1.23921	0.835779	0.6075	0.797117	0.770968	0.906211	0.784064
Ghir_D04G017590.1	GhRF2-26	2.231021	1.78481	0.781745	1.009815	0.487167	0.204251	0.734138	1.151526	1.441004	1.026545	1.144538	1.463365	1.058778	0.772547	1.378406
Ghir_D05G027570.1	GhRF2-27	3.593467	6.244987	3.88937	6.781143	9.237257	5.826573	7.10295	4.955647	10.28886	7.525407	8.135547	8.463157	8.820693	6.34814	5.30038
Ghir_D05G028420.1	GhRF2-28	1.096928	0.494797	0.415027	0.139416	0.056765	0.338755	0.467909	0.436246	1.168555	0.203687	0.403204	0.361538	0.339321	1.269009	0.445098
Ghir_D06G020520.1	GhRF2-29	1.966155	1.386173	1.252359	0.986209	0.920135	2.560233	1.5388	1.40901	1.64184	1.041589	1.194245	1.310103	1.157085	2.001923	1.969013
Ghir_D09G021400.1	GhRF2-30	2.039134	1.46643	1.78535	1.663827	1.77072	1.003431	2.277097	1.88051	1.803999	1.048191	2.096917	2.394572	2.413757	2.262747	1.658632
Ghir_D10G013410.1	GhRF2-31	1.987923	2.9719	2.300542	2.758087	3.461613	3.046633	2.131431	2.115687	4.485215	1.211249	1.706021	1.81253	3.04662	2.48625	1.227398
Ghir_D10G019890.1	GhRF2-32	4.345757	2.437463	4.37561	2.182223	1.984967	2.868594	2.62403	2.57811	4.215235	1.42086	1.778913	1.977092	2.03157	4.998657	2.259597
Ghir_D12G002680.1	GhRF2-33	0.858036	0.988781	1.094811	0.640697	0.46111	0.672858	0.904027	1.170702	1.48601	0.6832	0.930867	1.160736	1.16113	1.819657	1.173599
Ghir_D13G003680.1	GhRF2-34	1.628151	2.499873	5.073103	1.741543	0.975605	6.763027	2.10453	1.726957	1.936855	0.830573	1.747123	1.79186	2.134	1.973027	0.96439
Ghir_D13G009990.1	GhRF2-35	6.759963	10.65141	8.224853	7.560457	5.74037	6.306477	5.05891	4.644453	5.657275	5.073193	5.043577	3.849313	4.082903	8.298593	6.704423
Ghir_D13G016290.1	GhRF2-36	0.322297	0.32178	0.268806	0.024313	0.039497	0	0.245406	0.113224	0.036273	0	0	0.022528	0.042917	0.090537	0.069679

Table S4a. cis elements in Ga RF2 genes

evm.model.Ga01G0609	1600	6	-	light responsiveness
evm.model.Ga01G0609	1116	7	-	auxin responsiveness
evm.model.Ga01G0609	1600	6	-	abscisic acid responsiveness
evm.model.Ga01G0609	1601	5	+	abscisic acid responsiveness
evm.model.Ga01G0609	308	6	+	light responsiveness
evm.model.Ga01G0609	510	6	+	light responsiveness
evm.model.Ga01G0609	580	6	+	light responsiveness
evm.model.Ga01G0609	601	6	+	light responsiveness
evm.model.Ga01G0609	621	6	+	light responsiveness
evm.model.Ga01G0609	763	6	+	light responsiveness
evm.model.Ga01G0609	323	6	+	anaerobic induction
evm.model.Ga01G0609	1032	6	-	anaerobic induction
evm.model.Ga01G0609	1000	7	+	light responsiveness
evm.model.Ga01G0609	1777	6	-	light responsiveness
evm.model.Ga01G0609	1600	6	-	light responsiveness
evm.model.Ga01G0609	1868	6	-	light responsiveness
evm.model.Ga03G0132	829	6	+	low-temperature responsiveness
evm.model.Ga03G0132	261	6	+	light responsiveness
evm.model.Ga03G0132	999	5	-	MeJA responsiveness
evm.model.Ga03G0132	1725	9	+	salicylic acid responsiveness
evm.model.Ga03G0132	261	8	-	light responsiveness
evm.model.Ga03G0132	877	6	-	auxin responsiveness
evm.model.Ga03G0132	353	6	-	light responsiveness
evm.model.Ga03G0132	788	6	+	light responsiveness
evm.model.Ga03G0132	999	5	+	MeJA responsiveness
evm.model.Ga03G0132	497	10	+	light responsiveness
evm.model.Ga03G0132	340	6	+	light responsiveness
evm.model.Ga03G0132	926	6	+	light responsiveness
evm.model.Ga03G0132	946	6	+	light responsiveness
evm.model.Ga03G0132	1247	6	-	light responsiveness
evm.model.Ga03G0132	1337	6	-	light responsiveness
evm.model.Ga03G0132	1495	6	-	light responsiveness
evm.model.Ga03G0132	1605	6	-	light responsiveness
evm.model.Ga03G0132	261	5	-	abscisic acid responsiveness
evm.model.Ga03G0756	858	5	-	MeJA responsiveness
evm.model.Ga03G0756	136	10	+	light responsiveness
evm.model.Ga03G0756	275	10	-	light responsiveness
evm.model.Ga03G0756	346	6	-	light responsiveness
evm.model.Ga03G0756	1462	6	-	light responsiveness

evm.model.Ga03G0756	295	7	-	light responsiveness
evm.model.Ga03G0756	1959	10	+	light responsiveness
evm.model.Ga03G0756	782	6	-	drought inducibility
evm.model.Ga03G0756	333	9	-	defense and stress responsiveness
evm.model.Ga03G0756	1937	9	+	defense and stress responsiveness
evm.model.Ga03G0756	1702	6	-	light responsiveness
evm.model.Ga03G0756	183	5	+	abscisic acid responsiveness
evm.model.Ga03G0756	347	5	+	abscisic acid responsiveness
evm.model.Ga03G0756	860	5	+	abscisic acid responsiveness
evm.model.Ga03G0756	1463	5	+	abscisic acid responsiveness
evm.model.Ga03G0756	505	6	+	light responsiveness
evm.model.Ga03G0756	858	5	+	MeJA responsiveness
evm.model.Ga03G0756	1445	6	+	light responsiveness
evm.model.Ga03G0756	1545	6	-	light responsiveness
evm.model.Ga03G0756	289	6	-	auxin responsiveness
evm.model.Ga03G0756	182	6	+	light responsiveness
evm.model.Ga03G0756	859	6	-	light responsiveness
evm.model.Ga03G0756	1460	8	+	light responsiveness
evm.model.Ga03G1126	1235	10	+	light responsiveness
evm.model.Ga03G1126	265	6	-	light responsiveness
evm.model.Ga03G1126	1002	6	-	anaerobic induction
evm.model.Ga03G1126	1953	9.5	-	light responsiveness
evm.model.Ga03G1126	848	6	+	light responsiveness
evm.model.Ga03G1126	1697	6	-	light responsiveness
evm.model.Ga03G1126	1921	9	-	light responsiveness
evm.model.Ga03G1126	226	7	-	light responsiveness
evm.model.Ga03G1126	1055	6	-	auxin responsiveness
evm.model.Ga03G1126	241	6	-	light responsiveness
evm.model.Ga03G1126	163	6	+	light responsiveness
evm.model.Ga03G1126	771	6	-	light responsiveness
evm.model.Ga03G1126	1218	6	+	light responsiveness
evm.model.Ga03G1126	1896	6	-	light responsiveness
evm.model.Ga03G1126	241	5	-	abscisic acid responsiveness
evm.model.Ga03G1126	646	8	-	light responsiveness
evm.model.Ga03G1126	350	6	+	light responsiveness
evm.model.Ga03G1126	667	6	+	light responsiveness
evm.model.Ga03G1126	812	6	+	light responsiveness
evm.model.Ga03G1126	52	13	+	light responsiveness
evm.model.Ga03G1126	340	6	+	drought inducibility
evm.model.Ga03G1126	1982	6	-	drought inducibility

evm.model.Ga03G1126	1234	7	+	gibberellin responsiveness
evm.model.Ga03G2503	550	7	-	light responsiveness
evm.model.Ga03G2503	1671	7	-	light responsiveness
evm.model.Ga03G2503	1918	10	-	light responsiveness
evm.model.Ga03G2503	1128	6	+	low-temperature responsiveness
evm.model.Ga03G2503	17	9	+	light responsiveness
evm.model.Ga03G2503	1668	7	+	light responsiveness
evm.model.Ga03G2503	1892	9	-	light responsiveness
evm.model.Ga03G2503	415	6	+	light responsiveness
evm.model.Ga03G2503	793	6	+	light responsiveness
evm.model.Ga03G2503	1083	6	-	light responsiveness
evm.model.Ga03G2503	1944	6	-	light responsiveness
evm.model.Ga03G2503	846	10	-	light responsiveness
evm.model.Ga03G2503	588	9	-	salicylic acid responsiveness
evm.model.Ga03G2503	870	9	+	salicylic acid responsiveness
evm.model.Ga03G2503	1711	6	-	anaerobic induction
evm.model.Ga03G2503	1854	6	-	anaerobic induction
evm.model.Ga03G2503	852	6	+	light responsiveness
evm.model.Ga03G2503	57	6	-	auxin responsiveness
evm.model.Ga03G2578	356	6	+	drought inducibility
evm.model.Ga03G2578	121	5	+	MeJA responsiveness
evm.model.Ga03G2578	216	5	+	MeJA responsiveness
evm.model.Ga03G2578	1246	10	-	defense and stress responsiveness
evm.model.Ga03G2578	709	14	+	light responsiveness
evm.model.Ga03G2578	121	5	-	MeJA responsiveness
evm.model.Ga03G2578	216	5	-	MeJA responsiveness
evm.model.Ga03G2578	328	7	+	light responsiveness
evm.model.Ga03G2578	1963	6	+	light responsiveness
evm.model.Ga03G2578	1588	5	+	abscisic acid responsiveness
evm.model.Ga03G2578	700	6	+	light responsiveness
evm.model.Ga03G2578	1032	6	-	light responsiveness
evm.model.Ga03G2578	1218	6	-	light responsiveness
evm.model.Ga03G2578	905	8	-	light responsiveness
evm.model.Ga03G2578	1007	8	+	light responsiveness
evm.model.Ga03G2578	669	6	+	anaerobic induction
evm.model.Ga03G2578	1246	6	-	light responsiveness
evm.model.Ga03G2578	1727	8	-	light responsiveness
evm.model.Ga03G2578	1904	8	-	light responsiveness
evm.model.Ga03G2578	1587	6	+	light responsiveness
evm.model.Ga04G0296	205	9	-	salicylic acid responsiveness

evm.model.Ga04G0296	1020	6	-	light responsiveness
evm.model.Ga04G0296	171	6	-	light responsiveness
evm.model.Ga04G0296	880	6	+	light responsiveness
evm.model.Ga04G0296	1355	6	-	light responsiveness
evm.model.Ga04G0296	726	6	-	light responsiveness
evm.model.Ga04G0296	725	5	+	MeJA responsiveness
evm.model.Ga04G0296	1682	5	+	MeJA responsiveness
evm.model.Ga04G0296	229	9	-	light responsiveness
evm.model.Ga04G0296	727	5	+	abscisic acid responsiveness
evm.model.Ga04G0296	741	6	+	light responsiveness
evm.model.Ga04G0296	997	6	+	light responsiveness
evm.model.Ga04G0296	1612	8	-	light responsiveness
evm.model.Ga04G0296	110	6	+	light responsiveness
evm.model.Ga04G0296	260	6	+	light responsiveness
evm.model.Ga04G0296	747	6	+	light responsiveness
evm.model.Ga04G0296	904	6	+	light responsiveness
evm.model.Ga04G0296	911	6	+	light responsiveness
evm.model.Ga04G0296	609	8	+	light responsiveness
evm.model.Ga04G0296	1006	9	-	defense and stress responsiveness
evm.model.Ga04G0296	1355	9	-	defense and stress responsiveness
evm.model.Ga04G0296	725	5	-	MeJA responsiveness
evm.model.Ga04G0296	1682	5	-	MeJA responsiveness
evm.model.Ga04G1998	1275	6	+	light responsiveness
evm.model.Ga04G1998	1488	8	+	light responsiveness
evm.model.Ga04G1998	1686	8	+	light responsiveness
evm.model.Ga04G1998	1887	6	-	auxin responsiveness
evm.model.Ga04G1998	1193	6	+	anaerobic induction
evm.model.Ga04G1998	1592	6	-	anaerobic induction
evm.model.Ga04G1998	1553	8	-	light responsiveness
evm.model.Ga04G1998	564	6	+	light responsiveness
evm.model.Ga04G1998	1503	6	-	light responsiveness
evm.model.Ga04G1998	1087	6	-	light responsiveness
evm.model.Ga04G1998	877	7	-	abscisic acid responsiveness
evm.model.Ga04G1998	959	5	-	MeJA responsiveness
evm.model.Ga04G1998	959	5	+	MeJA responsiveness
evm.model.Ga05G2881	1781	5	+	MeJA responsiveness
evm.model.Ga05G2881	1957	5	+	MeJA responsiveness
evm.model.Ga05G2881	1873	7	+	gibberellin responsiveness
evm.model.Ga05G2881	1969	6	+	low-temperature responsiveness
evm.model.Ga05G2881	1765	13	-	light responsiveness

evm.model.Ga05G2881	101	6	+	light responsiveness
evm.model.Ga05G2881	1095	6	-	light responsiveness
evm.model.Ga05G2881	1119	6	-	light responsiveness
evm.model.Ga05G2881	1778	8	-	auxin responsiveness
evm.model.Ga05G2881	1848	5	+	abscisic acid responsiveness
evm.model.Ga05G2881	1781	5	-	MeJA responsiveness
evm.model.Ga05G2881	1957	5	-	MeJA responsiveness
evm.model.Ga05G2881	797	10	-	light responsiveness
evm.model.Ga05G2881	1847	6	+	light responsiveness
evm.model.Ga05G2881	1612	6	+	light responsiveness
evm.model.Ga05G2881	1911	6	+	light responsiveness
evm.model.Ga05G2881	419	6	+	anaerobic induction
evm.model.Ga05G2881	1536	10	+	light responsiveness
evm.model.Ga05G2977	211	6	+	light responsiveness
evm.model.Ga05G2977	881	6	+	light responsiveness
evm.model.Ga05G2977	1013	6	-	light responsiveness
evm.model.Ga05G2977	1110	6	-	light responsiveness
evm.model.Ga05G2977	1125	6	-	light responsiveness
evm.model.Ga05G2977	1179	6	-	light responsiveness
evm.model.Ga05G2977	387	8	+	light responsiveness
evm.model.Ga05G2977	50	6	+	abscisic acid responsiveness
evm.model.Ga05G2977	51	5	+	abscisic acid responsiveness
evm.model.Ga05G2977	842	5	-	abscisic acid responsiveness
evm.model.Ga05G2977	333	5	+	MeJA responsiveness
evm.model.Ga05G2977	844	5	-	MeJA responsiveness
evm.model.Ga05G2977	50	6	+	light responsiveness
evm.model.Ga05G2977	842	6	+	light responsiveness
evm.model.Ga05G2977	1412	9.5	-	light responsiveness
evm.model.Ga05G2977	295	6	-	anaerobic induction
evm.model.Ga05G2977	1513	6	-	anaerobic induction
evm.model.Ga05G2977	1764	6	+	anaerobic induction
evm.model.Ga05G2977	50	6	+	light responsiveness
evm.model.Ga05G2977	103	10	+	light responsiveness
evm.model.Ga05G2977	333	5	-	MeJA responsiveness
evm.model.Ga05G2977	844	5	+	MeJA responsiveness
evm.model.Ga05G2977	1899	6	+	low-temperature responsiveness
evm.model.Ga05G2977	1312	9	+	defense and stress responsiveness
evm.model.Ga06G2190	43	6	+	drought inducibility
evm.model.Ga06G2190	619	9	+	light responsiveness
evm.model.Ga06G2190	236	7	-	auxin responsiveness

evm.model.Ga06G2190	1985	7	-	gibberellin responsiveness
evm.model.Ga06G2190	1805	7	-	light responsiveness
evm.model.Ga06G2190	1806	6	-	light responsiveness
evm.model.Ga06G2190	640	6	+	light responsiveness
evm.model.Ga06G2190	1232	6	-	light responsiveness
evm.model.Ga06G2190	1245	6	-	light responsiveness
evm.model.Ga06G2190	1340	6	-	light responsiveness
evm.model.Ga06G2190	1785	6	-	light responsiveness
evm.model.Ga06G2190	971	6	-	light responsiveness
evm.model.Ga06G2190	960	6	-	anaerobic induction
evm.model.Ga06G2190	1205	6	-	anaerobic induction
evm.model.Ga06G2190	1086	6	-	auxin responsiveness
evm.model.Ga06G2190	1512	6	+	auxin responsiveness
evm.model.Ga06G2190	1291	9	+	light responsiveness
evm.model.Ga08G2367	199	6	-	light responsiveness
evm.model.Ga08G2367	358	9	-	defense and stress responsiveness
evm.model.Ga08G2367	1047	6	-	light responsiveness
evm.model.Ga08G2367	1581	6	-	light responsiveness
evm.model.Ga08G2367	1664	6	-	light responsiveness
evm.model.Ga08G2367	200	5	+	abscisic acid responsiveness
evm.model.Ga08G2367	229	6	+	light responsiveness
evm.model.Ga08G2367	275	6	+	light responsiveness
evm.model.Ga08G2367	526	6	+	light responsiveness
evm.model.Ga08G2367	1385	6	-	light responsiveness
evm.model.Ga08G2367	1965	8	-	light responsiveness
evm.model.Ga08G2367	885	6	-	light responsiveness
evm.model.Ga08G2367	558	6	+	anaerobic induction
evm.model.Ga08G2367	674	6	+	anaerobic induction
evm.model.Ga08G2367	1818	6	-	anaerobic induction
evm.model.Ga09G2394	147	6	+	light responsiveness
evm.model.Ga09G2394	579	6	+	light responsiveness
evm.model.Ga09G2394	737	6	+	light responsiveness
evm.model.Ga09G2394	1373	6	-	light responsiveness
evm.model.Ga09G2394	1042	6	+	light responsiveness
evm.model.Ga09G2394	873	6	+	anaerobic induction
evm.model.Ga09G2394	1907	6	-	anaerobic induction
evm.model.Ga09G2394	1778	9	+	salicylic acid responsiveness
evm.model.Ga09G2394	1163	6	-	light responsiveness
evm.model.Ga09G2394	966	8	+	light responsiveness
evm.model.Ga09G2394	147	6	+	abscisic acid responsiveness

evm.model.Ga09G2394	148	5	+	abscisic acid responsiveness
evm.model.Ga09G2394	580	5	+	abscisic acid responsiveness
evm.model.Ga09G2394	690	7	+	abscisic acid responsiveness
evm.model.Ga09G2394	737	5	-	abscisic acid responsiveness
evm.model.Ga09G2394	1366	5	+	abscisic acid responsiveness
evm.model.Ga09G2394	1373	5	-	abscisic acid responsiveness
evm.model.Ga09G2394	523	5	+	MeJA responsiveness
evm.model.Ga09G2394	763	5	-	MeJA responsiveness
evm.model.Ga09G2394	1062	5	+	MeJA responsiveness
evm.model.Ga09G2394	1450	7	-	gibberellin responsiveness
evm.model.Ga09G2394	1140	13	-	light responsiveness
evm.model.Ga09G2394	147	6	+	light responsiveness
evm.model.Ga09G2394	1365	6	-	light responsiveness
evm.model.Ga09G2394	523	5	-	MeJA responsiveness
evm.model.Ga09G2394	763	5	+	MeJA responsiveness
evm.model.Ga09G2394	1062	5	-	MeJA responsiveness
evm.model.Ga09G2394	827	6	-	low-temperature responsiveness
evm.model.Ga09G2394	1969	6	-	low-temperature responsiveness
evm.model.Ga10G0927	460	6	+	light responsiveness
evm.model.Ga10G0927	813	6	+	light responsiveness
evm.model.Ga10G0927	838	6	+	light responsiveness
evm.model.Ga10G0927	1401	6	-	light responsiveness
evm.model.Ga10G0927	1698	6	-	light responsiveness
evm.model.Ga10G0927	563	8	+	light responsiveness
evm.model.Ga10G0927	1312	6	+	light responsiveness
evm.model.Ga10G0927	731	9	+	abscisic acid responsiveness
evm.model.Ga10G0927	1755	8	-	abscisic acid responsiveness
evm.model.Ga10G0927	1757	5	-	abscisic acid responsiveness
evm.model.Ga10G0927	747	9	-	salicylic acid responsiveness
evm.model.Ga10G0927	1739	8	+	light responsiveness
evm.model.Ga10G0927	1757	6	-	light responsiveness
evm.model.Ga10G0927	1855	6	-	auxin responsiveness
evm.model.Ga10G0927	1755	9	+	light responsiveness
evm.model.Ga10G0927	821	7	-	light responsiveness
evm.model.Ga10G0927	1046	7	+	auxin responsiveness
evm.model.Ga10G1669	1773	9.5	-	light responsiveness
evm.model.Ga10G1669	489	9	-	light responsiveness
evm.model.Ga10G1669	1532	9	-	wound responsiveness
evm.model.Ga10G1669	1340	9	+	salicylic acid responsiveness
evm.model.Ga10G1669	1652	9	-	salicylic acid responsiveness

evm.model.Ga10G1669	1288	10	+	light responsiveness
evm.model.Ga10G1669	162	6	-	anaerobic induction
evm.model.Ga10G1669	264	6	+	anaerobic induction
evm.model.Ga10G1669	536	6	+	anaerobic induction
evm.model.Ga10G1669	950	6	+	anaerobic induction
evm.model.Ga10G1669	1785	6	+	anaerobic induction
evm.model.Ga10G1669	745	5	-	abscisic acid responsiveness
evm.model.Ga10G1669	136	6	+	light responsiveness
evm.model.Ga10G1669	447	6	+	light responsiveness
evm.model.Ga10G1669	1275	9	+	light responsiveness
evm.model.Ga10G1669	745	6	+	light responsiveness
evm.model.Ga11G0563	1323	6	+	light responsiveness
evm.model.Ga11G0563	1960	7	-	light responsiveness
evm.model.Ga11G0563	1961	6	-	light responsiveness
evm.model.Ga11G0563	215	5	-	abscisic acid responsiveness
evm.model.Ga11G0563	917	5	+	abscisic acid responsiveness
evm.model.Ga11G0563	1286	5	-	abscisic acid responsiveness
evm.model.Ga11G0563	822	7	+	gibberellin responsiveness
evm.model.Ga11G0563	510	6	+	light responsiveness
evm.model.Ga11G0563	644	6	+	light responsiveness
evm.model.Ga11G0563	1764	6	-	light responsiveness
evm.model.Ga11G0563	1768	6	-	light responsiveness
evm.model.Ga11G0563	1443	7	+	light responsiveness
evm.model.Ga11G0563	972	5	+	MeJA responsiveness
evm.model.Ga11G0563	1146	5	+	MeJA responsiveness
evm.model.Ga11G0563	916	6	+	light responsiveness
evm.model.Ga11G0563	1286	6	-	light responsiveness
evm.model.Ga11G0563	755	9	+	salicylic acid responsiveness
evm.model.Ga11G0563	798	9	-	salicylic acid responsiveness
evm.model.Ga11G0563	934	6	-	anaerobic induction
evm.model.Ga11G0563	140	9	-	light responsiveness
evm.model.Ga11G0563	972	5	-	MeJA responsiveness
evm.model.Ga11G0563	1146	5	-	MeJA responsiveness
evm.model.Ga11G0563	215	6	+	light responsiveness
evm.model.Ga11G0563	221	6	+	low-temperature responsiveness
evm.model.Ga11G0563	248	13	-	light responsiveness
evm.model.Ga13G0339	935	6	-	anaerobic induction
evm.model.Ga13G0339	1520	6	+	anaerobic induction
evm.model.Ga13G0339	1571	6	-	anaerobic induction
evm.model.Ga13G0339	1866	6	-	anaerobic induction

evm.model.Ga13G0339	842	9	+	wound responsiveness
evm.model.Ga13G0339	1483	6	-	light responsiveness
evm.model.Ga13G0339	1200	6	-	auxin responsiveness
evm.model.Ga13G0339	1185	7	-	light responsiveness
evm.model.Ga13G0339	112	5	-	MeJA responsiveness
evm.model.Ga13G0339	987	6	+	light responsiveness
evm.model.Ga13G0339	1575	6	-	light responsiveness
evm.model.Ga13G0339	1352	9	+	defense and stress responsiveness
evm.model.Ga13G0339	1303	8	-	light responsiveness
evm.model.Ga13G0339	509	6	+	low-temperature responsiveness
evm.model.Ga13G0339	112	5	+	MeJA responsiveness
evm.model.Ga13G1080	1473	8	+	light responsiveness
evm.model.Ga13G1080	128	5	-	MeJA responsiveness
evm.model.Ga13G1080	1946	9	+	salicylic acid responsiveness
evm.model.Ga13G1080	31	6	-	anaerobic induction
evm.model.Ga13G1080	653	6	-	anaerobic induction
evm.model.Ga13G1080	1201	6	+	anaerobic induction
evm.model.Ga13G1080	1979	6	-	light responsiveness
evm.model.Ga13G1080	1811	8	-	light responsiveness
evm.model.Ga13G1080	1380	6	+	light responsiveness
evm.model.Ga13G1080	1445	10	+	light responsiveness
evm.model.Ga13G1080	348	7	-	gibberellin responsiveness
evm.model.Ga13G1080	128	5	+	MeJA responsiveness
evm.model.Ga13G1080	1381	5	+	abscisic acid responsiveness
evm.model.Ga13G1080	132	7	+	light responsiveness
evm.model.Ga13G1080	1919	6	+	light responsiveness
evm.model.Ga13G1080	10	6	+	light responsiveness
evm.model.Ga13G1080	23	6	+	light responsiveness
evm.model.Ga13G1080	267	6	+	light responsiveness
evm.model.Ga13G1080	293	6	+	light responsiveness
evm.model.Ga13G1858	101	9	-	defense and stress responsiveness
evm.model.Ga13G1858	1931	5	-	MeJA responsiveness
evm.model.Ga13G1858	1094	6	-	light responsiveness
evm.model.Ga13G1858	1465	6	-	light responsiveness
evm.model.Ga13G1858	367	6	+	low-temperature responsiveness
evm.model.Ga13G1858	1138	8	+	light responsiveness
evm.model.Ga13G1858	716	7	-	light responsiveness
evm.model.Ga13G1858	1094	6	-	light responsiveness
evm.model.Ga13G1858	1465	6	-	light responsiveness
evm.model.Ga13G1858	1859	6	-	anaerobic induction

evm.model.Ga13G1858	1917	6	-	anaerobic induction
evm.model.Ga13G1858	1094	6	-	abscisic acid responsiveness
evm.model.Ga13G1858	1095	5	+	abscisic acid responsiveness
evm.model.Ga13G1858	1465	6	-	abscisic acid responsiveness
evm.model.Ga13G1858	1466	5	+	abscisic acid responsiveness
evm.model.Ga13G1858	1344	6	+	light responsiveness
evm.model.Ga13G1858	215	6	+	light responsiveness
evm.model.Ga13G1858	859	6	+	light responsiveness
evm.model.Ga13G1858	1109	6	-	light responsiveness
evm.model.Ga13G1858	1700	6	-	light responsiveness
evm.model.Ga13G1858	1931	5	+	MeJA responsiveness
evm.model.Ga14G0186	319	5	+	MeJA responsiveness
evm.model.Ga14G0186	1197	9	-	light responsiveness
evm.model.Ga14G0186	1153	6	-	light responsiveness
evm.model.Ga14G0186	575	6	+	low-temperature responsiveness
evm.model.Ga14G0186	966	6	-	drought inducibility
evm.model.Ga14G0186	906	7	-	light responsiveness
evm.model.Ga14G0186	513	6	-	light responsiveness
evm.model.Ga14G0186	603	6	+	light responsiveness
evm.model.Ga14G0186	1589	6	-	light responsiveness
evm.model.Ga14G0186	1151	8	+	light responsiveness
evm.model.Ga14G0186	1289	6	-	anaerobic induction
evm.model.Ga14G0186	1871	6	-	anaerobic induction
evm.model.Ga14G0186	1679	7	+	gibberellin responsiveness
evm.model.Ga14G0186	1577	6	-	light responsiveness
evm.model.Ga14G0186	1154	5	+	abscisic acid responsiveness
evm.model.Ga14G0186	316	8	-	auxin responsiveness
evm.model.Ga14G0186	22	6	+	light responsiveness
evm.model.Ga14G0186	43	6	+	light responsiveness
evm.model.Ga14G0186	132	6	+	light responsiveness
evm.model.Ga14G0186	143	6	+	light responsiveness
evm.model.Ga14G0186	359	6	+	light responsiveness
evm.model.Ga14G0186	319	5	-	MeJA responsiveness

Table S4b. cis elements in Gb RF2 genes

Gbar_A01G004970.1	68	6	+	light responsiveness
Gbar_A01G004970.1	314	6	+	light responsiveness
Gbar_A01G004970.1	516	6	+	light responsiveness
Gbar_A01G004970.1	586	6	+	light responsiveness
Gbar_A01G004970.1	607	6	+	light responsiveness
Gbar_A01G004970.1	627	6	+	light responsiveness
Gbar_A01G004970.1	769	6	+	light responsiveness
Gbar_A01G004970.1	1600	6	-	light responsiveness
Gbar_A01G004970.1	1122	7	-	auxin responsiveness
Gbar_A01G004970.1	1777	6	-	light responsiveness
Gbar_A01G004970.1	1600	6	-	light responsiveness
Gbar_A01G004970.1	1868	6	-	light responsiveness
Gbar_A01G004970.1	1006	7	+	light responsiveness
Gbar_A01G004970.1	329	6	+	anaerobic induction
Gbar_A01G004970.1	1038	6	-	anaerobic induction
Gbar_A01G004970.1	1600	6	-	abscisic acid responsiveness
Gbar_A01G004970.1	1601	5	+	abscisic acid responsiveness
Gbar_A02G001100.1	252	6	+	light responsiveness
Gbar_A02G001100.1	1005	5	-	MeJA responsiveness
Gbar_A02G001100.1	332	6	+	light responsiveness
Gbar_A02G001100.1	932	6	+	light responsiveness
Gbar_A02G001100.1	952	6	+	light responsiveness
Gbar_A02G001100.1	1253	6	-	light responsiveness
Gbar_A02G001100.1	1343	6	-	light responsiveness
Gbar_A02G001100.1	1498	6	-	light responsiveness
Gbar_A02G001100.1	1605	6	-	light responsiveness
Gbar_A02G001100.1	1005	5	+	MeJA responsiveness
Gbar_A02G001100.1	1725	9	+	salicylic acid responsiveness
Gbar_A02G001100.1	882	6	-	auxin responsiveness
Gbar_A02G001100.1	252	5	-	abscisic acid responsiveness
Gbar_A02G001100.1	834	6	+	low-temperature responsiveness
Gbar_A02G001100.1	345	6	-	light responsiveness
Gbar_A02G001100.1	793	6	+	light responsiveness
Gbar_A02G001100.1	252	8	-	light responsiveness
Gbar_A02G006540.1	303	7	-	light responsiveness
Gbar_A02G006540.1	1959	10	+	light responsiveness
Gbar_A02G006540.1	864	5	+	MeJA responsiveness
Gbar_A02G006540.1	465	6	-	light responsiveness
Gbar_A02G006540.1	1705	6	-	light responsiveness

Gbar_A02G006540.1	297	6	-	auxin responsiveness
Gbar_A02G006540.1	144	10	+	light responsiveness
Gbar_A02G006540.1	283	10	-	light responsiveness
Gbar_A02G006540.1	354	6	-	light responsiveness
Gbar_A02G006540.1	1465	6	-	light responsiveness
Gbar_A02G006540.1	1482	6	+	light responsiveness
Gbar_A02G006540.1	73	8	+	light responsiveness
Gbar_A02G006540.1	1937	9	+	defense and stress responsiveness
Gbar_A02G006540.1	864	5	-	MeJA responsiveness
Gbar_A02G006540.1	1448	6	+	light responsiveness
Gbar_A02G006540.1	1548	6	-	light responsiveness
Gbar_A02G006540.1	1917	6	+	light responsiveness
Gbar_A02G006540.1	189	9	-	light responsiveness
Gbar_A02G006540.1	190	6	+	light responsiveness
Gbar_A02G006540.1	865	6	-	light responsiveness
Gbar_A02G006540.1	1463	8	+	light responsiveness
Gbar_A02G006540.1	191	5	+	abscisic acid responsiveness
Gbar_A02G006540.1	355	5	+	abscisic acid responsiveness
Gbar_A02G006540.1	866	5	+	abscisic acid responsiveness
Gbar_A02G006540.1	1466	5	+	abscisic acid responsiveness
Gbar_A02G006540.1	1482	5	-	abscisic acid responsiveness
Gbar_A02G006540.1	788	6	-	drought inducibility
Gbar_A03G009220.1	166	6	+	light responsiveness
Gbar_A03G009220.1	770	6	-	light responsiveness
Gbar_A03G009220.1	1225	6	+	light responsiveness
Gbar_A03G009220.1	1054	6	-	auxin responsiveness
Gbar_A03G009220.1	353	6	+	light responsiveness
Gbar_A03G009220.1	665	6	+	light responsiveness
Gbar_A03G009220.1	811	6	+	light responsiveness
Gbar_A03G009220.1	644	8	-	light responsiveness
Gbar_A03G009220.1	314	6	-	light responsiveness
Gbar_A03G009220.1	1953	9.5	-	light responsiveness
Gbar_A03G009220.1	55	13	+	light responsiveness
Gbar_A03G009220.1	1001	6	-	anaerobic induction
Gbar_A03G009220.1	1241	7	+	gibberellin responsiveness
Gbar_A03G009220.1	268	6	-	light responsiveness
Gbar_A03G009220.1	229	7	-	light responsiveness
Gbar_A03G009220.1	515	7	+	light responsiveness
Gbar_A03G009220.1	244	6	-	light responsiveness
Gbar_A03G009220.1	847	6	+	light responsiveness

Gbar_A03G009220.1	1697	6	-	light responsiveness
Gbar_A03G009220.1	343	6	+	drought inducibility
Gbar_A03G009220.1	1982	6	-	drought inducibility
Gbar_A03G009220.1	1242	10	+	light responsiveness
Gbar_A03G009220.1	244	5	-	abscisic acid responsiveness
Gbar_A03G009220.1	315	5	+	abscisic acid responsiveness
Gbar_A03G021150.1	1081	6	-	light responsiveness
Gbar_A03G021150.1	1944	6	-	light responsiveness
Gbar_A03G021150.1	545	7	-	light responsiveness
Gbar_A03G021150.1	1671	7	-	light responsiveness
Gbar_A03G021150.1	793	10	-	light responsiveness
Gbar_A03G021150.1	840	10	-	light responsiveness
Gbar_A03G021150.1	583	9	-	salicylic acid responsiveness
Gbar_A03G021150.1	864	9	+	salicylic acid responsiveness
Gbar_A03G021150.1	409	6	+	light responsiveness
Gbar_A03G021150.1	7	9	+	light responsiveness
Gbar_A03G021150.1	47	6	-	auxin responsiveness
Gbar_A03G021150.1	1668	7	+	light responsiveness
Gbar_A03G021150.1	1892	9	-	light responsiveness
Gbar_A03G021150.1	846	6	+	light responsiveness
Gbar_A03G021150.1	1126	6	+	low-temperature responsiveness
Gbar_A03G021150.1	690	6	-	light responsiveness
Gbar_A03G021150.1	1918	10	-	light responsiveness
Gbar_A03G021150.1	1711	6	-	anaerobic induction
Gbar_A03G021150.1	1854	6	-	anaerobic induction
Gbar_A03G021900.1	4	6	+	light responsiveness
Gbar_A03G021900.1	702	6	+	light responsiveness
Gbar_A03G021900.1	1030	6	-	light responsiveness
Gbar_A03G021900.1	1214	6	-	light responsiveness
Gbar_A03G021900.1	122	5	-	MeJA responsiveness
Gbar_A03G021900.1	217	5	-	MeJA responsiveness
Gbar_A03G021900.1	329	7	+	light responsiveness
Gbar_A03G021900.1	1964	6	+	light responsiveness
Gbar_A03G021900.1	1460	10	+	light responsiveness
Gbar_A03G021900.1	1242	9	-	defense and stress responsiveness
Gbar_A03G021900.1	1728	8	-	light responsiveness
Gbar_A03G021900.1	1905	8	-	light responsiveness
Gbar_A03G021900.1	122	5	+	MeJA responsiveness
Gbar_A03G021900.1	217	5	+	MeJA responsiveness
Gbar_A03G021900.1	711	14	+	light responsiveness

Gbar_A03G021900.1	671	6	+	anaerobic induction
Gbar_A03G021900.1	1588	6	+	light responsiveness
Gbar_A03G021900.1	1589	5	+	abscisic acid responsiveness
Gbar_A03G021900.1	357	6	+	drought inducibility
Gbar_A05G026560.1	1781	5	+	MeJA responsiveness
Gbar_A05G026560.1	1957	5	+	MeJA responsiveness
Gbar_A05G026560.1	1765	13	-	light responsiveness
Gbar_A05G026560.1	1778	8	-	auxin responsiveness
Gbar_A05G026560.1	101	6	+	light responsiveness
Gbar_A05G026560.1	1095	6	-	light responsiveness
Gbar_A05G026560.1	1119	6	-	light responsiveness
Gbar_A05G026560.1	1781	5	-	MeJA responsiveness
Gbar_A05G026560.1	1957	5	-	MeJA responsiveness
Gbar_A05G026560.1	797	10	-	light responsiveness
Gbar_A05G026560.1	1199	6	+	drought inducibility
Gbar_A05G026560.1	1848	5	+	abscisic acid responsiveness
Gbar_A05G026560.1	1536	10	+	light responsiveness
Gbar_A05G026560.1	419	6	+	anaerobic induction
Gbar_A05G026560.1	1847	6	+	light responsiveness
Gbar_A05G026560.1	1612	6	+	light responsiveness
Gbar_A05G026560.1	1911	6	+	light responsiveness
Gbar_A05G026560.1	1969	6	+	low-temperature responsiveness
Gbar_A05G027450.1	752	5	-	abscisic acid responsiveness
Gbar_A05G027450.1	1420	9	-	circadian control
Gbar_A05G027450.1	752	6	+	light responsiveness
Gbar_A05G027450.1	203	6	-	anaerobic induction
Gbar_A05G027450.1	1532	6	-	anaerobic induction
Gbar_A05G027450.1	1764	6	+	anaerobic induction
Gbar_A05G027450.1	1899	6	+	low-temperature responsiveness
Gbar_A05G027450.1	1488	7	-	gibberellin responsiveness
Gbar_A05G027450.1	241	5	-	MeJA responsiveness
Gbar_A05G027450.1	754	5	+	MeJA responsiveness
Gbar_A05G027450.1	1474	8	+	light responsiveness
Gbar_A05G027450.1	1325	9.5	-	light responsiveness
Gbar_A05G027450.1	295	8	+	light responsiveness
Gbar_A05G027450.1	12	10	+	light responsiveness
Gbar_A05G027450.1	1222	9	+	defense and stress responsiveness
Gbar_A05G027450.1	241	5	+	MeJA responsiveness
Gbar_A05G027450.1	754	5	-	MeJA responsiveness
Gbar_A05G027450.1	119	6	+	light responsiveness

Gbar_A05G027450.1	791	6	+	light responsiveness
Gbar_A05G027450.1	923	6	+	light responsiveness
Gbar_A05G027450.1	1020	6	-	light responsiveness
Gbar_A05G027450.1	1035	6	-	light responsiveness
Gbar_A05G027450.1	1090	6	-	light responsiveness
Gbar_A05G040630.1	890	7	-	abscisic acid responsiveness
Gbar_A05G040630.1	1291	6	+	light responsiveness
Gbar_A05G040630.1	1206	6	+	anaerobic induction
Gbar_A05G040630.1	1592	6	-	anaerobic induction
Gbar_A05G040630.1	1369	13	-	light responsiveness
Gbar_A05G040630.1	972	5	+	MeJA responsiveness
Gbar_A05G040630.1	1486	8	+	light responsiveness
Gbar_A05G040630.1	1686	8	+	light responsiveness
Gbar_A05G040630.1	1553	8	-	light responsiveness
Gbar_A05G040630.1	1287	9	+	defense and stress responsiveness
Gbar_A05G040630.1	972	5	-	MeJA responsiveness
Gbar_A05G040630.1	575	6	+	light responsiveness
Gbar_A05G040630.1	1501	6	-	light responsiveness
Gbar_A05G040630.1	1887	6	-	auxin responsiveness
Gbar_A05G040630.1	1100	6	-	light responsiveness
Gbar_A06G019370.1	638	9	+	light responsiveness
Gbar_A06G019370.1	1105	6	-	auxin responsiveness
Gbar_A06G019370.1	1510	6	+	auxin responsiveness
Gbar_A06G019370.1	1805	7	-	light responsiveness
Gbar_A06G019370.1	1806	6	-	light responsiveness
Gbar_A06G019370.1	659	6	+	light responsiveness
Gbar_A06G019370.1	1251	6	-	light responsiveness
Gbar_A06G019370.1	1264	6	-	light responsiveness
Gbar_A06G019370.1	1785	6	-	light responsiveness
Gbar_A06G019370.1	256	7	-	auxin responsiveness
Gbar_A06G019370.1	990	6	-	light responsiveness
Gbar_A06G019370.1	979	6	-	anaerobic induction
Gbar_A06G019370.1	1224	6	-	anaerobic induction
Gbar_A06G019370.1	1985	7	-	gibberellin responsiveness
Gbar_A06G019370.1	1304	9	+	light responsiveness
Gbar_A06G019370.1	63	6	+	drought inducibility
Gbar_A07G025540.1	40	5	+	MeJA responsiveness
Gbar_A07G025540.1	1931	5	+	MeJA responsiveness
Gbar_A07G025540.1	261	6	+	light responsiveness
Gbar_A07G025540.1	888	6	+	light responsiveness

Gbar_A07G025540.1	1700	6	-	light responsiveness
Gbar_A07G025540.1	1342	6	+	light responsiveness
Gbar_A07G025540.1	1137	8	+	light responsiveness
Gbar_A07G025540.1	40	5	-	MeJA responsiveness
Gbar_A07G025540.1	1931	5	-	MeJA responsiveness
Gbar_A07G025540.1	1093	6	-	light responsiveness
Gbar_A07G025540.1	1464	6	-	light responsiveness
Gbar_A07G025540.1	413	6	+	low-temperature responsiveness
Gbar_A07G025540.1	1093	6	-	light responsiveness
Gbar_A07G025540.1	1464	6	-	light responsiveness
Gbar_A07G025540.1	764	7	-	light responsiveness
Gbar_A07G025540.1	1859	6	-	anaerobic induction
Gbar_A07G025540.1	1917	6	-	anaerobic induction
Gbar_A07G025540.1	1093	6	-	abscisic acid responsiveness
Gbar_A07G025540.1	1094	5	+	abscisic acid responsiveness
Gbar_A07G025540.1	1464	6	-	abscisic acid responsiveness
Gbar_A07G025540.1	1465	5	+	abscisic acid responsiveness
Gbar_A09G022230.1	1146	13	-	light responsiveness
Gbar_A09G022230.1	1777	9	+	salicylic acid responsiveness
Gbar_A09G022230.1	768	5	-	MeJA responsiveness
Gbar_A09G022230.1	1068	5	+	MeJA responsiveness
Gbar_A09G022230.1	2	5	+	abscisic acid responsiveness
Gbar_A09G022230.1	585	5	+	abscisic acid responsiveness
Gbar_A09G022230.1	695	7	+	abscisic acid responsiveness
Gbar_A09G022230.1	742	5	-	abscisic acid responsiveness
Gbar_A09G022230.1	1378	5	-	abscisic acid responsiveness
Gbar_A09G022230.1	584	6	+	light responsiveness
Gbar_A09G022230.1	742	6	+	light responsiveness
Gbar_A09G022230.1	1378	6	-	light responsiveness
Gbar_A09G022230.1	91	6	+	anoxic specific inducibility
Gbar_A09G022230.1	1	6	-	light responsiveness
Gbar_A09G022230.1	971	8	+	light responsiveness
Gbar_A09G022230.1	768	5	+	MeJA responsiveness
Gbar_A09G022230.1	1068	5	-	MeJA responsiveness
Gbar_A09G022230.1	1169	6	-	light responsiveness
Gbar_A09G022230.1	1454	7	-	gibberellin responsiveness
Gbar_A09G022230.1	878	6	+	anaerobic induction
Gbar_A09G022230.1	1907	6	-	anaerobic induction
Gbar_A09G022230.1	832	6	-	low-temperature responsiveness
Gbar_A09G022230.1	1969	6	-	low-temperature responsiveness

Gbar_A09G022230.1	1048	6	+	light responsiveness
Gbar_A10G014980.1	256	6	-	anaerobic induction
Gbar_A10G014980.1	359	6	+	anaerobic induction
Gbar_A10G014980.1	631	6	+	anaerobic induction
Gbar_A10G014980.1	1789	6	+	anaerobic induction
Gbar_A10G014980.1	1536	9	-	wound responsiveness
Gbar_A10G014980.1	751	8	+	light responsiveness
Gbar_A10G014980.1	1293	10	+	light responsiveness
Gbar_A10G014980.1	1280	9	+	light responsiveness
Gbar_A10G014980.1	842	6	+	light responsiveness
Gbar_A10G014980.1	1025	6	+	light responsiveness
Gbar_A10G014980.1	1345	9	+	salicylic acid responsiveness
Gbar_A10G014980.1	1656	9	-	salicylic acid responsiveness
Gbar_A10G014980.1	230	6	+	light responsiveness
Gbar_A10G014980.1	542	6	+	light responsiveness
Gbar_A10G014980.1	1777	9.5	-	light responsiveness
Gbar_A10G019270.1	559	8	+	light responsiveness
Gbar_A10G019270.1	456	6	+	light responsiveness
Gbar_A10G019270.1	813	6	+	light responsiveness
Gbar_A10G019270.1	838	6	+	light responsiveness
Gbar_A10G019270.1	1401	6	-	light responsiveness
Gbar_A10G019270.1	1698	6	-	light responsiveness
Gbar_A10G019270.1	821	7	-	light responsiveness
Gbar_A10G019270.1	1855	6	-	auxin responsiveness
Gbar_A10G019270.1	1312	6	+	light responsiveness
Gbar_A10G019270.1	747	9	-	salicylic acid responsiveness
Gbar_A10G019270.1	731	9	+	abscisic acid responsiveness
Gbar_A10G019270.1	1755	8	-	abscisic acid responsiveness
Gbar_A10G019270.1	1757	5	-	abscisic acid responsiveness
Gbar_A10G019270.1	1739	8	+	light responsiveness
Gbar_A10G019270.1	1755	9	+	light responsiveness
Gbar_A10G019270.1	1757	6	-	light responsiveness
Gbar_A11G030850.1	1450	7	+	light responsiveness
Gbar_A11G030850.1	1723	7	+	light responsiveness
Gbar_A11G030850.1	214	6	+	drought inducibility
Gbar_A11G030850.1	222	5	-	abscisic acid responsiveness
Gbar_A11G030850.1	924	5	+	abscisic acid responsiveness
Gbar_A11G030850.1	1034	6	-	abscisic acid responsiveness
Gbar_A11G030850.1	1035	5	+	abscisic acid responsiveness
Gbar_A11G030850.1	941	6	-	anaerobic induction

Gbar_A11G030850.1	923	6	+	light responsiveness
Gbar_A11G030850.1	1033	8	-	light responsiveness
Gbar_A11G030850.1	1034	6	-	light responsiveness
Gbar_A11G030850.1	222	6	+	light responsiveness
Gbar_A11G030850.1	1034	6	-	light responsiveness
Gbar_A11G030850.1	1153	5	-	MeJA responsiveness
Gbar_A11G030850.1	255	13	-	light responsiveness
Gbar_A11G030850.1	762	9	+	salicylic acid responsiveness
Gbar_A11G030850.1	1960	7	-	light responsiveness
Gbar_A11G030850.1	1961	6	-	light responsiveness
Gbar_A11G030850.1	147	9	-	light responsiveness
Gbar_A11G030850.1	517	6	+	light responsiveness
Gbar_A11G030850.1	651	6	+	light responsiveness
Gbar_A11G030850.1	1762	6	-	light responsiveness
Gbar_A11G030850.1	1766	6	-	light responsiveness
Gbar_A11G030850.1	1153	5	+	MeJA responsiveness
Gbar_A12G002500.1	520	6	-	light responsiveness
Gbar_A12G002500.1	610	6	+	light responsiveness
Gbar_A12G002500.1	1596	6	-	light responsiveness
Gbar_A12G002500.1	1686	7	+	gibberellin responsiveness
Gbar_A12G002500.1	913	7	-	light responsiveness
Gbar_A12G002500.1	1158	8	+	light responsiveness
Gbar_A12G002500.1	1296	6	-	anaerobic induction
Gbar_A12G002500.1	1871	6	-	anaerobic induction
Gbar_A12G002500.1	1161	5	+	abscisic acid responsiveness
Gbar_A12G002500.1	973	6	-	drought inducibility
Gbar_A12G002500.1	326	5	-	MeJA responsiveness
Gbar_A12G002500.1	28	6	+	light responsiveness
Gbar_A12G002500.1	49	6	+	light responsiveness
Gbar_A12G002500.1	139	6	+	light responsiveness
Gbar_A12G002500.1	150	6	+	light responsiveness
Gbar_A12G002500.1	366	6	+	light responsiveness
Gbar_A12G002500.1	323	8	-	auxin responsiveness
Gbar_A12G002500.1	1584	6	-	light responsiveness
Gbar_A12G002500.1	1204	9	-	light responsiveness
Gbar_A12G002500.1	326	5	+	MeJA responsiveness
Gbar_A12G002500.1	1160	6	-	light responsiveness
Gbar_A13G003620.1	117	5	+	MeJA responsiveness
Gbar_A13G003620.1	1353	9	+	defense and stress responsiveness
Gbar_A13G003620.1	1304	8	-	light responsiveness

Gbar_A13G003620.1	1201	6	-	auxin responsiveness
Gbar_A13G003620.1	1575	6	-	light responsiveness
Gbar_A13G003620.1	117	5	-	MeJA responsiveness
Gbar_A13G003620.1	988	6	+	light responsiveness
Gbar_A13G003620.1	843	9	+	wound responsiveness
Gbar_A13G003620.1	1484	6	-	light responsiveness
Gbar_A13G003620.1	1726	9	-	light responsiveness
Gbar_A13G003620.1	1186	7	-	light responsiveness
Gbar_A13G003620.1	936	6	-	anaerobic induction
Gbar_A13G003620.1	1521	6	+	anaerobic induction
Gbar_A13G003620.1	1571	6	-	anaerobic induction
Gbar_A13G003620.1	1866	6	-	anaerobic induction
Gbar_A13G003620.1	514	6	+	low-temperature responsiveness
Gbar_A13G009350.1	1946	9	+	salicylic acid responsiveness
Gbar_A13G009350.1	135	7	+	light responsiveness
Gbar_A13G009350.1	1919	6	+	light responsiveness
Gbar_A13G009350.1	967	9	+	light responsiveness
Gbar_A13G009350.1	131	5	+	MeJA responsiveness
Gbar_A13G009350.1	14	6	+	light responsiveness
Gbar_A13G009350.1	27	6	+	light responsiveness
Gbar_A13G009350.1	270	6	+	light responsiveness
Gbar_A13G009350.1	290	6	+	light responsiveness
Gbar_A13G009350.1	793	6	+	light responsiveness
Gbar_A13G009350.1	131	5	-	MeJA responsiveness
Gbar_A13G009350.1	1810	8	-	light responsiveness
Gbar_A13G009350.1	1475	8	+	light responsiveness
Gbar_A13G009350.1	1729	8	+	light responsiveness
Gbar_A13G009350.1	1380	6	+	light responsiveness
Gbar_A13G009350.1	35	6	-	anaerobic induction
Gbar_A13G009350.1	652	6	-	anaerobic induction
Gbar_A13G009350.1	1201	6	+	anaerobic induction
Gbar_A13G009350.1	345	7	-	gibberellin responsiveness
Gbar_A13G009350.1	1979	6	-	light responsiveness
Gbar_A13G009350.1	1445	10	+	light responsiveness
Gbar_A13G009350.1	1381	5	+	abscisic acid responsiveness
Gbar_D01G005120.1	394	6	+	anaerobic induction
Gbar_D01G005120.1	1047	6	-	anaerobic induction
Gbar_D01G005120.1	1608	6	-	light responsiveness
Gbar_D01G005120.1	1870	6	-	light responsiveness
Gbar_D01G005120.1	1015	7	+	light responsiveness

Gbar_D01G005120.1	1127	7	-	gibberellin responsiveness
Gbar_D01G005120.1	449	6	+	light responsiveness
Gbar_D01G005120.1	559	5	+	abscisic acid responsiveness
Gbar_D01G005120.1	1608	6	-	abscisic acid responsiveness
Gbar_D01G005120.1	1609	5	+	abscisic acid responsiveness
Gbar_D01G005120.1	312	6	+	auxin responsiveness
Gbar_D01G005120.1	213	6	-	light responsiveness
Gbar_D01G005120.1	149	6	+	light responsiveness
Gbar_D01G005120.1	283	6	+	light responsiveness
Gbar_D01G005120.1	379	6	+	light responsiveness
Gbar_D01G005120.1	583	6	+	light responsiveness
Gbar_D01G005120.1	785	6	+	light responsiveness
Gbar_D01G005120.1	827	6	+	light responsiveness
Gbar_D01G005120.1	9	7	+	light responsiveness
Gbar_D01G005120.1	131	5	+	MeJA responsiveness
Gbar_D01G005120.1	657	13	+	light responsiveness
Gbar_D01G005120.1	558	6	-	light responsiveness
Gbar_D01G005120.1	1608	6	-	light responsiveness
Gbar_D01G005120.1	131	5	-	MeJA responsiveness
Gbar_D02G001400.1	390	6	+	abscisic acid responsiveness
Gbar_D02G001400.1	391	5	+	abscisic acid responsiveness
Gbar_D02G001400.1	584	10	+	light responsiveness
Gbar_D02G001400.1	906	6	+	low-temperature responsiveness
Gbar_D02G001400.1	872	6	+	light responsiveness
Gbar_D02G001400.1	1346	6	-	light responsiveness
Gbar_D02G001400.1	390	6	+	light responsiveness
Gbar_D02G001400.1	11	5	+	MeJA responsiveness
Gbar_D02G001400.1	1049	5	-	MeJA responsiveness
Gbar_D02G001400.1	1928	8	-	light responsiveness
Gbar_D02G001400.1	1386	8	+	light responsiveness
Gbar_D02G001400.1	390	6	+	light responsiveness
Gbar_D02G001400.1	11	5	-	MeJA responsiveness
Gbar_D02G001400.1	1049	5	+	MeJA responsiveness
Gbar_D02G001400.1	1	6	+	light responsiveness
Gbar_D02G001400.1	42	6	+	light responsiveness
Gbar_D02G001400.1	67	6	+	light responsiveness
Gbar_D02G001400.1	1002	6	-	light responsiveness
Gbar_D02G001400.1	1022	6	-	light responsiveness
Gbar_D02G001400.1	1295	6	-	light responsiveness
Gbar_D02G001400.1	1513	6	-	light responsiveness

Gbar_D02G001400.1	1619	6	-	light responsiveness
Gbar_D02G001400.1	76	6	-	light responsiveness
Gbar_D02G001400.1	568	6	+	light responsiveness
Gbar_D02G001400.1	1741	9	+	salicylic acid responsiveness
Gbar_D02G001400.1	242	6	-	auxin responsiveness
Gbar_D02G007490.1	868	5	+	MeJA responsiveness
Gbar_D02G007490.1	517	6	+	light responsiveness
Gbar_D02G007490.1	564	6	+	light responsiveness
Gbar_D02G007490.1	1959	10	+	light responsiveness
Gbar_D02G007490.1	298	6	-	auxin responsiveness
Gbar_D02G007490.1	868	5	-	MeJA responsiveness
Gbar_D02G007490.1	490	13	+	light responsiveness
Gbar_D02G007490.1	287	6	+	light responsiveness
Gbar_D02G007490.1	1401	6	+	light responsiveness
Gbar_D02G007490.1	1937	9	+	defense and stress responsiveness
Gbar_D02G007490.1	1453	6	+	light responsiveness
Gbar_D02G007490.1	1917	6	+	light responsiveness
Gbar_D02G007490.1	1591	7	-	gibberellin responsiveness
Gbar_D02G007490.1	284	11	+	light responsiveness
Gbar_D02G007490.1	287	6	+	light responsiveness
Gbar_D02G007490.1	869	6	-	light responsiveness
Gbar_D02G007490.1	1106	10	-	light responsiveness
Gbar_D02G007490.1	1631	9	+	light responsiveness
Gbar_D02G007490.1	285	9	-	abscisic acid responsiveness
Gbar_D02G007490.1	287	6	+	abscisic acid responsiveness
Gbar_D02G007490.1	288	5	+	abscisic acid responsiveness
Gbar_D02G007490.1	870	5	+	abscisic acid responsiveness
Gbar_D02G007490.1	1401	5	-	abscisic acid responsiveness
Gbar_D02G007490.1	1595	9	-	wound responsiveness
Gbar_D02G007490.1	244	6	+	drought inducibility
Gbar_D02G007490.1	792	6	-	drought inducibility
Gbar_D02G010420.1	398	5	+	abscisic acid responsiveness
Gbar_D02G010420.1	1495	6	+	drought inducibility
Gbar_D02G010420.1	1983	6	-	drought inducibility
Gbar_D02G010420.1	830	6	+	light responsiveness
Gbar_D02G010420.1	1699	6	-	light responsiveness
Gbar_D02G010420.1	1269	7	+	gibberellin responsiveness
Gbar_D02G010420.1	1340	6	+	anaerobic induction
Gbar_D02G010420.1	234	7	-	light responsiveness
Gbar_D02G010420.1	643	8	-	light responsiveness

Gbar_D02G010420.1	397	6	-	light responsiveness
Gbar_D02G010420.1	1938	8	-	light responsiveness
Gbar_D02G010420.1	248	5	-	MeJA responsiveness
Gbar_D02G010420.1	1954	9.5	-	light responsiveness
Gbar_D02G010420.1	248	5	+	MeJA responsiveness
Gbar_D02G010420.1	1253	6	+	light responsiveness
Gbar_D02G010420.1	1897	6	-	light responsiveness
Gbar_D02G010420.1	248	8	+	auxin responsiveness
Gbar_D02G023090.1	151	5	+	MeJA responsiveness
Gbar_D02G023090.1	46	6	-	auxin responsiveness
Gbar_D02G023090.1	579	9	-	salicylic acid responsiveness
Gbar_D02G023090.1	863	9	+	salicylic acid responsiveness
Gbar_D02G023090.1	9	6	-	light responsiveness
Gbar_D02G023090.1	839	10	-	light responsiveness
Gbar_D02G023090.1	539	7	-	light responsiveness
Gbar_D02G023090.1	1671	7	-	light responsiveness
Gbar_D02G023090.1	1944	6	-	light responsiveness
Gbar_D02G023090.1	151	5	-	MeJA responsiveness
Gbar_D02G023090.1	1668	7	+	light responsiveness
Gbar_D02G023090.1	1600	7	+	abscisic acid responsiveness
Gbar_D02G023090.1	1536	6	-	anaerobic induction
Gbar_D02G023090.1	1711	6	-	anaerobic induction
Gbar_D02G023090.1	1854	6	-	anaerobic induction
Gbar_D02G023090.1	1124	6	+	low-temperature responsiveness
Gbar_D02G023090.1	845	6	+	light responsiveness
Gbar_D02G023830.1	716	13	+	light responsiveness
Gbar_D02G023830.1	225	5	+	MeJA responsiveness
Gbar_D02G023830.1	968	5	-	MeJA responsiveness
Gbar_D02G023830.1	986	5	+	MeJA responsiveness
Gbar_D02G023830.1	1721	8	-	light responsiveness
Gbar_D02G023830.1	1898	8	-	light responsiveness
Gbar_D02G023830.1	1262	6	-	light responsiveness
Gbar_D02G023830.1	1290	10	+	light responsiveness
Gbar_D02G023830.1	336	7	+	light responsiveness
Gbar_D02G023830.1	1960	6	+	light responsiveness
Gbar_D02G023830.1	225	5	-	MeJA responsiveness
Gbar_D02G023830.1	968	5	+	MeJA responsiveness
Gbar_D02G023830.1	986	5	-	MeJA responsiveness
Gbar_D02G023830.1	707	6	+	light responsiveness
Gbar_D02G023830.1	717	6	+	light responsiveness

Gbar_D02G023830.1	1227	6	-	light responsiveness
Gbar_D02G023830.1	914	8	-	light responsiveness
Gbar_D02G023830.1	1064	9	+	light responsiveness
Gbar_D02G023830.1	970	5	+	abscisic acid responsiveness
Gbar_D02G023830.1	1263	5	+	abscisic acid responsiveness
Gbar_D02G023830.1	1586	5	+	abscisic acid responsiveness
Gbar_D02G023830.1	969	6	-	light responsiveness
Gbar_D02G023830.1	1585	6	+	light responsiveness
Gbar_D02G023830.1	177	7	-	gibberellin responsiveness
Gbar_D02G023830.1	279	6	-	anaerobic induction
Gbar_D02G023830.1	676	6	+	anaerobic induction
Gbar_D02G023830.1	1039	6	+	anaerobic induction
Gbar_D02G023830.1	233	7	-	gibberellin responsiveness
Gbar_D04G001290.1	1886	6	-	auxin responsiveness
Gbar_D04G001290.1	1126	6	-	light responsiveness
Gbar_D04G001290.1	330	8.5	+	cell cycle regulation
Gbar_D04G001290.1	203	8	+	light responsiveness
Gbar_D04G001290.1	606	6	+	light responsiveness
Gbar_D04G001290.1	1617	6	-	light responsiveness
Gbar_D04G001290.1	997	5	-	MeJA responsiveness
Gbar_D04G001290.1	1306	9	+	defense and stress responsiveness
Gbar_D04G001290.1	1702	8	+	light responsiveness
Gbar_D04G001290.1	997	5	+	MeJA responsiveness
Gbar_D04G001290.1	1449	6	+	light responsiveness
Gbar_D04G001290.1	1310	6	+	light responsiveness
Gbar_D04G001290.1	69	9	+	light responsiveness
Gbar_D04G001290.1	929	6	-	drought inducibility
Gbar_D04G001290.1	1450	5	+	abscisic acid responsiveness
Gbar_D04G017730.1	305	8	-	light responsiveness
Gbar_D04G017730.1	1523	8	-	light responsiveness
Gbar_D04G017730.1	951	5	+	MeJA responsiveness
Gbar_D04G017730.1	951	5	-	MeJA responsiveness
Gbar_D04G017730.1	1383	9	+	light responsiveness
Gbar_D04G017730.1	419	6	+	light responsiveness
Gbar_D04G017730.1	529	6	+	light responsiveness
Gbar_D04G017730.1	523	9	+	cell cycle regulation
Gbar_D04G017730.1	1542	8	-	light responsiveness
Gbar_D04G017730.1	1644	10	-	light responsiveness
Gbar_D04G017730.1	1215	7	-	gibberellin responsiveness
Gbar_D04G017730.1	123	6	-	light responsiveness

Gbar_D04G017730.1	480	6	+	low-temperature responsiveness
Gbar_D04G017730.1	1264	7	-	gibberellin responsiveness
Gbar_D04G017740.1	295	6	-	light responsiveness
Gbar_D04G017740.1	1425	6	-	light responsiveness
Gbar_D04G017740.1	877	6	-	light responsiveness
Gbar_D04G017740.1	1883	6	-	anaerobic induction
Gbar_D04G017740.1	607	9	-	circadian control
Gbar_D04G017740.1	449	7	+	abscisic acid responsiveness
Gbar_D04G017740.1	878	5	+	abscisic acid responsiveness
Gbar_D04G017740.1	860	8	-	light responsiveness
Gbar_D04G017740.1	876	5	+	MeJA responsiveness
Gbar_D04G017740.1	398	6	+	light responsiveness
Gbar_D04G017740.1	628	6	+	light responsiveness
Gbar_D04G017740.1	1002	6	-	light responsiveness
Gbar_D04G017740.1	1661	8	-	light responsiveness
Gbar_D04G017740.1	232	7	-	light responsiveness
Gbar_D04G017740.1	233	6	-	light responsiveness
Gbar_D04G017740.1	382	7	-	light responsiveness
Gbar_D04G017740.1	383	6	-	light responsiveness
Gbar_D04G017740.1	892	6	+	light responsiveness
Gbar_D04G017740.1	1080	6	+	light responsiveness
Gbar_D04G017740.1	761	8	+	light responsiveness
Gbar_D04G017740.1	876	5	-	MeJA responsiveness
Gbar_D04G017740.1	1664	8	+	light responsiveness
Gbar_D04G017740.1	1425	9	-	defense and stress responsiveness
Gbar_D05G027400.1	1969	6	+	low-temperature responsiveness
Gbar_D05G027400.1	528	6	+	light responsiveness
Gbar_D05G027400.1	1911	6	+	light responsiveness
Gbar_D05G027400.1	700	6	+	anaerobic induction
Gbar_D05G027400.1	34	7	+	light responsiveness
Gbar_D05G027400.1	618	6	+	light responsiveness
Gbar_D05G027400.1	1847	6	+	light responsiveness
Gbar_D05G027400.1	1848	5	+	abscisic acid responsiveness
Gbar_D05G027400.1	1266	9	-	light responsiveness
Gbar_D05G027400.1	1538	10	+	light responsiveness
Gbar_D05G027400.1	792	10	-	light responsiveness
Gbar_D05G027400.1	797	10	-	light responsiveness
Gbar_D05G027400.1	1201	6	+	drought inducibility
Gbar_D05G027400.1	431	6	+	light responsiveness
Gbar_D05G027400.1	1092	6	-	light responsiveness

Gbar_D05G027400.1	1117	6	-	light responsiveness
Gbar_D05G027400.1	455	5	-	MeJA responsiveness
Gbar_D05G027400.1	1780	5	-	MeJA responsiveness
Gbar_D05G027400.1	1957	5	-	MeJA responsiveness
Gbar_D05G027400.1	261	9	-	salicylic acid responsiveness
Gbar_D05G027400.1	79	6	+	light responsiveness
Gbar_D05G027400.1	1777	8	-	auxin responsiveness
Gbar_D05G027400.1	1650	8	+	light responsiveness
Gbar_D05G027400.1	1555	6	+	auxin responsiveness
Gbar_D05G027400.1	473	9	-	light responsiveness
Gbar_D05G027400.1	455	5	+	MeJA responsiveness
Gbar_D05G027400.1	1780	5	+	MeJA responsiveness
Gbar_D05G027400.1	1957	5	+	MeJA responsiveness
Gbar_D05G027400.1	1764	13	-	light responsiveness
Gbar_D05G028280.1	1914	6	+	low-temperature responsiveness
Gbar_D05G028280.1	46	6	-	anaerobic induction
Gbar_D05G028280.1	545	6	-	anaerobic induction
Gbar_D05G028280.1	1531	6	+	anaerobic induction
Gbar_D05G028280.1	1544	6	+	anaerobic induction
Gbar_D05G028280.1	1583	6	-	anaerobic induction
Gbar_D05G028280.1	1780	6	+	anaerobic induction
Gbar_D05G028280.1	761	8	+	light responsiveness
Gbar_D05G028280.1	208	6	+	light responsiveness
Gbar_D05G028280.1	732	6	+	light responsiveness
Gbar_D05G028280.1	775	6	+	light responsiveness
Gbar_D05G028280.1	1021	6	-	light responsiveness
Gbar_D05G028280.1	1404	6	-	light responsiveness
Gbar_D05G028280.1	84	5	+	MeJA responsiveness
Gbar_D05G028280.1	1869	9	+	salicylic acid responsiveness
Gbar_D05G028280.1	1747	8	+	light responsiveness
Gbar_D05G028280.1	739	9	-	light responsiveness
Gbar_D05G028280.1	1273	9.5	-	light responsiveness
Gbar_D05G028280.1	84	5	-	MeJA responsiveness
Gbar_D06G020250.1	1985	7	-	gibberellin responsiveness
Gbar_D06G020250.1	346	6	+	light responsiveness
Gbar_D06G020250.1	1083	6	+	light responsiveness
Gbar_D06G020250.1	1098	6	-	low-temperature responsiveness
Gbar_D06G020250.1	1345	6	-	anaerobic induction
Gbar_D06G020250.1	1676	6	-	anaerobic induction
Gbar_D06G020250.1	239	9	+	light responsiveness

Gbar_D06G020250.1	971	10	-	light responsiveness
Gbar_D06G020250.1	604	6	+	light responsiveness
Gbar_D06G020250.1	649	6	+	light responsiveness
Gbar_D06G020250.1	672	6	+	light responsiveness
Gbar_D06G020250.1	676	6	+	light responsiveness
Gbar_D06G020250.1	984	6	+	light responsiveness
Gbar_D06G020250.1	1275	6	-	light responsiveness
Gbar_D06G020250.1	1606	6	-	light responsiveness
Gbar_D06G020250.1	1449	6	+	auxin responsiveness
Gbar_D06G020250.1	909	6	-	light responsiveness
Gbar_D06G020250.1	1778	13	-	light responsiveness
Gbar_D09G021980.1	844	6	-	low-temperature responsiveness
Gbar_D09G021980.1	1969	6	-	low-temperature responsiveness
Gbar_D09G021980.1	11	8	+	light responsiveness
Gbar_D09G021980.1	166	6	+	light responsiveness
Gbar_D09G021980.1	880	6	+	light responsiveness
Gbar_D09G021980.1	1907	6	-	anaerobic induction
Gbar_D09G021980.1	14	5	+	abscisic acid responsiveness
Gbar_D09G021980.1	166	6	+	abscisic acid responsiveness
Gbar_D09G021980.1	167	5	+	abscisic acid responsiveness
Gbar_D09G021980.1	878	9	-	abscisic acid responsiveness
Gbar_D09G021980.1	880	6	+	abscisic acid responsiveness
Gbar_D09G021980.1	881	5	+	abscisic acid responsiveness
Gbar_D09G021980.1	1347	5	-	abscisic acid responsiveness
Gbar_D09G021980.1	434	6	+	drought inducibility
Gbar_D09G021980.1	742	6	+	drought inducibility
Gbar_D09G021980.1	1450	6	+	drought inducibility
Gbar_D09G021980.1	1581	7	+	light responsiveness
Gbar_D09G021980.1	1180	6	-	light responsiveness
Gbar_D09G021980.1	1291	6	+	auxin responsiveness
Gbar_D09G021980.1	577	9	+	light responsiveness
Gbar_D09G021980.1	1773	9	+	salicylic acid responsiveness
Gbar_D09G021980.1	1157	13	-	light responsiveness
Gbar_D09G021980.1	13	6	-	light responsiveness
Gbar_D09G021980.1	166	6	+	light responsiveness
Gbar_D09G021980.1	880	6	+	light responsiveness
Gbar_D09G021980.1	1347	6	+	light responsiveness
Gbar_D09G021980.1	524	6	+	anoxic specific inducibility
Gbar_D10G013120.1	1484	6	+	low-temperature responsiveness
Gbar_D10G013120.1	73	6	+	anaerobic induction

Gbar_D10G013120.1	353	6	+	anaerobic induction
Gbar_D10G013120.1	651	6	+	anaerobic induction
Gbar_D10G013120.1	970	6	+	anaerobic induction
Gbar_D10G013120.1	1542	9	-	wound responsiveness
Gbar_D10G013120.1	1522	6	+	drought inducibility
Gbar_D10G013120.1	290	5	+	MeJA responsiveness
Gbar_D10G013120.1	462	6	+	light responsiveness
Gbar_D10G013120.1	1010	6	-	light responsiveness
Gbar_D10G013120.1	785	6	-	auxin responsiveness
Gbar_D10G013120.1	1289	9	+	light responsiveness
Gbar_D10G013120.1	776	7	-	light responsiveness
Gbar_D10G013120.1	777	6	-	light responsiveness
Gbar_D10G013120.1	1662	9	-	salicylic acid responsiveness
Gbar_D10G013120.1	290	5	-	MeJA responsiveness
Gbar_D10G019420.1	264	6	+	anaerobic induction
Gbar_D10G019420.1	1757	6	-	light responsiveness
Gbar_D10G019420.1	1755	8	-	abscisic acid responsiveness
Gbar_D10G019420.1	1757	5	-	abscisic acid responsiveness
Gbar_D10G019420.1	311	6	+	drought inducibility
Gbar_D10G019420.1	1755	9	+	light responsiveness
Gbar_D10G019420.1	1739	8	+	light responsiveness
Gbar_D10G019420.1	678	6	+	light responsiveness
Gbar_D10G019420.1	780	6	+	light responsiveness
Gbar_D10G019420.1	990	6	+	light responsiveness
Gbar_D10G019420.1	1084	6	-	light responsiveness
Gbar_D10G019420.1	1317	6	-	light responsiveness
Gbar_D10G019420.1	1597	6	-	light responsiveness
Gbar_D10G019420.1	1699	6	-	light responsiveness
Gbar_D10G019420.1	443	7	-	light responsiveness
Gbar_D10G019420.1	1855	6	-	auxin responsiveness
Gbar_D10G019420.1	567	6	+	light responsiveness
Gbar_D10G019420.1	654	7	+	light responsiveness
Gbar_D10G019420.1	1229	6	+	light responsiveness
Gbar_D10G019420.1	1432	6	-	light responsiveness
Gbar_D10G019420.1	729	9	-	salicylic acid responsiveness
Gbar_D10G019420.1	549	8	+	light responsiveness
Gbar_D10G019420.1	1842	8	+	light responsiveness
Gbar_D11G031450.1	1045	5	+	abscisic acid responsiveness
Gbar_D11G031450.1	1228	5	+	abscisic acid responsiveness
Gbar_D11G031450.1	181	6	+	drought inducibility

Gbar_D11G031450.1	1465	7	+	light responsiveness
Gbar_D11G031450.1	1724	7	+	light responsiveness
Gbar_D11G031450.1	132	6	+	low-temperature responsiveness
Gbar_D11G031450.1	195	6	+	low-temperature responsiveness
Gbar_D11G031450.1	170	7	-	gibberellin responsiveness
Gbar_D11G031450.1	940	6	-	anaerobic induction
Gbar_D11G031450.1	1044	6	+	light responsiveness
Gbar_D11G031450.1	1227	6	-	light responsiveness
Gbar_D11G031450.1	1511	8	-	light responsiveness
Gbar_D11G031450.1	222	13	-	light responsiveness
Gbar_D11G031450.1	126	5	+	MeJA responsiveness
Gbar_D11G031450.1	978	5	-	MeJA responsiveness
Gbar_D11G031450.1	1152	5	-	MeJA responsiveness
Gbar_D11G031450.1	1226	5	-	MeJA responsiveness
Gbar_D11G031450.1	1359	5	-	MeJA responsiveness
Gbar_D11G031450.1	335	6	+	light responsiveness
Gbar_D11G031450.1	529	6	+	light responsiveness
Gbar_D11G031450.1	661	6	+	light responsiveness
Gbar_D11G031450.1	668	6	+	light responsiveness
Gbar_D11G031450.1	725	6	+	light responsiveness
Gbar_D11G031450.1	1764	6	-	light responsiveness
Gbar_D11G031450.1	1768	6	-	light responsiveness
Gbar_D11G031450.1	126	5	-	MeJA responsiveness
Gbar_D11G031450.1	978	5	+	MeJA responsiveness
Gbar_D11G031450.1	1152	5	+	MeJA responsiveness
Gbar_D11G031450.1	1226	5	+	MeJA responsiveness
Gbar_D11G031450.1	1359	5	+	MeJA responsiveness
Gbar_D11G031450.1	140	9	-	light responsiveness
Gbar_D11G031450.1	1960	7	-	light responsiveness
Gbar_D11G031450.1	1961	6	-	light responsiveness
Gbar_D12G002730.1	918	7	-	light responsiveness
Gbar_D12G002730.1	1165	8	+	light responsiveness
Gbar_D12G002730.1	1303	6	-	anaerobic induction
Gbar_D12G002730.1	1873	6	-	anaerobic induction
Gbar_D12G002730.1	491	6	-	light responsiveness
Gbar_D12G002730.1	581	6	+	light responsiveness
Gbar_D12G002730.1	1602	6	-	light responsiveness
Gbar_D12G002730.1	7	6	-	low-temperature responsiveness
Gbar_D12G002730.1	553	6	+	low-temperature responsiveness
Gbar_D12G002730.1	142	9	-	light responsiveness

Gbar_D12G002730.1	1357	6	+	drought inducibility
Gbar_D12G002730.1	735	5	-	abscisic acid responsiveness
Gbar_D12G002730.1	1168	5	+	abscisic acid responsiveness
Gbar_D12G002730.1	1590	6	-	light responsiveness
Gbar_D12G002730.1	1724	9	+	salicylic acid responsiveness
Gbar_D12G002730.1	1211	9	-	light responsiveness
Gbar_D12G002730.1	44	6	+	light responsiveness
Gbar_D12G002730.1	136	6	+	light responsiveness
Gbar_D12G002730.1	168	6	+	light responsiveness
Gbar_D12G002730.1	350	6	+	light responsiveness
Gbar_D12G002730.1	735	6	+	light responsiveness
Gbar_D12G002730.1	1167	6	-	light responsiveness
Gbar_D12G023100.1	1779	7	-	light responsiveness
Gbar_D12G023100.1	1107	7	-	gibberellin responsiveness
Gbar_D12G023100.1	503	6	-	anaerobic induction
Gbar_D12G023100.1	1068	6	+	anaerobic induction
Gbar_D12G023100.1	1708	6	-	anaerobic induction
Gbar_D12G023100.1	175	6	-	low-temperature responsiveness
Gbar_D12G023100.1	1990	7	+	light responsiveness
Gbar_D12G023100.1	910	9	-	abscisic acid responsiveness
Gbar_D12G023100.1	290	6	-	auxin responsiveness
Gbar_D12G023100.1	1600	6	-	auxin responsiveness
Gbar_D12G023100.1	1561	9	+	salicylic acid responsiveness
Gbar_D12G023100.1	430	6	-	light responsiveness
Gbar_D12G023100.1	654	6	-	light responsiveness
Gbar_D12G023100.1	55	5	+	MeJA responsiveness
Gbar_D12G023100.1	1966	5	-	MeJA responsiveness
Gbar_D12G023100.1	221	6	+	light responsiveness
Gbar_D12G023100.1	307	6	+	light responsiveness
Gbar_D12G023100.1	55	5	-	MeJA responsiveness
Gbar_D12G023100.1	1966	5	+	MeJA responsiveness
Gbar_D13G003400.1	127	9	+	defense and stress responsiveness
Gbar_D13G003400.1	1354	9	+	defense and stress responsiveness
Gbar_D13G003400.1	989	6	+	light responsiveness
Gbar_D13G003400.1	1201	6	-	auxin responsiveness
Gbar_D13G003400.1	1152	9	-	light responsiveness
Gbar_D13G003400.1	781	9	+	salicylic acid responsiveness
Gbar_D13G003400.1	1576	6	-	light responsiveness
Gbar_D13G003400.1	110	8	+	abscisic acid responsiveness
Gbar_D13G003400.1	111	5	+	abscisic acid responsiveness

Gbar_D13G003400.1	1700	10	-	light responsiveness
Gbar_D13G003400.1	108	9	-	light responsiveness
Gbar_D13G003400.1	53	10	-	light responsiveness
Gbar_D13G003400.1	895	7	+	gibberellin responsiveness
Gbar_D13G003400.1	519	6	+	low-temperature responsiveness
Gbar_D13G003400.1	131	6	+	light responsiveness
Gbar_D13G003400.1	490	10	+	light responsiveness
Gbar_D13G003400.1	937	6	-	anaerobic induction
Gbar_D13G003400.1	1314	6	-	anaerobic induction
Gbar_D13G003400.1	1522	6	+	anaerobic induction
Gbar_D13G003400.1	1572	6	-	anaerobic induction
Gbar_D13G003400.1	1867	6	-	anaerobic induction
Gbar_D13G003400.1	110	6	+	light responsiveness
Gbar_D13G003400.1	1727	9	-	light responsiveness
Gbar_D13G009810.1	1018	8	+	light responsiveness
Gbar_D13G009810.1	1472	8	+	light responsiveness
Gbar_D13G009810.1	1808	8	-	light responsiveness
Gbar_D13G009810.1	939	6	+	light responsiveness
Gbar_D13G009810.1	1210	6	-	light responsiveness
Gbar_D13G009810.1	210	6	+	light responsiveness
Gbar_D13G009810.1	439	6	+	light responsiveness
Gbar_D13G009810.1	1919	6	+	light responsiveness
Gbar_D13G009810.1	1946	9	+	salicylic acid responsiveness
Gbar_D13G009810.1	1383	5	+	abscisic acid responsiveness
Gbar_D13G009810.1	964	9	+	light responsiveness
Gbar_D13G009810.1	1979	6	-	light responsiveness
Gbar_D13G009810.1	831	6	-	anaerobic induction
Gbar_D13G009810.1	1382	6	+	light responsiveness
Gbar_D13G016200.1	106	5	-	MeJA responsiveness
Gbar_D13G016200.1	1931	5	-	MeJA responsiveness
Gbar_D13G016200.1	1115	8	+	light responsiveness
Gbar_D13G016200.1	397	8	+	light responsiveness
Gbar_D13G016200.1	1458	6	-	light responsiveness
Gbar_D13G016200.1	1320	6	+	light responsiveness
Gbar_D13G016200.1	106	5	+	MeJA responsiveness
Gbar_D13G016200.1	1931	5	+	MeJA responsiveness
Gbar_D13G016200.1	785	6	+	light responsiveness
Gbar_D13G016200.1	865	6	+	light responsiveness
Gbar_D13G016200.1	1689	6	-	light responsiveness
Gbar_D13G016200.1	1715	9	+	light responsiveness

Gbar_D13G016200.1	1458	6	-	abscisic acid responsiveness
Gbar_D13G016200.1	1459	5	+	abscisic acid responsiveness
Gbar_D13G016200.1	1458	6	-	light responsiveness
Gbar_D13G016200.1	1910	7	-	light responsiveness
Gbar_D13G016200.1	1917	6	-	anaerobic induction
Gbar_D13G016200.1	1400	6	-	light responsiveness

Table S4c. cis elements in Gh RF2 genes

Ghir_A01G022860.1	1006	7	+	light responsiveness
Ghir_A01G022860.1	329	6	+	anaerobic induction
Ghir_A01G022860.1	1038	6	-	anaerobic induction
Ghir_A01G022860.1	1604	6	-	light responsiveness
Ghir_A01G022860.1	1868	6	-	light responsiveness
Ghir_A01G022860.1	67	6	+	light responsiveness
Ghir_A01G022860.1	314	6	+	light responsiveness
Ghir_A01G022860.1	516	6	+	light responsiveness
Ghir_A01G022860.1	586	6	+	light responsiveness
Ghir_A01G022860.1	607	6	+	light responsiveness
Ghir_A01G022860.1	627	6	+	light responsiveness
Ghir_A01G022860.1	769	6	+	light responsiveness
Ghir_A01G022860.1	803	6	+	light responsiveness
Ghir_A01G022860.1	1779	6	-	light responsiveness
Ghir_A01G022860.1	1604	6	-	abscisic acid responsiveness
Ghir_A01G022860.1	1605	5	+	abscisic acid responsiveness
Ghir_A01G022860.1	1604	6	-	light responsiveness
Ghir_A01G022860.1	1122	7	-	auxin responsiveness
Ghir_A01G022860.1	118	9	+	salicylic acid responsiveness
Ghir_A02G001350.1	255	5	-	abscisic acid responsiveness
Ghir_A02G001350.1	1006	5	-	MeJA responsiveness
Ghir_A02G001350.1	348	6	-	light responsiveness
Ghir_A02G001350.1	796	6	+	light responsiveness
Ghir_A02G001350.1	1725	9	+	salicylic acid responsiveness
Ghir_A02G001350.1	255	6	+	light responsiveness
Ghir_A02G001350.1	1006	5	+	MeJA responsiveness
Ghir_A02G001350.1	884	6	-	auxin responsiveness
Ghir_A02G001350.1	335	6	+	light responsiveness
Ghir_A02G001350.1	933	6	+	light responsiveness
Ghir_A02G001350.1	953	6	+	light responsiveness
Ghir_A02G001350.1	1254	6	-	light responsiveness
Ghir_A02G001350.1	1343	6	-	light responsiveness
Ghir_A02G001350.1	1498	6	-	light responsiveness
Ghir_A02G001350.1	1605	6	-	light responsiveness
Ghir_A02G001350.1	836	6	+	low-temperature responsiveness
Ghir_A02G001350.1	255	8	-	light responsiveness
Ghir_A02G006740.1	1959	10	+	light responsiveness
Ghir_A02G006740.1	148	10	+	light responsiveness

Ghir_A02G006740.1	287	10	-	light responsiveness
Ghir_A02G006740.1	358	6	-	light responsiveness
Ghir_A02G006740.1	1466	6	-	light responsiveness
Ghir_A02G006740.1	1483	6	+	light responsiveness
Ghir_A02G006740.1	195	5	+	abscisic acid responsiveness
Ghir_A02G006740.1	359	5	+	abscisic acid responsiveness
Ghir_A02G006740.1	870	5	+	abscisic acid responsiveness
Ghir_A02G006740.1	1467	5	+	abscisic acid responsiveness
Ghir_A02G006740.1	1483	5	-	abscisic acid responsiveness
Ghir_A02G006740.1	868	5	-	MeJA responsiveness
Ghir_A02G006740.1	1706	6	-	light responsiveness
Ghir_A02G006740.1	1449	6	+	light responsiveness
Ghir_A02G006740.1	1549	6	-	light responsiveness
Ghir_A02G006740.1	1917	6	+	light responsiveness
Ghir_A02G006740.1	1937	9	+	defense and stress responsiveness
Ghir_A02G006740.1	307	7	-	light responsiveness
Ghir_A02G006740.1	792	6	-	drought inducibility
Ghir_A02G006740.1	301	6	-	auxin responsiveness
Ghir_A02G006740.1	77	8	+	light responsiveness
Ghir_A02G006740.1	193	9	-	light responsiveness
Ghir_A02G006740.1	194	6	+	light responsiveness
Ghir_A02G006740.1	869	6	-	light responsiveness
Ghir_A02G006740.1	1464	8	+	light responsiveness
Ghir_A02G006740.1	868	5	+	MeJA responsiveness
Ghir_A03G009200.1	849	6	+	light responsiveness
Ghir_A03G009200.1	1697	6	-	light responsiveness
Ghir_A03G009200.1	169	6	+	light responsiveness
Ghir_A03G009200.1	661	6	+	light responsiveness
Ghir_A03G009200.1	772	6	-	light responsiveness
Ghir_A03G009200.1	1226	6	+	light responsiveness
Ghir_A03G009200.1	1896	6	-	light responsiveness
Ghir_A03G009200.1	247	5	-	abscisic acid responsiveness
Ghir_A03G009200.1	1312	5	-	abscisic acid responsiveness
Ghir_A03G009200.1	271	6	-	light responsiveness
Ghir_A03G009200.1	58	13	+	light responsiveness
Ghir_A03G009200.1	1242	7	+	gibberellin responsiveness
Ghir_A03G009200.1	232	7	-	light responsiveness
Ghir_A03G009200.1	518	7	+	light responsiveness
Ghir_A03G009200.1	1003	6	-	anaerobic induction
Ghir_A03G009200.1	1243	10	+	light responsiveness

Ghir_A03G009200.1	356	6	+	light responsiveness
Ghir_A03G009200.1	668	6	+	light responsiveness
Ghir_A03G009200.1	813	6	+	light responsiveness
Ghir_A03G009200.1	647	8	-	light responsiveness
Ghir_A03G009200.1	1311	9	+	light responsiveness
Ghir_A03G009200.1	1953	9.5	-	light responsiveness
Ghir_A03G009200.1	247	6	-	light responsiveness
Ghir_A03G009200.1	1309	9	+	light responsiveness
Ghir_A03G009200.1	1312	6	-	light responsiveness
Ghir_A03G009200.1	346	6	+	drought inducibility
Ghir_A03G009200.1	1982	6	-	drought inducibility
Ghir_A03G009200.1	1055	6	-	auxin responsiveness
Ghir_A03G021100.1	1668	7	+	light responsiveness
Ghir_A03G021100.1	1892	9	-	light responsiveness
Ghir_A03G021100.1	558	7	-	light responsiveness
Ghir_A03G021100.1	1671	7	-	light responsiveness
Ghir_A03G021100.1	34	6	-	light responsiveness
Ghir_A03G021100.1	425	6	+	light responsiveness
Ghir_A03G021100.1	856	6	+	light responsiveness
Ghir_A03G021100.1	595	9	-	salicylic acid responsiveness
Ghir_A03G021100.1	874	9	+	salicylic acid responsiveness
Ghir_A03G021100.1	1711	6	-	anaerobic induction
Ghir_A03G021100.1	1854	6	-	anaerobic induction
Ghir_A03G021100.1	804	10	-	light responsiveness
Ghir_A03G021100.1	850	10	-	light responsiveness
Ghir_A03G021100.1	1918	10	-	light responsiveness
Ghir_A03G021100.1	702	6	-	light responsiveness
Ghir_A03G021100.1	1133	6	+	low-temperature responsiveness
Ghir_A03G021100.1	1088	6	-	light responsiveness
Ghir_A03G021100.1	1944	6	-	light responsiveness
Ghir_A03G021100.1	70	6	-	auxin responsiveness
Ghir_A03G021810.1	29	6	+	light responsiveness
Ghir_A03G021810.1	723	6	+	light responsiveness
Ghir_A03G021810.1	1031	6	-	light responsiveness
Ghir_A03G021810.1	1216	6	-	light responsiveness
Ghir_A03G021810.1	1460	10	+	light responsiveness
Ghir_A03G021810.1	1588	6	+	light responsiveness
Ghir_A03G021810.1	379	6	+	drought inducibility
Ghir_A03G021810.1	147	5	-	MeJA responsiveness
Ghir_A03G021810.1	241	5	-	MeJA responsiveness

Ghir_A03G021810.1	692	6	+	anaerobic induction
Ghir_A03G021810.1	732	14	+	light responsiveness
Ghir_A03G021810.1	1728	8	-	light responsiveness
Ghir_A03G021810.1	1905	8	-	light responsiveness
Ghir_A03G021810.1	351	7	+	light responsiveness
Ghir_A03G021810.1	1964	6	+	light responsiveness
Ghir_A03G021810.1	1244	9	-	defense and stress responsiveness
Ghir_A03G021810.1	1589	5	+	abscisic acid responsiveness
Ghir_A03G021810.1	147	5	+	MeJA responsiveness
Ghir_A03G021810.1	241	5	+	MeJA responsiveness
Ghir_A05G027550.1	609	6	+	anaerobic induction
Ghir_A05G027550.1	1957	5	-	MeJA responsiveness
Ghir_A05G027550.1	37	6	+	drought inducibility
Ghir_A05G027550.1	1388	6	+	drought inducibility
Ghir_A05G027550.1	159	6	-	light responsiveness
Ghir_A05G027550.1	291	6	+	light responsiveness
Ghir_A05G027550.1	1284	6	-	light responsiveness
Ghir_A05G027550.1	1308	6	-	light responsiveness
Ghir_A05G027550.1	1954	8	-	auxin responsiveness
Ghir_A05G027550.1	1957	5	+	MeJA responsiveness
Ghir_A05G027550.1	160	5	+	abscisic acid responsiveness
Ghir_A05G027550.1	986	10	-	light responsiveness
Ghir_A05G027550.1	1795	6	+	light responsiveness
Ghir_A05G027550.1	91	9	-	salicylic acid responsiveness
Ghir_A05G028410.1	1470	8	+	light responsiveness
Ghir_A05G028410.1	1416	9	-	circadian control
Ghir_A05G028410.1	18	10	+	light responsiveness
Ghir_A05G028410.1	1484	7	-	gibberellin responsiveness
Ghir_A05G028410.1	754	5	-	abscisic acid responsiveness
Ghir_A05G028410.1	247	5	-	MeJA responsiveness
Ghir_A05G028410.1	756	5	+	MeJA responsiveness
Ghir_A05G028410.1	1224	9	+	defense and stress responsiveness
Ghir_A05G028410.1	1899	6	+	low-temperature responsiveness
Ghir_A05G028410.1	298	8	+	light responsiveness
Ghir_A05G028410.1	125	6	+	light responsiveness
Ghir_A05G028410.1	793	6	+	light responsiveness
Ghir_A05G028410.1	925	6	+	light responsiveness
Ghir_A05G028410.1	1022	6	-	light responsiveness
Ghir_A05G028410.1	1037	6	-	light responsiveness
Ghir_A05G028410.1	1092	6	-	light responsiveness

Ghir_A05G028410.1	1326	9.5	-	light responsiveness
Ghir_A05G028410.1	754	6	+	light responsiveness
Ghir_A05G028410.1	209	6	-	anaerobic induction
Ghir_A05G028410.1	1528	6	-	anaerobic induction
Ghir_A05G028410.1	1764	6	+	anaerobic induction
Ghir_A05G028410.1	247	5	+	MeJA responsiveness
Ghir_A05G028410.1	756	5	-	MeJA responsiveness
Ghir_A05G041580.1	662	13	-	light responsiveness
Ghir_A05G041580.1	1375	13	-	light responsiveness
Ghir_A05G041580.1	1487	8	+	light responsiveness
Ghir_A05G041580.1	1687	8	+	light responsiveness
Ghir_A05G041580.1	1274	9	+	defense and stress responsiveness
Ghir_A05G041580.1	1278	6	+	light responsiveness
Ghir_A05G041580.1	1126	6	-	light responsiveness
Ghir_A05G041580.1	1001	5	+	MeJA responsiveness
Ghir_A05G041580.1	919	7	-	abscisic acid responsiveness
Ghir_A05G041580.1	1550	8	-	light responsiveness
Ghir_A05G041580.1	155	6	+	light responsiveness
Ghir_A05G041580.1	606	6	+	light responsiveness
Ghir_A05G041580.1	1502	6	-	light responsiveness
Ghir_A05G041580.1	1887	6	-	auxin responsiveness
Ghir_A05G041580.1	1001	5	-	MeJA responsiveness
Ghir_A05G041580.1	1232	6	+	anaerobic induction
Ghir_A05G041580.1	1588	6	-	anaerobic induction
Ghir_A06G019360.1	964	6	-	anaerobic induction
Ghir_A06G019360.1	1209	6	-	anaerobic induction
Ghir_A06G019360.1	1090	6	-	auxin responsiveness
Ghir_A06G019360.1	1511	6	+	auxin responsiveness
Ghir_A06G019360.1	49	6	+	drought inducibility
Ghir_A06G019360.1	622	9	+	light responsiveness
Ghir_A06G019360.1	763	9	-	light responsiveness
Ghir_A06G019360.1	643	6	+	light responsiveness
Ghir_A06G019360.1	1236	6	-	light responsiveness
Ghir_A06G019360.1	1249	6	-	light responsiveness
Ghir_A06G019360.1	1339	6	-	light responsiveness
Ghir_A06G019360.1	1785	6	-	light responsiveness
Ghir_A06G019360.1	1985	7	-	gibberellin responsiveness
Ghir_A06G019360.1	1805	7	-	light responsiveness
Ghir_A06G019360.1	1806	6	-	light responsiveness
Ghir_A06G019360.1	1289	9	+	light responsiveness

Ghir_A06G019360.1	975	6	-	light responsiveness
Ghir_A06G019360.1	242	7	-	auxin responsiveness
Ghir_A09G022110.1	768	5	-	MeJA responsiveness
Ghir_A09G022110.1	1069	5	+	MeJA responsiveness
Ghir_A09G022110.1	1170	6	-	light responsiveness
Ghir_A09G022110.1	92	6	+	anoxic specific inducibility
Ghir_A09G022110.1	584	6	+	light responsiveness
Ghir_A09G022110.1	742	6	+	light responsiveness
Ghir_A09G022110.1	1378	6	-	light responsiveness
Ghir_A09G022110.1	3	5	+	abscisic acid responsiveness
Ghir_A09G022110.1	585	5	+	abscisic acid responsiveness
Ghir_A09G022110.1	742	5	-	abscisic acid responsiveness
Ghir_A09G022110.1	1371	5	+	abscisic acid responsiveness
Ghir_A09G022110.1	1378	5	-	abscisic acid responsiveness
Ghir_A09G022110.1	1454	7	-	gibberellin responsiveness
Ghir_A09G022110.1	878	6	+	anaerobic induction
Ghir_A09G022110.1	1907	6	-	anaerobic induction
Ghir_A09G022110.1	971	8	+	light responsiveness
Ghir_A09G022110.1	832	6	-	low-temperature responsiveness
Ghir_A09G022110.1	1969	6	-	low-temperature responsiveness
Ghir_A09G022110.1	768	5	+	MeJA responsiveness
Ghir_A09G022110.1	1069	5	-	MeJA responsiveness
Ghir_A09G022110.1	1049	6	+	light responsiveness
Ghir_A09G022110.1	1777	9	+	salicylic acid responsiveness
Ghir_A09G022110.1	2	6	-	light responsiveness
Ghir_A09G022110.1	1370	6	-	light responsiveness
Ghir_A09G022110.1	1147	13	-	light responsiveness
Ghir_A10G014150.1	243	6	+	light responsiveness
Ghir_A10G014150.1	553	6	+	light responsiveness
Ghir_A10G014150.1	1051	6	-	light responsiveness
Ghir_A10G014150.1	1539	9	-	wound responsiveness
Ghir_A10G014150.1	1780	9.5	-	light responsiveness
Ghir_A10G014150.1	1283	9	+	light responsiveness
Ghir_A10G014150.1	1348	9	+	salicylic acid responsiveness
Ghir_A10G014150.1	1659	9	-	salicylic acid responsiveness
Ghir_A10G014150.1	849	6	+	light responsiveness
Ghir_A10G014150.1	1032	6	+	light responsiveness
Ghir_A10G014150.1	1296	10	+	light responsiveness
Ghir_A10G014150.1	269	6	-	anaerobic induction
Ghir_A10G014150.1	372	6	+	anaerobic induction

Ghir_A10G014150.1	642	6	+	anaerobic induction
Ghir_A10G014150.1	1792	6	+	anaerobic induction
Ghir_A10G018390.1	1739	8	+	light responsiveness
Ghir_A10G018390.1	484	6	+	light responsiveness
Ghir_A10G018390.1	814	6	+	light responsiveness
Ghir_A10G018390.1	839	6	+	light responsiveness
Ghir_A10G018390.1	1402	6	-	light responsiveness
Ghir_A10G018390.1	1699	6	-	light responsiveness
Ghir_A10G018390.1	584	8	+	light responsiveness
Ghir_A10G018390.1	1757	6	-	light responsiveness
Ghir_A10G018390.1	1855	6	-	auxin responsiveness
Ghir_A10G018390.1	1314	6	+	light responsiveness
Ghir_A10G018390.1	1755	9	+	light responsiveness
Ghir_A10G018390.1	822	7	-	light responsiveness
Ghir_A10G018390.1	731	9	+	abscisic acid responsiveness
Ghir_A10G018390.1	1755	8	-	abscisic acid responsiveness
Ghir_A10G018390.1	1757	5	-	abscisic acid responsiveness
Ghir_A10G018390.1	747	9	-	salicylic acid responsiveness
Ghir_A11G031340.1	1155	5	-	MeJA responsiveness
Ghir_A11G031340.1	227	5	-	abscisic acid responsiveness
Ghir_A11G031340.1	926	5	+	abscisic acid responsiveness
Ghir_A11G031340.1	1036	6	-	abscisic acid responsiveness
Ghir_A11G031340.1	1037	5	+	abscisic acid responsiveness
Ghir_A11G031340.1	1222	5	+	abscisic acid responsiveness
Ghir_A11G031340.1	1452	7	+	light responsiveness
Ghir_A11G031340.1	1725	7	+	light responsiveness
Ghir_A11G031340.1	1960	7	-	light responsiveness
Ghir_A11G031340.1	1961	6	-	light responsiveness
Ghir_A11G031340.1	764	9	+	salicylic acid responsiveness
Ghir_A11G031340.1	260	13	-	light responsiveness
Ghir_A11G031340.1	227	6	+	light responsiveness
Ghir_A11G031340.1	1036	6	-	light responsiveness
Ghir_A11G031340.1	943	6	-	anaerobic induction
Ghir_A11G031340.1	1155	5	+	MeJA responsiveness
Ghir_A11G031340.1	152	9	-	light responsiveness
Ghir_A11G031340.1	925	6	+	light responsiveness
Ghir_A11G031340.1	1035	8	-	light responsiveness
Ghir_A11G031340.1	1036	6	-	light responsiveness
Ghir_A11G031340.1	1221	6	+	light responsiveness
Ghir_A11G031340.1	1219	9	-	light responsiveness

Ghir_A11G031340.1	520	6	+	light responsiveness
Ghir_A11G031340.1	653	6	+	light responsiveness
Ghir_A11G031340.1	1764	6	-	light responsiveness
Ghir_A11G031340.1	1768	6	-	light responsiveness
Ghir_A12G002680.1	915	7	-	light responsiveness
Ghir_A12G002680.1	338	5	-	MeJA responsiveness
Ghir_A12G002680.1	1298	6	-	anaerobic induction
Ghir_A12G002680.1	1871	6	-	anaerobic induction
Ghir_A12G002680.1	1206	9	-	light responsiveness
Ghir_A12G002680.1	1160	8	+	light responsiveness
Ghir_A12G002680.1	41	6	+	light responsiveness
Ghir_A12G002680.1	62	6	+	light responsiveness
Ghir_A12G002680.1	151	6	+	light responsiveness
Ghir_A12G002680.1	162	6	+	light responsiveness
Ghir_A12G002680.1	378	6	+	light responsiveness
Ghir_A12G002680.1	975	6	-	drought inducibility
Ghir_A12G002680.1	1586	6	-	light responsiveness
Ghir_A12G002680.1	533	6	-	light responsiveness
Ghir_A12G002680.1	623	6	+	light responsiveness
Ghir_A12G002680.1	1598	6	-	light responsiveness
Ghir_A12G002680.1	338	5	+	MeJA responsiveness
Ghir_A12G002680.1	335	8	-	auxin responsiveness
Ghir_A12G002680.1	1688	7	+	gibberellin responsiveness
Ghir_A12G002680.1	1163	5	+	abscisic acid responsiveness
Ghir_A12G002680.1	1162	6	-	light responsiveness
Ghir_A13G003420.1	1304	8	-	light responsiveness
Ghir_A13G003420.1	1201	6	-	auxin responsiveness
Ghir_A13G003420.1	1484	6	-	light responsiveness
Ghir_A13G003420.1	1726	9	-	light responsiveness
Ghir_A13G003420.1	988	6	+	light responsiveness
Ghir_A13G003420.1	518	6	+	low-temperature responsiveness
Ghir_A13G003420.1	937	6	-	anaerobic induction
Ghir_A13G003420.1	1521	6	+	anaerobic induction
Ghir_A13G003420.1	1571	6	-	anaerobic induction
Ghir_A13G003420.1	1866	6	-	anaerobic induction
Ghir_A13G003420.1	121	5	-	MeJA responsiveness
Ghir_A13G003420.1	1186	7	-	light responsiveness
Ghir_A13G003420.1	844	9	+	wound responsiveness
Ghir_A13G003420.1	121	5	+	MeJA responsiveness
Ghir_A13G003420.1	1353	9	+	defense and stress responsiveness

Ghir_A13G003420.1	1575	6	-	light responsiveness
Ghir_A13G009010.1	1946	9	+	salicylic acid responsiveness
Ghir_A13G009010.1	1811	8	-	light responsiveness
Ghir_A13G009010.1	460	7	-	auxin responsiveness
Ghir_A13G009010.1	132	5	-	MeJA responsiveness
Ghir_A13G009010.1	1381	5	+	abscisic acid responsiveness
Ghir_A13G009010.1	346	7	-	gibberellin responsiveness
Ghir_A13G009010.1	1445	10	+	light responsiveness
Ghir_A13G009010.1	136	7	+	light responsiveness
Ghir_A13G009010.1	1919	6	+	light responsiveness
Ghir_A13G009010.1	1979	6	-	light responsiveness
Ghir_A13G009010.1	1380	6	+	light responsiveness
Ghir_A13G009010.1	968	9	+	light responsiveness
Ghir_A13G009010.1	14	6	+	light responsiveness
Ghir_A13G009010.1	27	6	+	light responsiveness
Ghir_A13G009010.1	271	6	+	light responsiveness
Ghir_A13G009010.1	291	6	+	light responsiveness
Ghir_A13G009010.1	794	6	+	light responsiveness
Ghir_A13G009010.1	871	6	+	light responsiveness
Ghir_A13G009010.1	35	6	-	anaerobic induction
Ghir_A13G009010.1	652	6	-	anaerobic induction
Ghir_A13G009010.1	1201	6	+	anaerobic induction
Ghir_A13G009010.1	1474	8	+	light responsiveness
Ghir_A13G009010.1	132	5	+	MeJA responsiveness
Ghir_A13G015530.1	1097	6	-	light responsiveness
Ghir_A13G015530.1	1465	6	-	light responsiveness
Ghir_A13G015530.1	403	6	+	low-temperature responsiveness
Ghir_A13G015530.1	251	6	+	light responsiveness
Ghir_A13G015530.1	893	6	+	light responsiveness
Ghir_A13G015530.1	1699	6	-	light responsiveness
Ghir_A13G015530.1	752	7	-	light responsiveness
Ghir_A13G015530.1	1931	5	+	MeJA responsiveness
Ghir_A13G015530.1	1859	6	-	anaerobic induction
Ghir_A13G015530.1	1917	6	-	anaerobic induction
Ghir_A13G015530.1	1097	6	-	light responsiveness
Ghir_A13G015530.1	1465	6	-	light responsiveness
Ghir_A13G015530.1	1140	8	+	light responsiveness
Ghir_A13G015530.1	1342	6	+	light responsiveness
Ghir_A13G015530.1	1931	5	-	MeJA responsiveness
Ghir_A13G015530.1	1097	6	-	abscisic acid responsiveness

Ghir_A13G015530.1	1098	5	+	abscisic acid responsiveness
Ghir_A13G015530.1	1465	6	-	abscisic acid responsiveness
Ghir_A13G015530.1	1466	5	+	abscisic acid responsiveness
Ghir_D01G005170.1	148	6	+	light responsiveness
Ghir_D01G005170.1	282	6	+	light responsiveness
Ghir_D01G005170.1	297	6	+	light responsiveness
Ghir_D01G005170.1	378	6	+	light responsiveness
Ghir_D01G005170.1	582	6	+	light responsiveness
Ghir_D01G005170.1	784	6	+	light responsiveness
Ghir_D01G005170.1	826	6	+	light responsiveness
Ghir_D01G005170.1	1606	6	-	light responsiveness
Ghir_D01G005170.1	1868	6	-	light responsiveness
Ghir_D01G005170.1	311	6	+	auxin responsiveness
Ghir_D01G005170.1	656	13	+	light responsiveness
Ghir_D01G005170.1	130	5	+	MeJA responsiveness
Ghir_D01G005170.1	1014	7	+	light responsiveness
Ghir_D01G005170.1	393	6	+	anaerobic induction
Ghir_D01G005170.1	1046	6	-	anaerobic induction
Ghir_D01G005170.1	1606	6	-	light responsiveness
Ghir_D01G005170.1	212	6	-	light responsiveness
Ghir_D01G005170.1	448	6	+	light responsiveness
Ghir_D01G005170.1	8	7	+	light responsiveness
Ghir_D01G005170.1	1606	6	-	abscisic acid responsiveness
Ghir_D01G005170.1	1607	5	+	abscisic acid responsiveness
Ghir_D01G005170.1	1126	7	-	gibberellin responsiveness
Ghir_D01G005170.1	130	5	-	MeJA responsiveness
Ghir_D02G001430.1	24	5	-	MeJA responsiveness
Ghir_D02G001430.1	1050	5	+	MeJA responsiveness
Ghir_D02G001430.1	255	6	-	auxin responsiveness
Ghir_D02G001430.1	401	6	+	light responsiveness
Ghir_D02G001430.1	14	6	+	light responsiveness
Ghir_D02G001430.1	55	6	+	light responsiveness
Ghir_D02G001430.1	80	6	+	light responsiveness
Ghir_D02G001430.1	1003	6	-	light responsiveness
Ghir_D02G001430.1	1023	6	-	light responsiveness
Ghir_D02G001430.1	1513	6	-	light responsiveness
Ghir_D02G001430.1	1620	6	-	light responsiveness
Ghir_D02G001430.1	4	8	+	light responsiveness
Ghir_D02G001430.1	1386	8	+	light responsiveness
Ghir_D02G001430.1	907	6	+	low-temperature responsiveness

Ghir_D02G001430.1	24	5	+	MeJA responsiveness
Ghir_D02G001430.1	1050	5	-	MeJA responsiveness
Ghir_D02G001430.1	401	6	+	abscisic acid responsiveness
Ghir_D02G001430.1	402	5	+	abscisic acid responsiveness
Ghir_D02G001430.1	586	10	+	light responsiveness
Ghir_D02G001430.1	89	6	-	light responsiveness
Ghir_D02G001430.1	570	6	+	light responsiveness
Ghir_D02G001430.1	874	6	+	light responsiveness
Ghir_D02G001430.1	1346	6	-	light responsiveness
Ghir_D02G001430.1	1741	9	+	salicylic acid responsiveness
Ghir_D02G001430.1	1928	8	-	light responsiveness
Ghir_D02G001430.1	401	6	+	light responsiveness
Ghir_D02G007160.1	279	6	+	abscisic acid responsiveness
Ghir_D02G007160.1	280	5	+	abscisic acid responsiveness
Ghir_D02G007160.1	861	5	+	abscisic acid responsiveness
Ghir_D02G007160.1	1590	7	-	gibberellin responsiveness
Ghir_D02G007160.1	859	5	-	MeJA responsiveness
Ghir_D02G007160.1	1711	6	-	light responsiveness
Ghir_D02G007160.1	1452	6	+	light responsiveness
Ghir_D02G007160.1	1917	6	+	light responsiveness
Ghir_D02G007160.1	1937	9	+	defense and stress responsiveness
Ghir_D02G007160.1	1095	10	-	light responsiveness
Ghir_D02G007160.1	1630	9	+	light responsiveness
Ghir_D02G007160.1	1959	10	+	light responsiveness
Ghir_D02G007160.1	279	6	+	light responsiveness
Ghir_D02G007160.1	1594	9	-	wound responsiveness
Ghir_D02G007160.1	481	13	+	light responsiveness
Ghir_D02G007160.1	859	5	+	MeJA responsiveness
Ghir_D02G007160.1	236	6	+	drought inducibility
Ghir_D02G007160.1	783	6	-	drought inducibility
Ghir_D02G007160.1	290	6	-	auxin responsiveness
Ghir_D02G007160.1	508	6	+	light responsiveness
Ghir_D02G007160.1	555	6	+	light responsiveness
Ghir_D02G007160.1	279	6	+	light responsiveness
Ghir_D02G007160.1	860	6	-	light responsiveness
Ghir_D02G007160.1	743	10	-	light responsiveness
Ghir_D02G010000.1	1269	7	+	gibberellin responsiveness
Ghir_D02G010000.1	1938	8	-	light responsiveness
Ghir_D02G010000.1	392	6	-	light responsiveness
Ghir_D02G010000.1	243	5	-	MeJA responsiveness

Ghir_D02G010000.1	243	8	+	auxin responsiveness
Ghir_D02G010000.1	393	5	+	abscisic acid responsiveness
Ghir_D02G010000.1	1253	6	+	light responsiveness
Ghir_D02G010000.1	1897	6	-	light responsiveness
Ghir_D02G010000.1	821	6	+	light responsiveness
Ghir_D02G010000.1	1699	6	-	light responsiveness
Ghir_D02G010000.1	1495	6	+	drought inducibility
Ghir_D02G010000.1	1983	6	-	drought inducibility
Ghir_D02G010000.1	1954	9.5	-	light responsiveness
Ghir_D02G010000.1	633	8	-	light responsiveness
Ghir_D02G010000.1	1340	6	+	anaerobic induction
Ghir_D02G010000.1	243	5	+	MeJA responsiveness
Ghir_D02G010000.1	229	7	-	light responsiveness
Ghir_D02G022550.1	578	9	-	salicylic acid responsiveness
Ghir_D02G022550.1	862	9	+	salicylic acid responsiveness
Ghir_D02G022550.1	844	6	+	light responsiveness
Ghir_D02G022550.1	13	6	-	light responsiveness
Ghir_D02G022550.1	538	7	-	light responsiveness
Ghir_D02G022550.1	1671	7	-	light responsiveness
Ghir_D02G022550.1	1668	7	+	light responsiveness
Ghir_D02G022550.1	1600	7	+	abscisic acid responsiveness
Ghir_D02G022550.1	153	5	+	MeJA responsiveness
Ghir_D02G022550.1	1944	6	-	light responsiveness
Ghir_D02G022550.1	1123	6	+	low-temperature responsiveness
Ghir_D02G022550.1	838	10	-	light responsiveness
Ghir_D02G022550.1	48	6	-	auxin responsiveness
Ghir_D02G022550.1	153	5	-	MeJA responsiveness
Ghir_D02G022550.1	1536	6	-	anaerobic induction
Ghir_D02G022550.1	1711	6	-	anaerobic induction
Ghir_D02G022550.1	1854	6	-	anaerobic induction
Ghir_D02G023300.1	1268	6	-	light responsiveness
Ghir_D02G023300.1	723	13	+	light responsiveness
Ghir_D02G023300.1	1722	8	-	light responsiveness
Ghir_D02G023300.1	1899	8	-	light responsiveness
Ghir_D02G023300.1	343	7	+	light responsiveness
Ghir_D02G023300.1	1961	6	+	light responsiveness
Ghir_D02G023300.1	1070	9	+	light responsiveness
Ghir_D02G023300.1	184	7	-	gibberellin responsiveness
Ghir_D02G023300.1	240	7	-	gibberellin responsiveness
Ghir_D02G023300.1	976	5	+	abscisic acid responsiveness

Ghir_D02G023300.1	1269	5	+	abscisic acid responsiveness
Ghir_D02G023300.1	1587	5	+	abscisic acid responsiveness
Ghir_D02G023300.1	232	5	+	MeJA responsiveness
Ghir_D02G023300.1	974	5	-	MeJA responsiveness
Ghir_D02G023300.1	992	5	+	MeJA responsiveness
Ghir_D02G023300.1	714	6	+	light responsiveness
Ghir_D02G023300.1	724	6	+	light responsiveness
Ghir_D02G023300.1	1233	6	-	light responsiveness
Ghir_D02G023300.1	1296	10	+	light responsiveness
Ghir_D02G023300.1	975	6	-	light responsiveness
Ghir_D02G023300.1	1586	6	+	light responsiveness
Ghir_D02G023300.1	920	8	-	light responsiveness
Ghir_D02G023300.1	232	5	-	MeJA responsiveness
Ghir_D02G023300.1	974	5	+	MeJA responsiveness
Ghir_D02G023300.1	992	5	-	MeJA responsiveness
Ghir_D02G023300.1	286	6	-	anaerobic induction
Ghir_D02G023300.1	587	6	+	anaerobic induction
Ghir_D02G023300.1	683	6	+	anaerobic induction
Ghir_D02G023300.1	1045	6	+	anaerobic induction
Ghir_D04G001370.1	1702	8	+	light responsiveness
Ghir_D04G001370.1	1115	6	-	light responsiveness
Ghir_D04G001370.1	61	9	+	light responsiveness
Ghir_D04G001370.1	1321	6	+	light responsiveness
Ghir_D04G001370.1	1317	9	+	defense and stress responsiveness
Ghir_D04G001370.1	735	7	-	gibberellin responsiveness
Ghir_D04G001370.1	1450	5	+	abscisic acid responsiveness
Ghir_D04G001370.1	995	5	+	MeJA responsiveness
Ghir_D04G001370.1	612	6	+	light responsiveness
Ghir_D04G001370.1	1617	6	-	light responsiveness
Ghir_D04G001370.1	1449	6	+	light responsiveness
Ghir_D04G001370.1	1138	6	-	auxin responsiveness
Ghir_D04G001370.1	1886	6	-	auxin responsiveness
Ghir_D04G001370.1	931	6	-	drought inducibility
Ghir_D04G001370.1	195	8	+	light responsiveness
Ghir_D04G001370.1	995	5	-	MeJA responsiveness
Ghir_D04G001370.1	322	8.5	+	cell cycle regulation
Ghir_D04G017590.1	1036	6	+	auxin responsiveness
Ghir_D04G017590.1	803	6	+	drought inducibility
Ghir_D04G017590.1	1542	8	-	light responsiveness
Ghir_D04G017590.1	20	6	+	low-temperature responsiveness

Ghir_D04G017590.1	922	9	+	light responsiveness
Ghir_D04G017590.1	1797	6	-	anaerobic induction
Ghir_D04G017590.1	63	9	+	cell cycle regulation
Ghir_D04G017590.1	1523	8	-	light responsiveness
Ghir_D04G017590.1	1127	9	+	circadian control
Ghir_D04G017590.1	1308	9	+	salicylic acid responsiveness
Ghir_D04G017590.1	754	7	-	gibberellin responsiveness
Ghir_D04G017590.1	1137	6	+	light responsiveness
Ghir_D04G017590.1	69	6	+	light responsiveness
Ghir_D04G017590.1	1644	10	-	light responsiveness
Ghir_D05G027570.1	705	6	+	anaerobic induction
Ghir_D05G027570.1	1969	6	+	low-temperature responsiveness
Ghir_D05G027570.1	478	9	-	light responsiveness
Ghir_D05G027570.1	1202	6	+	drought inducibility
Ghir_D05G027570.1	533	6	+	light responsiveness
Ghir_D05G027570.1	1911	6	+	light responsiveness
Ghir_D05G027570.1	86	6	+	light responsiveness
Ghir_D05G027570.1	460	5	+	MeJA responsiveness
Ghir_D05G027570.1	1780	5	+	MeJA responsiveness
Ghir_D05G027570.1	1957	5	+	MeJA responsiveness
Ghir_D05G027570.1	1777	8	-	auxin responsiveness
Ghir_D05G027570.1	1764	13	-	light responsiveness
Ghir_D05G027570.1	269	9	-	salicylic acid responsiveness
Ghir_D05G027570.1	460	5	-	MeJA responsiveness
Ghir_D05G027570.1	1780	5	-	MeJA responsiveness
Ghir_D05G027570.1	1957	5	-	MeJA responsiveness
Ghir_D05G027570.1	41	7	+	light responsiveness
Ghir_D05G027570.1	436	6	+	light responsiveness
Ghir_D05G027570.1	1095	6	-	light responsiveness
Ghir_D05G027570.1	1120	6	-	light responsiveness
Ghir_D05G027570.1	623	6	+	light responsiveness
Ghir_D05G027570.1	1847	6	+	light responsiveness
Ghir_D05G027570.1	1556	6	+	auxin responsiveness
Ghir_D05G027570.1	1267	9	-	light responsiveness
Ghir_D05G027570.1	1539	10	+	light responsiveness
Ghir_D05G027570.1	797	10	-	light responsiveness
Ghir_D05G027570.1	802	10	-	light responsiveness
Ghir_D05G027570.1	1651	8	+	light responsiveness
Ghir_D05G027570.1	1848	5	+	abscisic acid responsiveness
Ghir_D05G028420.1	546	6	-	anaerobic induction

Ghir_D05G028420.1	1531	6	+	anaerobic induction
Ghir_D05G028420.1	1544	6	+	anaerobic induction
Ghir_D05G028420.1	1583	6	-	anaerobic induction
Ghir_D05G028420.1	1780	6	+	anaerobic induction
Ghir_D05G028420.1	87	5	+	MeJA responsiveness
Ghir_D05G028420.1	762	8	+	light responsiveness
Ghir_D05G028420.1	1914	6	+	low-temperature responsiveness
Ghir_D05G028420.1	1747	8	+	light responsiveness
Ghir_D05G028420.1	211	6	+	light responsiveness
Ghir_D05G028420.1	733	6	+	light responsiveness
Ghir_D05G028420.1	776	6	+	light responsiveness
Ghir_D05G028420.1	1022	6	-	light responsiveness
Ghir_D05G028420.1	1404	6	-	light responsiveness
Ghir_D05G028420.1	740	9	-	light responsiveness
Ghir_D05G028420.1	1273	9.5	-	light responsiveness
Ghir_D05G028420.1	1970	5	-	abscisic acid responsiveness
Ghir_D05G028420.1	87	5	-	MeJA responsiveness
Ghir_D05G028420.1	1869	9	+	salicylic acid responsiveness
Ghir_D05G028420.1	1970	6	+	light responsiveness
Ghir_D06G020520.1	1347	6	-	anaerobic induction
Ghir_D06G020520.1	1677	6	-	anaerobic induction
Ghir_D06G020520.1	1451	6	+	auxin responsiveness
Ghir_D06G020520.1	608	6	+	light responsiveness
Ghir_D06G020520.1	653	6	+	light responsiveness
Ghir_D06G020520.1	676	6	+	light responsiveness
Ghir_D06G020520.1	680	6	+	light responsiveness
Ghir_D06G020520.1	988	6	+	light responsiveness
Ghir_D06G020520.1	1277	6	-	light responsiveness
Ghir_D06G020520.1	1608	6	-	light responsiveness
Ghir_D06G020520.1	1101	6	-	low-temperature responsiveness
Ghir_D06G020520.1	1985	7	-	gibberellin responsiveness
Ghir_D06G020520.1	350	6	+	light responsiveness
Ghir_D06G020520.1	1086	6	+	light responsiveness
Ghir_D06G020520.1	913	6	-	light responsiveness
Ghir_D06G020520.1	244	9	+	light responsiveness
Ghir_D06G020520.1	975	10	-	light responsiveness
Ghir_D09G021400.1	1582	7	+	light responsiveness
Ghir_D09G021400.1	23	5	+	abscisic acid responsiveness
Ghir_D09G021400.1	175	6	+	abscisic acid responsiveness
Ghir_D09G021400.1	176	5	+	abscisic acid responsiveness

Ghir_D09G021400.1	716	7	+	abscisic acid responsiveness
Ghir_D09G021400.1	887	9	-	abscisic acid responsiveness
Ghir_D09G021400.1	889	6	+	abscisic acid responsiveness
Ghir_D09G021400.1	890	5	+	abscisic acid responsiveness
Ghir_D09G021400.1	1165	13	-	light responsiveness
Ghir_D09G021400.1	22	6	-	light responsiveness
Ghir_D09G021400.1	175	6	+	light responsiveness
Ghir_D09G021400.1	889	6	+	light responsiveness
Ghir_D09G021400.1	1774	9	+	salicylic acid responsiveness
Ghir_D09G021400.1	1907	6	-	anaerobic induction
Ghir_D09G021400.1	1311	9	-	cell cycle regulation
Ghir_D09G021400.1	20	8	+	light responsiveness
Ghir_D09G021400.1	175	6	+	light responsiveness
Ghir_D09G021400.1	790	9	+	light responsiveness
Ghir_D09G021400.1	889	6	+	light responsiveness
Ghir_D09G021400.1	533	6	+	anoxic specific inducibility
Ghir_D09G021400.1	586	9	+	light responsiveness
Ghir_D09G021400.1	1188	6	-	light responsiveness
Ghir_D09G021400.1	853	6	-	low-temperature responsiveness
Ghir_D09G021400.1	1969	6	-	low-temperature responsiveness
Ghir_D09G021400.1	1297	6	+	auxin responsiveness
Ghir_D09G021400.1	443	6	+	drought inducibility
Ghir_D09G021400.1	751	6	+	drought inducibility
Ghir_D09G021400.1	1455	6	+	drought inducibility
Ghir_D10G013410.1	805	6	-	auxin responsiveness
Ghir_D10G013410.1	1523	6	+	drought inducibility
Ghir_D10G013410.1	210	6	+	light responsiveness
Ghir_D10G013410.1	1011	6	-	light responsiveness
Ghir_D10G013410.1	1485	6	+	low-temperature responsiveness
Ghir_D10G013410.1	1290	9	+	light responsiveness
Ghir_D10G013410.1	112	6	+	anaerobic induction
Ghir_D10G013410.1	247	6	+	anaerobic induction
Ghir_D10G013410.1	362	6	+	anaerobic induction
Ghir_D10G013410.1	671	6	+	anaerobic induction
Ghir_D10G013410.1	971	6	+	anaerobic induction
Ghir_D10G013410.1	299	5	+	MeJA responsiveness
Ghir_D10G013410.1	1663	9	-	salicylic acid responsiveness
Ghir_D10G013410.1	793	6	+	light responsiveness
Ghir_D10G013410.1	1543	9	-	wound responsiveness
Ghir_D10G013410.1	793	5	-	abscisic acid responsiveness

Ghir_D10G013410.1	299	5	-	MeJA responsiveness
Ghir_D10G013410.1	797	6	-	light responsiveness
Ghir_D10G019890.1	1756	9	+	light responsiveness
Ghir_D10G019890.1	560	6	+	light responsiveness
Ghir_D10G019890.1	1225	6	+	light responsiveness
Ghir_D10G019890.1	1438	6	-	light responsiveness
Ghir_D10G019890.1	436	7	-	light responsiveness
Ghir_D10G019890.1	709	9	+	abscisic acid responsiveness
Ghir_D10G019890.1	1756	8	-	abscisic acid responsiveness
Ghir_D10G019890.1	1758	5	-	abscisic acid responsiveness
Ghir_D10G019890.1	1196	5	-	MeJA responsiveness
Ghir_D10G019890.1	725	9	-	salicylic acid responsiveness
Ghir_D10G019890.1	1196	5	+	MeJA responsiveness
Ghir_D10G019890.1	1740	8	+	light responsiveness
Ghir_D10G019890.1	264	6	+	anaerobic induction
Ghir_D10G019890.1	1842	8	+	light responsiveness
Ghir_D10G019890.1	776	6	+	light responsiveness
Ghir_D10G019890.1	804	6	+	light responsiveness
Ghir_D10G019890.1	1081	6	-	light responsiveness
Ghir_D10G019890.1	1313	6	-	light responsiveness
Ghir_D10G019890.1	1600	6	-	light responsiveness
Ghir_D10G019890.1	1700	6	-	light responsiveness
Ghir_D10G019890.1	1758	6	-	light responsiveness
Ghir_D10G019890.1	1855	6	-	auxin responsiveness
Ghir_D12G002680.1	1590	6	-	light responsiveness
Ghir_D12G002680.1	153	9	-	light responsiveness
Ghir_D12G002680.1	501	6	-	light responsiveness
Ghir_D12G002680.1	591	6	+	light responsiveness
Ghir_D12G002680.1	1602	6	-	light responsiveness
Ghir_D12G002680.1	748	5	-	abscisic acid responsiveness
Ghir_D12G002680.1	1166	5	+	abscisic acid responsiveness
Ghir_D12G002680.1	748	6	+	light responsiveness
Ghir_D12G002680.1	1165	6	-	light responsiveness
Ghir_D12G002680.1	835	9	+	salicylic acid responsiveness
Ghir_D12G002680.1	1724	9	+	salicylic acid responsiveness
Ghir_D12G002680.1	916	7	-	light responsiveness
Ghir_D12G002680.1	1301	6	-	anaerobic induction
Ghir_D12G002680.1	1873	6	-	anaerobic induction
Ghir_D12G002680.1	54	6	+	light responsiveness
Ghir_D12G002680.1	147	6	+	light responsiveness

Ghir_D12G002680.1	179	6	+	light responsiveness
Ghir_D12G002680.1	361	6	+	light responsiveness
Ghir_D12G002680.1	17	6	-	low-temperature responsiveness
Ghir_D12G002680.1	563	6	+	low-temperature responsiveness
Ghir_D12G002680.1	1163	8	+	light responsiveness
Ghir_D12G002680.1	1209	9	-	light responsiveness
Ghir_D12G002680.1	1355	6	+	drought inducibility
Ghir_D13G003680.1	781	9	+	salicylic acid responsiveness
Ghir_D13G003680.1	895	7	+	gibberellin responsiveness
Ghir_D13G003680.1	109	8	+	abscisic acid responsiveness
Ghir_D13G003680.1	110	5	+	abscisic acid responsiveness
Ghir_D13G003680.1	126	10	+	defense and stress responsiveness
Ghir_D13G003680.1	1354	9	+	defense and stress responsiveness
Ghir_D13G003680.1	1700	10	-	light responsiveness
Ghir_D13G003680.1	52	10	-	light responsiveness
Ghir_D13G003680.1	1576	6	-	light responsiveness
Ghir_D13G003680.1	107	9	-	light responsiveness
Ghir_D13G003680.1	130	6	+	light responsiveness
Ghir_D13G003680.1	1201	6	-	auxin responsiveness
Ghir_D13G003680.1	109	6	+	light responsiveness
Ghir_D13G003680.1	1727	9	-	light responsiveness
Ghir_D13G003680.1	489	10	+	light responsiveness
Ghir_D13G003680.1	989	6	+	light responsiveness
Ghir_D13G003680.1	518	6	+	low-temperature responsiveness
Ghir_D13G003680.1	937	6	-	anaerobic induction
Ghir_D13G003680.1	1314	6	-	anaerobic induction
Ghir_D13G003680.1	1522	6	+	anaerobic induction
Ghir_D13G003680.1	1572	6	-	anaerobic induction
Ghir_D13G003680.1	1867	6	-	anaerobic induction
Ghir_D13G009990.1	1489	8	+	light responsiveness
Ghir_D13G009990.1	824	6	-	anaerobic induction
Ghir_D13G009990.1	1400	6	+	light responsiveness
Ghir_D13G009990.1	1010	8	+	light responsiveness
Ghir_D13G009990.1	931	6	+	light responsiveness
Ghir_D13G009990.1	1401	5	+	abscisic acid responsiveness
Ghir_D13G009990.1	956	9	+	light responsiveness
Ghir_D13G009990.1	1979	6	-	light responsiveness
Ghir_D13G009990.1	211	6	+	light responsiveness
Ghir_D13G009990.1	440	6	+	light responsiveness
Ghir_D13G009990.1	1919	6	+	light responsiveness

Ghir_D13G009990.1	1946	9	+	salicylic acid responsiveness
Ghir_D13G009990.1	1808	8	-	light responsiveness
Ghir_D13G016290.1	415	8	+	light responsiveness
Ghir_D13G016290.1	805	6	+	light responsiveness
Ghir_D13G016290.1	885	6	+	light responsiveness
Ghir_D13G016290.1	1646	6	-	light responsiveness
Ghir_D13G016290.1	1695	6	-	light responsiveness
Ghir_D13G016290.1	1467	6	-	light responsiveness
Ghir_D13G016290.1	1917	6	-	anaerobic induction
Ghir_D13G016290.1	125	5	+	MeJA responsiveness
Ghir_D13G016290.1	1931	5	+	MeJA responsiveness
Ghir_D13G016290.1	1134	8	+	light responsiveness
Ghir_D13G016290.1	1467	6	-	light responsiveness
Ghir_D13G016290.1	1467	6	-	abscisic acid responsiveness
Ghir_D13G016290.1	1468	5	+	abscisic acid responsiveness
Ghir_D13G016290.1	125	5	-	MeJA responsiveness
Ghir_D13G016290.1	1931	5	-	MeJA responsiveness
Ghir_D13G016290.1	1339	6	+	light responsiveness
Ghir_D13G016290.1	1409	6	-	light responsiveness
Ghir_D13G016290.1	1721	9	+	light responsiveness

Table S4d. cis elements in Gr RF2 genes

XP_012436227.1	1128	6	-	auxin responsiveness
XP_012436227.1	1590	6	-	light responsiveness
XP_012436227.1	170	9	-	light responsiveness
XP_012436227.1	1161	8	+	light responsiveness
XP_012436227.1	1724	9	+	salicylic acid responsiveness
XP_012436227.1	749	6	+	light responsiveness
XP_012436227.1	1163	6	-	light responsiveness
XP_012436227.1	1207	9	-	light responsiveness
XP_012436227.1	1353	6	+	drought inducibility
XP_012436227.1	504	6	-	light responsiveness
XP_012436227.1	594	6	+	light responsiveness
XP_012436227.1	1602	6	-	light responsiveness
XP_012436227.1	1299	6	-	anaerobic induction
XP_012436227.1	1873	6	-	anaerobic induction
XP_012436227.1	38	6	-	low-temperature responsiveness
XP_012436227.1	566	6	+	low-temperature responsiveness
XP_012436227.1	75	6	+	light responsiveness
XP_012436227.1	167	6	+	light responsiveness
XP_012436227.1	196	6	+	light responsiveness
XP_012436227.1	362	6	+	light responsiveness
XP_012436227.1	749	5	-	abscisic acid responsiveness
XP_012436227.1	1164	5	+	abscisic acid responsiveness
XP_012436227.1	914	7	-	light responsiveness
XP_012436227.1	1691	7	+	gibberellin responsiveness
XP_012441813.1	958	6	+	light responsiveness
XP_012441813.1	1195	6	-	light responsiveness
XP_012441813.1	1384	5	+	abscisic acid responsiveness
XP_012441813.1	1473	8	+	light responsiveness
XP_012441813.1	983	9	+	light responsiveness
XP_012441813.1	1039	8	+	light responsiveness
XP_012441813.1	850	6	-	anaerobic induction
XP_012441813.1	1448	10	+	light responsiveness
XP_012441813.1	15	6	-	drought inducibility
XP_012441813.1	1979	6	-	light responsiveness
XP_012441813.1	1812	8	-	light responsiveness
XP_012441813.1	238	6	+	light responsiveness
XP_012441813.1	1919	6	+	light responsiveness
XP_012441813.1	1946	9	+	salicylic acid responsiveness
XP_012441813.1	1383	6	+	light responsiveness

XP_012446799.1	119	6	+	light responsiveness
XP_012446799.1	1554	6	-	light responsiveness
XP_012446799.1	509	7	-	gibberellin responsiveness
XP_012446799.1	478	5	+	MeJA responsiveness
XP_012446799.1	1780	5	+	MeJA responsiveness
XP_012446799.1	1957	5	+	MeJA responsiveness
XP_012446799.1	1651	8	+	light responsiveness
XP_012446799.1	285	9	-	salicylic acid responsiveness
XP_012446799.1	637	6	+	light responsiveness
XP_012446799.1	1847	6	+	light responsiveness
XP_012446799.1	1202	6	+	drought inducibility
XP_012446799.1	810	10	-	light responsiveness
XP_012446799.1	815	10	-	light responsiveness
XP_012446799.1	496	9	-	light responsiveness
XP_012446799.1	547	6	+	light responsiveness
XP_012446799.1	1267	9	-	light responsiveness
XP_012446799.1	719	6	+	anaerobic induction
XP_012446799.1	1969	6	+	low-temperature responsiveness
XP_012446799.1	1764	13	-	light responsiveness
XP_012446799.1	454	6	+	light responsiveness
XP_012446799.1	1095	6	-	light responsiveness
XP_012446799.1	1120	6	-	light responsiveness
XP_012446799.1	1664	6	-	light responsiveness
XP_012446799.1	74	7	+	light responsiveness
XP_012446799.1	1777	8	-	auxin responsiveness
XP_012446799.1	1848	5	+	abscisic acid responsiveness
XP_012446799.1	478	5	-	MeJA responsiveness
XP_012446799.1	1780	5	-	MeJA responsiveness
XP_012446799.1	1957	5	-	MeJA responsiveness
XP_012447231.1	781	8	+	light responsiveness
XP_012447231.1	102	5	-	MeJA responsiveness
XP_012447231.1	1869	9	+	salicylic acid responsiveness
XP_012447231.1	1914	6	+	low-temperature responsiveness
XP_012447231.1	226	6	+	light responsiveness
XP_012447231.1	589	6	+	light responsiveness
XP_012447231.1	752	6	+	light responsiveness
XP_012447231.1	795	6	+	light responsiveness
XP_012447231.1	1041	6	-	light responsiveness
XP_012447231.1	1402	6	-	light responsiveness
XP_012447231.1	102	5	+	MeJA responsiveness

XP_012447231.1	1550	7	-	gibberellin responsiveness
XP_012447231.1	759	9	-	light responsiveness
XP_012447231.1	1297	9.5	-	light responsiveness
XP_012447231.1	1758	8	+	light responsiveness
XP_012447231.1	64	6	-	anaerobic induction
XP_012447231.1	565	6	-	anaerobic induction
XP_012447231.1	1529	6	+	anaerobic induction
XP_012447231.1	1594	6	-	anaerobic induction
XP_012447231.1	1791	6	+	anaerobic induction
XP_012449361.1	1778	13	-	light responsiveness
XP_012449361.1	1345	6	-	anaerobic induction
XP_012449361.1	1676	6	-	anaerobic induction
XP_012449361.1	604	6	+	light responsiveness
XP_012449361.1	649	6	+	light responsiveness
XP_012449361.1	672	6	+	light responsiveness
XP_012449361.1	676	6	+	light responsiveness
XP_012449361.1	984	6	+	light responsiveness
XP_012449361.1	1275	6	-	light responsiveness
XP_012449361.1	1606	6	-	light responsiveness
XP_012449361.1	1098	6	-	low-temperature responsiveness
XP_012449361.1	1985	7	-	gibberellin responsiveness
XP_012449361.1	1449	6	+	auxin responsiveness
XP_012449361.1	909	6	-	light responsiveness
XP_012449361.1	239	9	+	light responsiveness
XP_012449361.1	346	6	+	light responsiveness
XP_012449361.1	1083	6	+	light responsiveness
XP_012449361.1	971	10	-	light responsiveness
XP_012456530.1	65	6	+	anaerobic induction
XP_012456530.1	343	6	+	anaerobic induction
XP_012456530.1	640	6	+	anaerobic induction
XP_012456530.1	970	6	+	anaerobic induction
XP_012456530.1	1542	9	-	wound responsiveness
XP_012456530.1	1484	6	+	low-temperature responsiveness
XP_012456530.1	773	5	-	abscisic acid responsiveness
XP_012456530.1	1076	5	+	abscisic acid responsiveness
XP_012456530.1	280	5	+	MeJA responsiveness
XP_012456530.1	675	9	+	light responsiveness
XP_012456530.1	777	6	-	light responsiveness
XP_012456530.1	785	6	-	auxin responsiveness
XP_012456530.1	280	5	-	MeJA responsiveness

XP_012456530.1	1073	8	+	light responsiveness
XP_012456530.1	1354	9	+	salicylic acid responsiveness
XP_012456530.1	1662	9	-	salicylic acid responsiveness
XP_012456530.1	773	6	+	light responsiveness
XP_012456530.1	1075	6	-	light responsiveness
XP_012456530.1	1289	9	+	light responsiveness
XP_012456530.1	1522	6	+	drought inducibility
XP_012456653.1	733	9	-	salicylic acid responsiveness
XP_012456653.1	1758	6	-	light responsiveness
XP_012456653.1	1756	9	+	light responsiveness
XP_012456653.1	1855	6	-	auxin responsiveness
XP_012456653.1	570	6	+	light responsiveness
XP_012456653.1	657	7	+	light responsiveness
XP_012456653.1	1232	6	+	light responsiveness
XP_012456653.1	1435	6	-	light responsiveness
XP_012456653.1	1740	8	+	light responsiveness
XP_012456653.1	313	6	+	drought inducibility
XP_012456653.1	266	6	+	anaerobic induction
XP_012456653.1	552	8	+	light responsiveness
XP_012456653.1	1842	8	+	light responsiveness
XP_012456653.1	717	9	+	abscisic acid responsiveness
XP_012456653.1	1756	8	-	abscisic acid responsiveness
XP_012456653.1	1758	5	-	abscisic acid responsiveness
XP_012456653.1	681	6	+	light responsiveness
XP_012456653.1	784	6	+	light responsiveness
XP_012456653.1	994	6	+	light responsiveness
XP_012456653.1	1088	6	-	light responsiveness
XP_012456653.1	1320	6	-	light responsiveness
XP_012456653.1	1700	6	-	light responsiveness
XP_012456653.1	446	7	-	light responsiveness
XP_012457700.1	147	6	+	light responsiveness
XP_012457700.1	379	6	+	light responsiveness
XP_012457700.1	582	6	+	light responsiveness
XP_012457700.1	784	6	+	light responsiveness
XP_012457700.1	800	6	+	light responsiveness
XP_012457700.1	7	7	+	light responsiveness
XP_012457700.1	1125	7	-	gibberellin responsiveness
XP_012457700.1	1605	6	-	abscisic acid responsiveness
XP_012457700.1	1606	5	+	abscisic acid responsiveness
XP_012457700.1	1013	7	+	light responsiveness

XP_012457700.1	394	6	+	anaerobic induction
XP_012457700.1	1045	6	-	anaerobic induction
XP_012457700.1	1605	6	-	light responsiveness
XP_012457700.1	448	6	+	light responsiveness
XP_012457700.1	1605	6	-	light responsiveness
XP_012457700.1	1868	6	-	light responsiveness
XP_012460397.1	1104	6	-	light responsiveness
XP_012460397.1	1127	6	-	auxin responsiveness
XP_012460397.1	1886	6	-	auxin responsiveness
XP_012460397.1	34	9	+	light responsiveness
XP_012460397.1	1487	6	+	light responsiveness
XP_012460397.1	1147	9	+	salicylic acid responsiveness
XP_012460397.1	975	5	+	MeJA responsiveness
XP_012460397.1	168	8	+	light responsiveness
XP_012460397.1	1307	6	+	light responsiveness
XP_012460397.1	1702	8	+	light responsiveness
XP_012460397.1	907	6	-	drought inducibility
XP_012460397.1	1303	9	+	defense and stress responsiveness
XP_012460397.1	585	6	+	light responsiveness
XP_012460397.1	975	5	-	MeJA responsiveness
XP_012460397.1	1488	5	+	abscisic acid responsiveness
XP_012460807.1	1373	6	-	light responsiveness
XP_012460807.1	1049	6	-	light responsiveness
XP_012460807.1	1609	8	-	light responsiveness
XP_012460807.1	825	6	-	light responsiveness
XP_012460807.1	824	5	-	MeJA responsiveness
XP_012460807.1	186	7	-	light responsiveness
XP_012460807.1	187	6	-	light responsiveness
XP_012460807.1	328	7	-	light responsiveness
XP_012460807.1	329	6	-	light responsiveness
XP_012460807.1	840	6	+	light responsiveness
XP_012460807.1	1026	6	+	light responsiveness
XP_012460807.1	808	8	-	light responsiveness
XP_012460807.1	709	8	+	light responsiveness
XP_012460807.1	395	7	+	abscisic acid responsiveness
XP_012460807.1	826	5	+	abscisic acid responsiveness
XP_012460807.1	824	5	+	MeJA responsiveness
XP_012460807.1	344	6	+	light responsiveness
XP_012460807.1	575	6	+	light responsiveness
XP_012460807.1	950	6	+	light responsiveness

XP_012460807.1	554	9	-	circadian control
XP_012460807.1	1836	6	-	anaerobic induction
XP_012460807.1	1612	8	+	light responsiveness
XP_012460807.1	1035	9	-	defense and stress responsiveness
XP_012460807.1	1373	9	-	defense and stress responsiveness
XP_012463962.1	504	6	+	low-temperature responsiveness
XP_012463962.1	990	6	+	light responsiveness
XP_012463962.1	121	5	-	MeJA responsiveness
XP_012463962.1	896	7	+	gibberellin responsiveness
XP_012463962.1	134	9	+	defense and stress responsiveness
XP_012463962.1	1355	9	+	defense and stress responsiveness
XP_012463962.1	1700	10	-	light responsiveness
XP_012463962.1	938	6	-	anaerobic induction
XP_012463962.1	1315	6	-	anaerobic induction
XP_012463962.1	1523	6	+	anaerobic induction
XP_012463962.1	1572	6	-	anaerobic induction
XP_012463962.1	1867	6	-	anaerobic induction
XP_012463962.1	60	10	-	light responsiveness
XP_012463962.1	138	6	+	light responsiveness
XP_012463962.1	1576	6	-	light responsiveness
XP_012463962.1	1202	6	-	auxin responsiveness
XP_012463962.1	121	5	+	MeJA responsiveness
XP_012463962.1	1727	9	-	light responsiveness
XP_012464256.1	470	8	+	light responsiveness
XP_012464256.1	74	6	+	anaerobic induction
XP_012464256.1	1917	6	-	anaerobic induction
XP_012464256.1	490	6	+	low-temperature responsiveness
XP_012464256.1	837	6	+	light responsiveness
XP_012464256.1	917	6	+	light responsiveness
XP_012464256.1	1691	6	-	light responsiveness
XP_012464256.1	1471	6	-	abscisic acid responsiveness
XP_012464256.1	1472	5	+	abscisic acid responsiveness
XP_012464256.1	179	5	+	MeJA responsiveness
XP_012464256.1	1931	5	+	MeJA responsiveness
XP_012464256.1	1354	6	+	light responsiveness
XP_012464256.1	179	5	-	MeJA responsiveness
XP_012464256.1	1931	5	-	MeJA responsiveness
XP_012464256.1	1471	6	-	light responsiveness
XP_012464256.1	1471	6	-	light responsiveness
XP_012464256.1	1717	9	+	light responsiveness

XP_012464256.1	45	9	+	light responsiveness
XP_012464256.1	1146	8	+	light responsiveness
XP_012464256.1	1413	6	-	light responsiveness
XP_012478118.1	219	7	-	light responsiveness
XP_012478118.1	233	8	+	auxin responsiveness
XP_012478118.1	383	5	+	abscisic acid responsiveness
XP_012478118.1	233	5	+	MeJA responsiveness
XP_012478118.1	134	6	+	light responsiveness
XP_012478118.1	1342	6	+	anaerobic induction
XP_012478118.1	1954	9.5	-	light responsiveness
XP_012478118.1	623	8	-	light responsiveness
XP_012478118.1	1495	6	+	drought inducibility
XP_012478118.1	1983	6	-	drought inducibility
XP_012478118.1	1938	8	-	light responsiveness
XP_012478118.1	828	6	+	light responsiveness
XP_012478118.1	1699	6	-	light responsiveness
XP_012478118.1	382	6	-	light responsiveness
XP_012478118.1	233	5	-	MeJA responsiveness
XP_012478118.1	1255	6	+	light responsiveness
XP_012478118.1	1897	6	-	light responsiveness
XP_012480294.1	1123	6	+	low-temperature responsiveness
XP_012480294.1	1944	6	-	light responsiveness
XP_012480294.1	534	7	-	light responsiveness
XP_012480294.1	1670	7	-	light responsiveness
XP_012480294.1	1599	7	+	abscisic acid responsiveness
XP_012480294.1	147	5	-	MeJA responsiveness
XP_012480294.1	1535	6	-	anaerobic induction
XP_012480294.1	1710	6	-	anaerobic induction
XP_012480294.1	1853	6	-	anaerobic induction
XP_012480294.1	838	10	-	light responsiveness
XP_012480294.1	1667	7	+	light responsiveness
XP_012480294.1	844	6	+	light responsiveness
XP_012480294.1	43	6	-	auxin responsiveness
XP_012480294.1	6	6	-	light responsiveness
XP_012480294.1	147	5	+	MeJA responsiveness
XP_012480294.1	574	9	-	salicylic acid responsiveness
XP_012480294.1	862	9	+	salicylic acid responsiveness
XP_012480421.1	1721	8	-	light responsiveness
XP_012480421.1	1899	8	-	light responsiveness
XP_012480421.1	1275	6	-	light responsiveness

XP_012480421.1	1386	9	-	salicylic acid responsiveness
XP_012480421.1	1586	6	+	light responsiveness
XP_012480421.1	999	5	+	MeJA responsiveness
XP_012480421.1	1961	6	+	light responsiveness
XP_012480421.1	927	8	-	light responsiveness
XP_012480421.1	1276	5	+	abscisic acid responsiveness
XP_012480421.1	1587	5	+	abscisic acid responsiveness
XP_012480421.1	999	5	-	MeJA responsiveness
XP_012480421.1	45	6	+	light responsiveness
XP_012480421.1	69	6	+	light responsiveness
XP_012480421.1	153	6	+	light responsiveness
XP_012480421.1	718	6	+	light responsiveness
XP_012480421.1	728	6	+	light responsiveness
XP_012480421.1	1240	6	-	light responsiveness
XP_012480421.1	221	6	+	low-temperature responsiveness
XP_012480421.1	303	6	+	low-temperature responsiveness
XP_012480421.1	727	13	+	light responsiveness
XP_012480421.1	687	6	+	anaerobic induction
XP_012480421.1	1052	6	+	anaerobic induction
XP_012480421.1	1077	9	+	light responsiveness
XP_012481534.1	1737	9	+	salicylic acid responsiveness
XP_012481534.1	373	6	+	light responsiveness
XP_012481534.1	1047	5	-	MeJA responsiveness
XP_012481534.1	225	6	-	auxin responsiveness
XP_012481534.1	60	6	-	light responsiveness
XP_012481534.1	553	6	+	light responsiveness
XP_012481534.1	1928	8	-	light responsiveness
XP_012481534.1	857	6	+	light responsiveness
XP_012481534.1	1344	6	-	light responsiveness
XP_012481534.1	1388	6	-	light responsiveness
XP_012481534.1	569	10	+	light responsiveness
XP_012481534.1	373	6	+	light responsiveness
XP_012481534.1	373	6	+	abscisic acid responsiveness
XP_012481534.1	374	5	+	abscisic acid responsiveness
XP_012481534.1	1047	5	+	MeJA responsiveness
XP_012481534.1	26	6	+	light responsiveness
XP_012481534.1	51	6	+	light responsiveness
XP_012481534.1	1000	6	-	light responsiveness
XP_012481534.1	1020	6	-	light responsiveness
XP_012481534.1	1293	6	-	light responsiveness

XP_012481534.1	1511	6	-	light responsiveness
XP_012481534.1	1618	6	-	light responsiveness
XP_012481534.1	890	6	+	low-temperature responsiveness
XP_012487039.1	1185	6	-	light responsiveness
XP_012487039.1	1585	7	+	light responsiveness
XP_012487039.1	671	6	+	low-temperature responsiveness
XP_012487039.1	852	6	-	low-temperature responsiveness
XP_012487039.1	1969	6	-	low-temperature responsiveness
XP_012487039.1	24	5	+	abscisic acid responsiveness
XP_012487039.1	174	6	+	abscisic acid responsiveness
XP_012487039.1	175	5	+	abscisic acid responsiveness
XP_012487039.1	715	7	+	abscisic acid responsiveness
XP_012487039.1	886	9	-	abscisic acid responsiveness
XP_012487039.1	888	6	+	abscisic acid responsiveness
XP_012487039.1	889	5	+	abscisic acid responsiveness
XP_012487039.1	1310	9	-	cell cycle regulation
XP_012487039.1	898	6	+	anaerobic induction
XP_012487039.1	1907	6	-	anaerobic induction
XP_012487039.1	23	6	-	light responsiveness
XP_012487039.1	174	6	+	light responsiveness
XP_012487039.1	888	6	+	light responsiveness
XP_012487039.1	442	6	+	drought inducibility
XP_012487039.1	596	6	+	drought inducibility
XP_012487039.1	750	6	+	drought inducibility
XP_012487039.1	1455	6	+	drought inducibility
XP_012487039.1	585	9	+	light responsiveness
XP_012487039.1	584	11	+	light responsiveness
XP_012487039.1	1777	9	+	salicylic acid responsiveness
XP_012487039.1	21	8	+	light responsiveness
XP_012487039.1	174	6	+	light responsiveness
XP_012487039.1	789	9	+	light responsiveness
XP_012487039.1	888	6	+	light responsiveness
XP_012488308.1	120	5	-	MeJA responsiveness
XP_012488308.1	977	5	+	MeJA responsiveness
XP_012488308.1	1151	5	+	MeJA responsiveness
XP_012488308.1	1359	5	+	MeJA responsiveness
XP_012488308.1	538	6	+	light responsiveness
XP_012488308.1	627	6	+	light responsiveness
XP_012488308.1	1765	6	-	light responsiveness
XP_012488308.1	1769	6	-	light responsiveness

XP_012488308.1	217	13	-	light responsiveness
XP_012488308.1	126	6	+	low-temperature responsiveness
XP_012488308.1	190	6	+	low-temperature responsiveness
XP_012488308.1	1036	6	-	anaerobic induction
XP_012488308.1	1511	8	-	light responsiveness
XP_012488308.1	135	9	-	light responsiveness
XP_012488308.1	1465	7	+	light responsiveness
XP_012488308.1	1725	7	+	light responsiveness
XP_012488308.1	176	6	+	drought inducibility
XP_012488308.1	120	5	+	MeJA responsiveness
XP_012488308.1	977	5	-	MeJA responsiveness
XP_012488308.1	1151	5	-	MeJA responsiveness
XP_012488308.1	1359	5	-	MeJA responsiveness
XP_012488308.1	165	7	-	gibberellin responsiveness
XP_012488308.1	1960	7	-	light responsiveness
XP_012488308.1	1961	6	-	light responsiveness
